# The dark side of the light (UVA): melanoma microenvironment and cell survival strategies

**DOI:** 10.1038/s41420-025-02751-y

**Published:** 2025-10-20

**Authors:** Abhijit Basu, Peters Thorsten, Björn Schumacher, Dimitris Kletsas, Karin Scharffetter-Kochanek

**Affiliations:** 1 Experimental Laboratories of the Department of Dermatology and Allergic Diseases, Ulm University, Ulm, Germany; 2 Department of Dermatology and Allergic Diseases, University Hospital, Ulm University, Ulm, Germany; 3 Institute for Genome Stability in Ageing and Disease, Medical Faculty and Cologne. Excellence Cluster for Cellular Stress Responses in Ageing-associated Diseases (CECAD). Research, University of Cologne, Cologne, Germany; 4 Laboratory of Cell Proliferation & Ageing, Institute of Bioscience and Application, National Center for Scientific Research Demokritos, Athens, Greece

**Keywords:** Cancer microenvironment, Melanoma

## Abstract

The incidence of cutaneous melanoma is rapidly escalating in many developed countries, particularly among the aging populations. Extensive evidence shows epigenetic changes have a strong correlation between cutaneous malignant melanoma and exposure to sunlight, particularly its ultraviolet (UV) components, in the aging skin. While significant research has been conducted on aging and its associated Senescence-Associated Secretory Phenotype (SASP) contributed by senescent fibroblasts in old individuals, less is known about the role of UVA radiation in such SASP melanoma microenvironment, its effects on gene function, and the underlying mechanisms following UVA-induced DNA Damage (UDD). It is established that UVA radiation induces Double-Strand Breaks (DSBs) in DNA and activates checkpoint kinase 2 (Chk2) at these breaks, leading to p53-mediated apoptosis. But p53, beyond its role in regulating cell fate, its mutated form is also involved in the transcriptional regulation of potent pro-survival pathways by actively transcribing genes that counteract apoptosis in genetically abnormal melanoma cells. However, the molecular mechanisms that govern the fine balance between cell death and survival mediated by the p53 transcription factor under UVA exposure remain largely elusive. In this study, we report for the first time that UVA induces global DNA methylation as a compensatory mechanism in response to UVA-induced DNA Damage (UDD) in melanoma and melanocyte cells. However, melanoma cells in the vicinity of senescent fibroblasts under genotoxic stress are epigenetically altered by the paracrine secretion of interleukin-6 (IL-6) from senescent fibroblasts, which upregulates the anti-apoptotic gene *GDF-15* as well as the DNA damage repair system. This upregulation occurs via hypomethylation of *GDF-15* orphan CpG island promoters, followed by its active gene transcription of *GDF-15* via both WT p53^Ser392^ and Mutated p53^N239Y^ transcription factors, thereby enhancing pro-survival mechanisms that contribute to melanoma progression.

## Introduction

For the past decade, UVA (320–400 nm) has been identified as a significant risk factor in the development of both cutaneous melanoma [1–3] and non-melanoma skin cancers, especially in old patients [4–7]. Beyond its association with oxidative damage, particularly the formation of 8-oxo-7,8-dihydroguanine (8-oxoGua) and cyclobutane pyrimidine dimers (CPDs), especially the CPDs that lead to mutations in key tumor suppressor genes such as p53, melanoma cells exhibit epigenetic mechanisms linked to defective or impaired nucleotide excision repair (NER) systems [8], a topic that has not been thoroughly explored. In this study, we demonstrate that under UVA irradiation, melanoma cells adopt a multifaceted response to genotoxic stress, opting not only for cell cycle arrest or apoptosis, but also introducing a third mechanism: the induction of CpG hypermethylation in the promoters of critical cell survival genes. Although hypomethylation of the Aging non-malignant skin has been previously reported with UVA irradiation [9, 10]. Notably, we reveal for the first time that high levels of UVA irradiation led to a downregulation of interleukin-6 (IL-6) in A375 melanoma cells when compared to the non-irradiated control. However, in the presence of a supportive microenvironment provided by UVA-exposed senescent fibroblasts, independent of whether it derived from natural sun-exposed skin, tanning beds [11] or through in vitro co-culture, it fathoms a stable and elevated paracrine IL-6 around the melanoma cells. This condition facilitates the hypomethylation of orphan CpG islands in the *GDF-15* gene promoter at the p53 binding sites, which, when transcribed by p53, leads to an increased GDF-15 secretion in melanoma cells, which leads to reduced apoptosis in UVA-irradiated conditions [12]. Conversely, fibroblasts, both young and senescent, exhibit reduced GDF-15 secretion despite elevated IL-6 levels following UVA exposure. Previously, UV irradiation and its role in epigenetic regulation have been reported, impacting either the methylome of epidermal cells (reviewed in de Oliveira et al. [13]), Hypomethylation in aging skin (Gröninger et al. [14], Vandiver et al. [9]), or in genome-wide promoter methylation in melanoma models (Bormann et al. 2016 [10], Shen et al. [15], Rius et al. [16]). However, the exact mechanisms of the impact of UVA irradiation on the epigenetic crosstalk between senescent fibroblasts and melanoma cells have not been addressed in sufficient detail. We here propose that the paracrine releases of increased IL-6 concentrations from senescent fibroblasts profoundly alter levels of DNA methylation in melanoma cells and elevated levels of active phosphorylated wild-type or mutated p53 transcription factor, collectively enhancing melanoma cell survival. Furthermore, our findings indicate that IL-6 phosphorylates p53 at serine 392 via the Stat2/3 pathway, facilitating the translocation of p53 into the nucleus, where it functions as a transcription factor for both mutated and wild-type p53. However, successful binding to the *GDF-15* promoter and subsequent active transcription only occur when the promoter is hypomethylated in melanoma rather than being mostly hypermethylated. In summary, UVA-irradiated senescent fibroblasts secrete IL-6 paracrinically, potentially regulating melanoma cell survival by inducing hypomethylation of the *GDF-15* gene promoter. This mechanism may assist melanoma cells in evading apoptosis under genotoxic stress conditions.

## Results

### UVA irradiated senescent fibroblast conditioned medium (CM) enhances the migration of melanoma cell lines

**Fig. 1:**
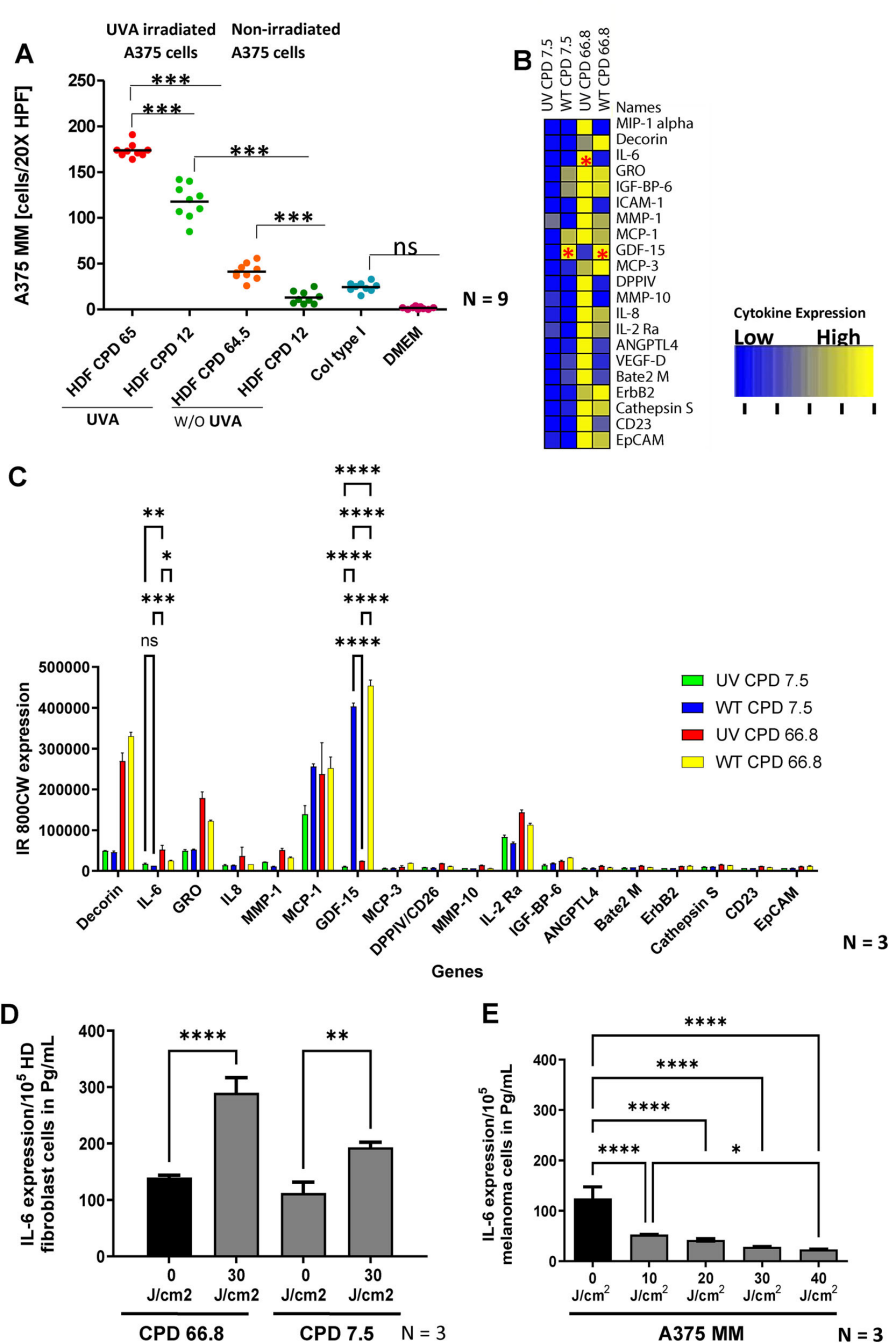
CM released by senescent fibroblasts post-irradiation confers oncogenic properties of melanoma cells. **A** CM from non-senescent fibroblasts (FF95 CPD 7.4, FFRa CPD 14.4) and senescent fibroblasts (FF95 CPD 64.6, FFRa CPD 64.6), 30 J UVA-irradiation were assessed for their stimulatory effect on non-irradiated and post-irradiated A375 melanoma cell migration. Nine technical replicates were performed. 10% FCS (Fetal Calf Serum) and collagen type 1 served as positive controls, as previously published [44]. 0.2% DMEM served as a negative control. CM from non-senescent (FF95 CPD 14.4) and senescent fibroblasts (FF95 CPD 68) were assessed for their secreted cytokine and chemokines by cytokines & chemokines profiling assay, as shown in bar graphs (**C**) and heatmap (**B**). Three technical replicates were performed. All data are mean ± s.e.m. All experiments were repeated 3 independently with two fibroblast cell lines. **D** Furthermore, confirmatory IL-6 quantification was carried out three times independently via IL-6 ELISA upon CM from non-senescent and senescent fibroblast cells, both in non-irradiated and irradiated conditions. **E** Also, dosage-wise IL-6 quantification was carried out in three independent experiments with CM from non-irradiated and post-irradiated A375 melanoma cells. *P < 0.01, ***P < 0.001, ****P < 0.0001 by ANOVA multiple comparison with *Tukey’s* posthoc test.

UVA-irradiated A375 MM melanoma cells showed significantly increased migration towards conditioned media (CM) from irradiated replicative senescent (10000 cells/plate) and young non senescent (10000 cells/plate) fibroblasts compared to non-irradiated controls (fibroblasts and melanoma cells), as demonstrated in the Transwell® chamber migration assay (Fig. 1A). Collagen served as a positive control, while 0.2% DMEM without FCS was the negative control.

### Characterization of conditioned medium (CM) from UVA-irradiated senescent fibroblasts shows enhanced IL-6 and reduced GDF-15

Characterization of conditioned media (CM) from 30 J/cm^2^ UVA-irradiated senescent and young fibroblasts revealed a significant upregulation of interleukin IL-6 in CM from UVA-irradiated senescent fibroblasts (Fig. 1B, C). The heatmap in Fig. 1B and the bar plots in Fig. 1C illustrate this finding. The fold change for IL-6 was 3.5-fold for UVA-irradiated versus 1.1-fold for non-irradiated senescent and young fibroblasts, with P values shown in Suppl. Figure 1A. Conversely, GDF-15 levels were reduced in CM from UVA-irradiated fibroblasts compared to non-irradiated counterparts. Overall, these results indicate that UVA-irradiated fibroblasts increase IL-6, while decreasing GDF-15 levels. This was further confirmed using an antibody-specific ELISA (Fig. 1D).

### Conditioned medium (CM) from UVA-irradiated melanoma cells shows a dose-dependent reduction in IL-6 and IL-6R

A375 melanoma cells exhibited reduced IL-6 secretion in a dose-dependent manner after UVA irradiation, as shown by ELISA (Fig. 1E), immunofluorescence, and western blotting ((Suppl. Figures 1B, 1C), Fig. 4A). This was accompanied by decreased IL-6R (GP130) expression, which was partially restored by 200 pg/mL recombinant IL-6 following 30 J/cm^2^ UVA exposure. To explore the effects of DNA methylation on IL-6 or IL-6R expression, we applied 0.1% methylation modulating agent methyl-methane sulfonate (MMS), 0.1 µM demethylating agent azacytidine (AZA) as controls. Co-culturing DDK-tagged IL-6-expressing fibroblasts with UVA-irradiated A375 melanoma cells led to the uptake of tagged IL-6 (Supplementary Fig. 1B). Fibroblast-derived IL-6, stained red with an anti-DDK antibody, showed clear uptake by melanoma cells after UVA exposure (Suppl. Figure 1B, lower right). In summary, UVA irradiation reduces IL-6 and IL-6R in melanoma cells, which can be partially restored by IL-6 supplementation, but not via genome-wide demethylation or methylation.

### UVA-irradiated senescent fibroblast conditioned medium (CM) rescues UVA-irradiated melanoma cells from apoptosis

**Fig. 2:**
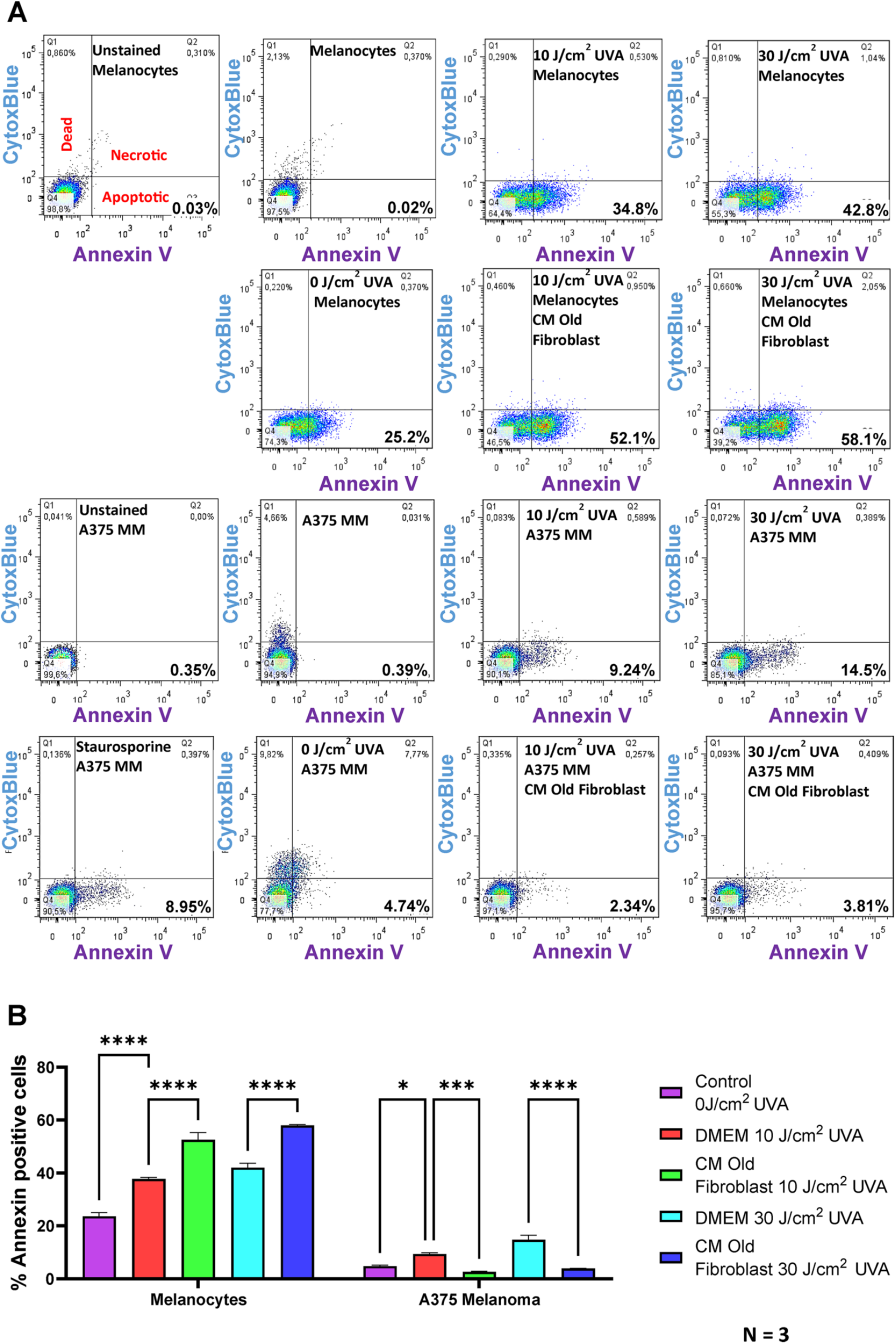
IL-6 in the CM released by senescent fibroblasts post-irradiation confers anti-apoptotic properties of melanoma cells. **A** UVA irradiated CM from senescent fibroblasts (FF95 CPD 64.6, FFRa CPD 64.6) was assessed for its anti-apoptotic effect on melanoma cells and melanocytes. CM was added to melanocytes and A375 melanoma cells, which were further subjected to 30 J/cm^2^ irradiation. Fluorescently labeled Annexin-V was used to measure percent apoptosis, and Sytox Blue/Red was used to measure percent dead/necrotic cells of total A375 MM cells. Annexin-positive cells were considered apoptotic, whereas double-labeled Annexin plus Sytox were considered late apoptotic. Staurosporine-treated cells served as a positive control. Three independent experimental replicates were performed. **B** shows the quantitative representation of the above experiment in a bar graph, separated into early and late apoptosis.

**Fig. 3:**
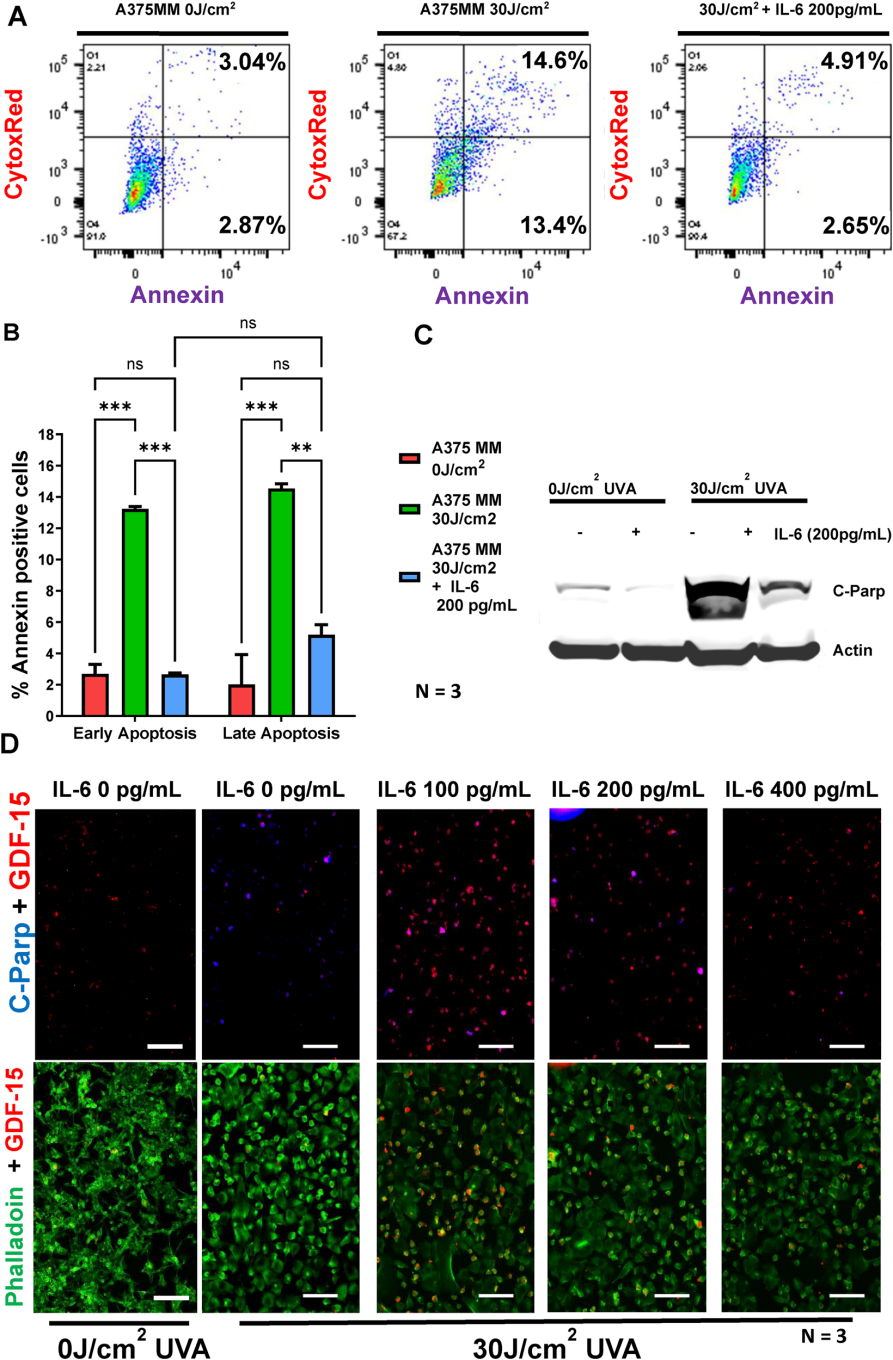
C-Parp staining confirms that IL-6 in the CM released by senescent fibroblasts post irradiation confers anti-apoptotic properties of melanoma cells. **A** To determine if IL-6 is the major component of the CM from senescent fibroblast cells, 200 pg/mL of IL-6 was supplemented with post-UVA irradiated melanoma cells. **B** The bar graph representation of 3 A. **C** Western blot showing that IL-6 supplementation on post-irradiated melanoma leads to a reduction in apoptosis marker C-Parp. **D** In a complementary approach with immunostaining with anti-C-Parp and anti-GDF-15 antibodies was used to determine the apoptosis under non-irradiated and UVA irradiated conditions in A375 melanoma cells, which were either supplemented with IL-6 or without. Non-irradiated non-treated with IL-6 served as a control.

**Fig. 4:**
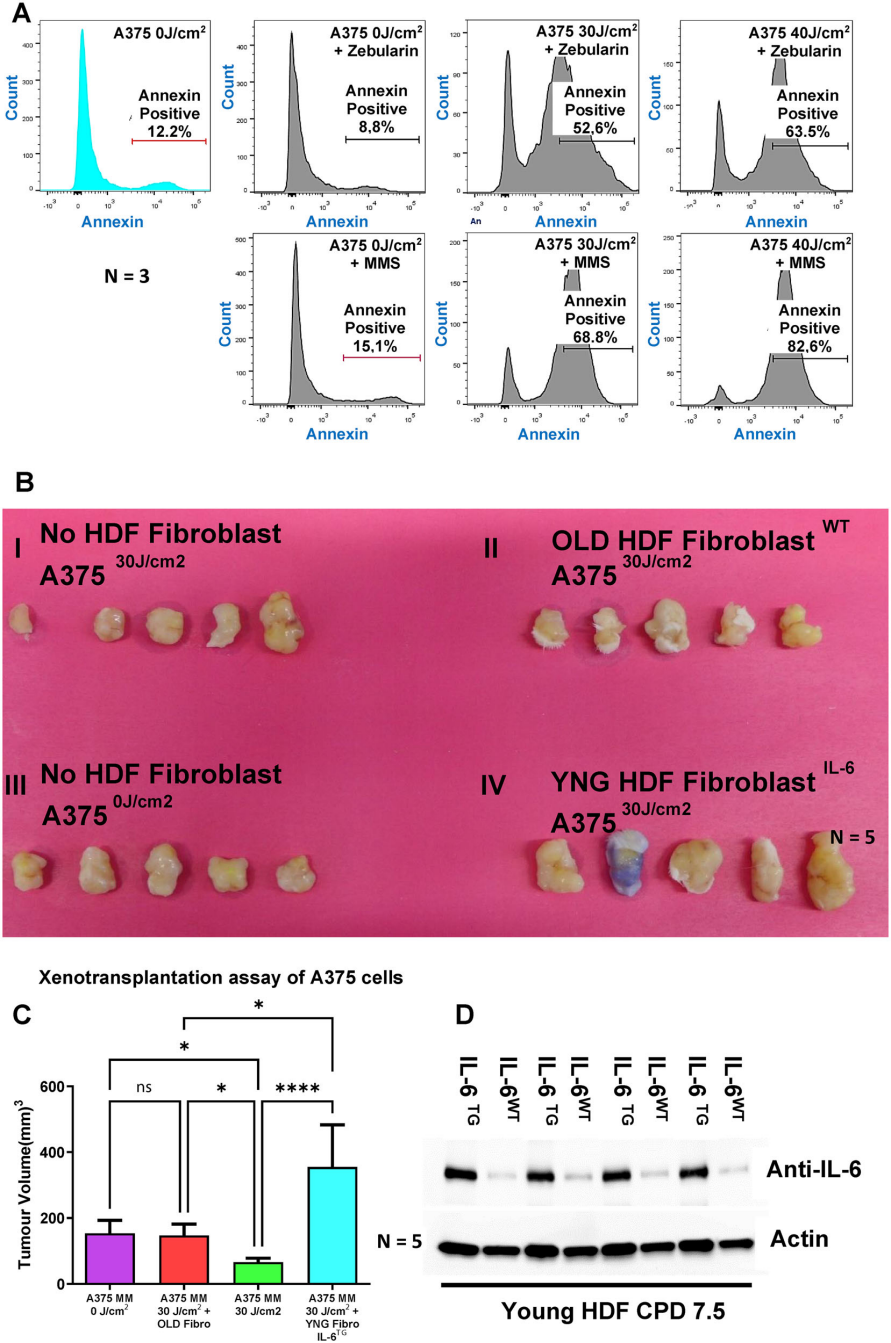
Xenotransplantation studies with IL-6-supplemented fibroblasts post-irradiation confer resistance against apoptosis in melanoma cells. **A** Supplementation of global DNA methylating agent MMS 200 pg/mL or demethylating agent AZA 200 pg/mL on UVA irradiated in vitro cultured A375 melanoma cells does not show anti-apoptotic effect (**B**). Represents the assessment of the in vivo efficacy of IL-6 overexpressing young fibroblast cells along with A375 melanoma cells in a xenotransplant experiment in SCID mice. **B**, **IV**) when mixed and co-injected with post-irradiated melanoma cells in comparison to either irradiated melanoma cells without co-injected fibroblast (**B**, **I/III**) and with senescence fibroblasts (FF95 CPD 68) (**B**, **II**). **C** Bar graph of the in vivo experiment showing quantification of the tumor volume. Young fibroblast overexpressing IL-6 showed the highest tumor volume in comparison to without fibroblast, either irradiated or non-irradiated, or with senescent fibroblast co-injection. **D** Overexpression of IL-6 from the lysate of young fibroblasts was assessed for their IL-6 cytokine levels in comparison to young wild-type fibroblasts.

30 J/cm^2^ UVA-irradiated A375 melanoma cells supplemented with 30 J/cm^2^ UVA-irradiated conditioned media (CM) from senescent fibroblasts showed a significant reduction in cell apoptosis as measured by Annexin V staining, when compared to A375 melanoma control cells supplemented with 10% DMEM. In contrast, melanocytes displayed a significantly higher rate of apoptosis when treated identically (Fig. 2A). Specifically, the CM from senescent fibroblasts reduced apoptosis in melanoma cells consistently by over 10.69%, while it increased apoptosis in melanocytes by 15.3% (Fig. 2B). To determine if IL-6 was the key factor in this apoptotic rescue, we supplemented 200 pg/mL of IL-6 instead of CM to the post irradiated melanoma cells. This led to a significant decrease in apoptosis in UVA-irradiated melanoma cells (Fig. 3A, B). Additionally, C-PARP staining & western blotting confirmed reduced apoptosis levels with recombinant IL-6 supplementation (Fig. 3C, D). Notably, global DNA methylation or demethylation using MMS or AZA at 200 pg/mL did not show any anti-apoptotic effects (Fig. 4A).

### Paracrine secretion of young fibroblasts overexpressing IL-6 imparts an increase in melanoma volume in xenograft transplantation

To investigate the functional role of fibroblast-secreted IL-6 on 30 J/cm^2^ UVA-irradiated A375 melanoma cells, we injected 10^6^ melanoma cells either non-exposed or exposed to 30 J/cm^2^ UVA, along with or without young fibroblasts or young fibroblasts constitutively overexpressing IL-6 (Fig. 4B). Young fibroblasts were selected to avoid the influence of other SASP cytokines typically released from senescent fibroblasts (Fig. 4B, I-IV). Quantitative results of melanoma volume showed that A375 melanoma cells co-injected with IL-6 overexpressing young fibroblasts survived and proliferated significantly more than those injected with senescent fibroblasts or without fibroblasts (Fig. 4C). Tumor volume was significantly greater with IL-6 overexpressed young fibroblasts compared to irradiated melanoma cells without fibroblasts. Notably, IL-6 induction in UVA-irradiated A375 melanoma cells alone was insufficient to provide enhanced protection. Western blot analysis was done to confirm the overexpression of young IL-6^TG^ fibroblasts as compared to young IL-6^WT^ fibroblasts, as shown in Fig. 4D.

### Pleotropic roles of IL-6 induce melanoma cell proliferation, DNA damage rescue, and protection against apoptosis under genotoxic stress

**Fig. 5:**
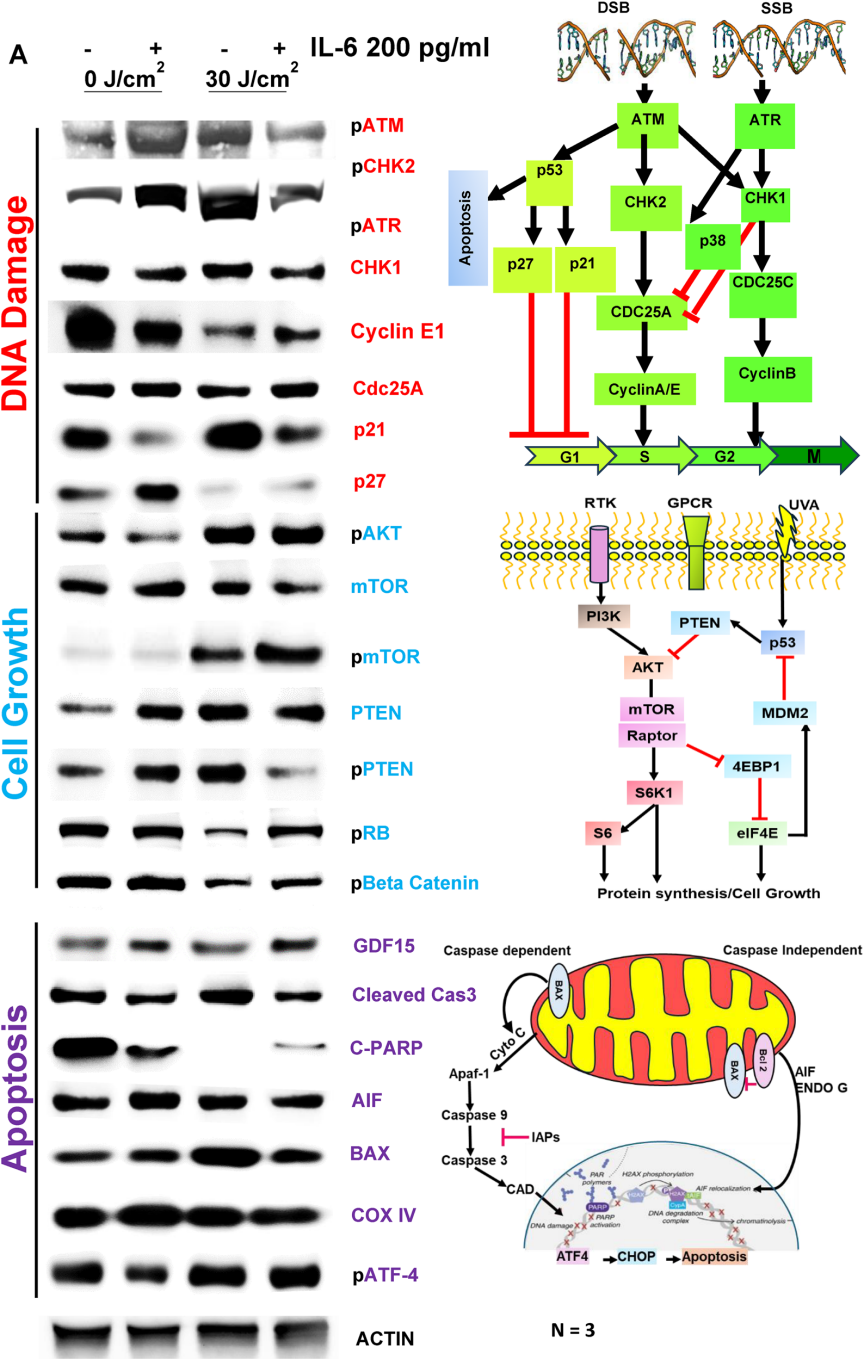
Reduction of DNA damage and apoptosis, increase in cell growth with IL-6 supplemented senescent fibroblasts post irradiation, confers anti-apoptotic properties of melanoma cells. **A** mTOR, a protein kinase that regulates cellular metabolism and proliferation, along with PTEN, a tumor suppressor that downregulates AKT, and retinoblastoma (RB), which inhibits CDKs, demonstrated increased phospho-mTOR and phospho-RB, and decreased phospho-PTEN post-UVA IL-6 supplementation, indicating a shift toward proliferation and protein synthesis. Inactive PTEN and upregulated pAKT in both pre- and post-UVA conditions with IL-6 suggest enhanced cellular proliferation compared to non-irradiated cells. Investigation of pro-apoptotic genes revealed that Caspase 3 and BAX were downregulated after UVA IL-6 supplementation in A375 melanoma cells, while C-PARP was also reduced compared to non-UVA conditions. AIF and CoxV showed minimal differences, indicating no significant caspase-independent pathway activation. Conversely, the anti-apoptotic gene GDF-15 was upregulated following UVA IL-6 supplementation compared to the control. These results suggest the activation of the ATM-mTOR-GDF-15 axis in response to IL-6 supplementation post-UVA irradiation *P < 0.01, ***P < 0.001, ****P < 0.0001 by ANOVA multiple comparison with *Tukey’s* posthoc test.

The ATM pathway is activated by DNA double-strand breaks and oxidative stress [17] while pATR responds to single-strand breaks [18], working together with ATM to maintain genome integrity. CHK1/2 is involved in DNA repair, cell cycle arrest, or apoptosis in response to DNA damage. p21, a major target of p53, links DNA damage to cell cycle arrest and degradation of Cdc25 A in response to DNA damage [19], further promoting cell cycle arrest by inhibiting CDKs. Cyclin E1 and p27Kip1 act as CDK inhibitors. After UVA irradiation, the pATM/ATM ratio was upregulated, but with IL-6 supplementation, this ratio was reduced, whereas non-irradiated A375 melanoma cells did not show such an activation by IL-6 (Fig. 5A). mTOR, a protein kinase that regulates cellular metabolism and proliferation, along with PTEN, a tumor suppressor that downregulates AKT, and retinoblastoma (RB), which inhibits CDKs, demonstrated increased phospho-mTOR and phospho-RB with UVA, but it decreased phospho-PTEN after UVA and IL-6 supplementation, indicating a shift toward proliferation and protein synthesis. Inactive PTEN and upregulated pAKT in both pre- and post-UVA conditions with IL-6 supplementation suggest enhanced cellular proliferation compared to non-irradiated melanoma cells. Investigation of pro-apoptotic genes revealed that caspase 3 and BAX were downregulated after UVA and IL-6 supplementation in A375 melanoma cells, while C-PARP was also reduced compared to non-UVA conditions. Apoptosis-inducing factors, mitochondrial (AIF) and CoxV, showed minimal differences, indicating that there is no significant activation of a caspase-independent pathway. Conversely, the gene coding for the anti-apoptotic GDF-15 protein was upregulated following UVA and IL-6 supplementation compared to the control (Unirradiated and irradiated but without IL-6). These results suggest the activation of the ATM-mTOR-GDF-15 axis in response to IL-6 supplementation after UVA-irradiation of melanoma cells.

### UVA-irradiated senescent fibroblast conditioned medium (CM) alters the methylation states of *GDF-15* gene promoter in UVA-irradiated melanoma cells

**Fig. 6:**
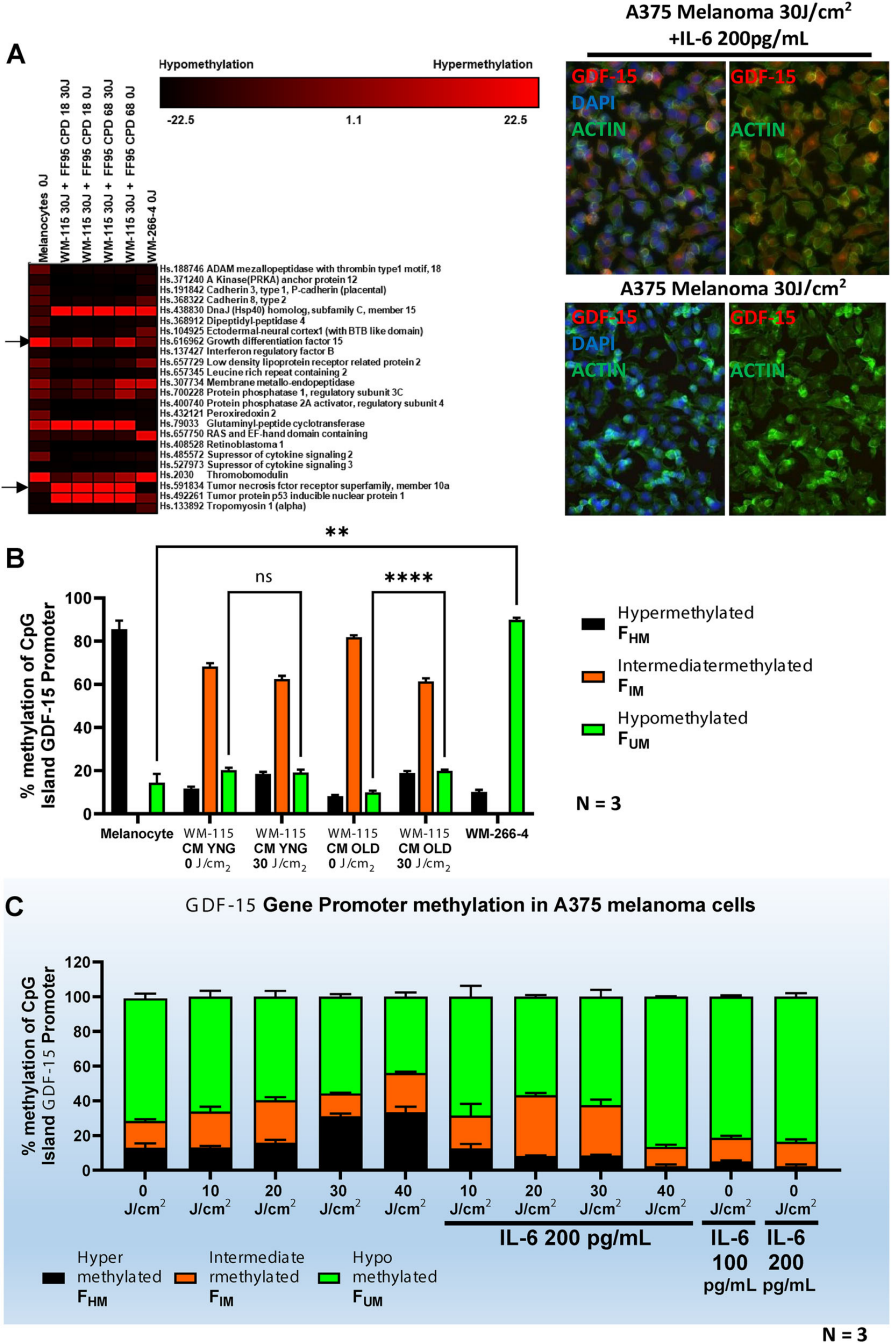
IL-6 in the CM released by senescent fibroblasts post irradiation induces selective hypomethylation in orphan CpG islands of melanoma cells. **A** Epitect® methylation array was used to assess the promoter methylation levels in primary melanoma WM-115 cells. CM from post-irradiated senescence fibroblasts (FF95 CPD 68.6, FFRa CPD 64.6) and young fibroblasts (FF95 CPD 18, FFRa CPD 7.5), and post-irradiated WM-115 melanoma was used. Here non-irradiated metastatic melanoma cells WM-226-4 and melanocyte served as positive and negative controls. Promoters were either hypermethylated (Red) or hypomethylated (Black), or Intermediatory methylated (Brown). Promoter quantification is shown in the Suppl. Table 2. We observe selective hypomethylation of many promoters in the WM-115 cell line derived from primary melanoma (**B**). Quantification of % GDF-15 CpG promoter methylation with CM from post-irradiation senescence fibroblasts on post-irradiation primary melanoma is shown, where supplementation of CM leads to hypomethylation GDF-15 CpG promoter, whereas the melanocytes promoter was hypermethylated, and metastatic melanoma WM-255-4 was hypomethylated. Here, hypermethylation of the TNF-alpha promoter was observed regardless of UVA exposure. **C** To observe if IL-6 is the principal component in the GDF-15 promoter hypomethylation, A375 metastatic melanoma cells were post-irradiated and supplemented with or without IL-6 200 pg/mL. UVA irradiation increased hypermethylation of CpG islands, whereas IL-6 supplementation reduced hypermethylation and increased hypomethylation under genotoxic stress. Non-irradiated A375 melanoma did not display a dramatic epigenetic change. **D** The outcome of the GDF-15 promoter hypomethylation is the increase in GDF-15 secretion in A375 melanoma as measured with GDF-15 ELISA assay and immunostaining (**A**, right). Post-irradiated A375 shows reduced secretion; on the contrary, GDF-15 secretion increased, followed by supplementation of IL-6. Use of global DNA methylating (MMS) or demethylating (AZA) agents did not lead to significant GDF-15 expression. Non-irradiated A375 cells with IL-6 supplementation had significantly less GDF-15 secretion. *P < 0.01, ***P < 0.001, ****P < 0.0001 by ANOVA multiple comparison with *Tukey’s* posthoc test.

Melanoma cells co-cultured with CM from 30 J/cm^2^ UVA-exposed human dermal senescent fibroblasts (HDF), which were analyzed using the Epitect® methyl PCR array, exhibited alterations in the methylation states of CpG islands across melanocytes and primary and metastatic melanoma cell lines. These included hypermethylation (FHM), intermediate methylation (FIM), and hypomethylation (FUM) (Fig. 6A and Suppl. Table 2). Notably, UVA-irradiated CM from senescent fibroblasts resulted in altered hypomethylation levels in the primary melanoma cell line WM-115. Specifically, the anti-apoptotic gene *GDF-15* showed 9% hypomethylation when incubated with CM from young, irradiated fibroblasts and 20% hypomethylation when co-cultured with CM from irradiated senescent fibroblasts. In contrast, the metastatic cell line WM-266-4 demonstrated 90% hypomethylation of *GDF-15* (Fig. 6B). For comparison, melanocytes served as controls, showing 86% hypermethylation for *GDF-15*. Taken together, CM from Senescence fibroblast alters the methylome of post-irradiated melanoma.

### Elevated IL-6 concentrations lead to selective hypomethylation of the orphan CpG *GDF-15* promoter in melanoma cell lines

**Fig. 7:**
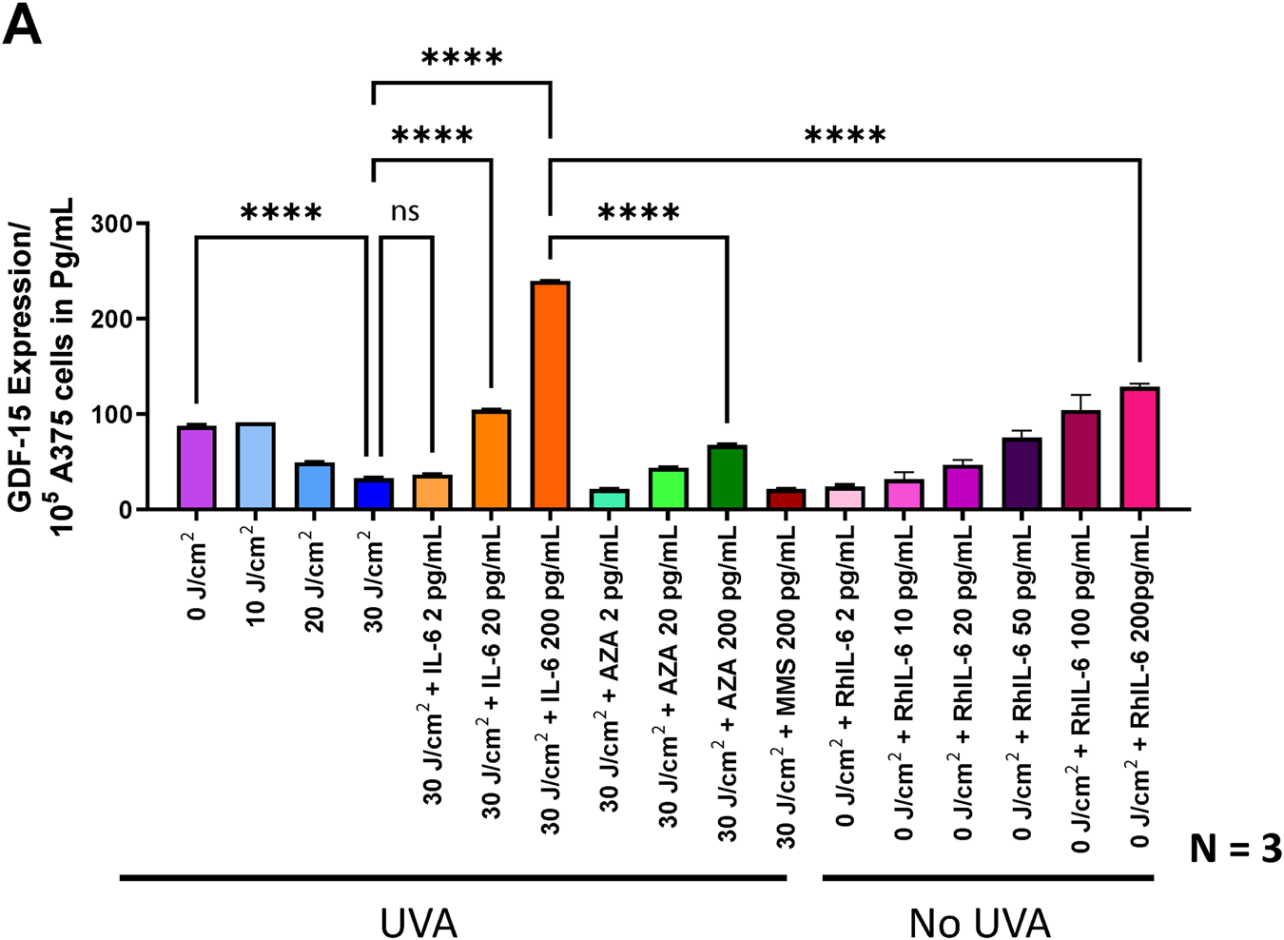
Enhanced GDF-15 expression is observed with IL-6-supplemented senescent fibroblasts post-irradiation, but not in non-irradiated melanoma cells. **A** The outcome of the GDF-15 promoter hypomethylation is the increase in GDF-15 secretion in A375 melanoma as measured with GDF-15 ELISA assay and immunostaining. *P < 0.01, ***P < 0.001, ****P < 0.0001 by ANOVA multiple comparison with *Tukey’s* posthoc test.

30 J/cm^2^ UVA irradiation and 200 pg/µl of recombinant IL-6 (RhIL-6) cause genome-wide hypermethylation of genomic DNA in melanoma cells under genotoxic stress as evaluated by 5-methylcytosine (5-MC) expression levels (Suppl. Figure 1D, E) compared to non-irradiated cells. However, analysis of methylation levels in the CpG islands of the *GDF-15* promoter by using specific biotin probes showed that elevated IL-6 concentrations reduced overall *GDF-15* methylation of post UVA irradiated (30 J/cm^2^) cells (Suppl Fig. 1F). In addition, under these experimental conditions, the CpG island promoters of *GDF-15* at p53 binding site were converted to C- > T as observed with bisulfite sequencing data (Suppl Fig. 2B). These data indicate that increasing IL-6 supplementation of UVA irradiated melanoma cells can specifically hypomethylate the *GDF-15* promoter at the p53 binding region, while UVA exposure induces in general genome wide DNA hypermethylation. Using CpG methylation-sensitive (SmaI) and-insensitive (MspI) restriction enzymes along with methylation restriction-specific PCR primers (Suppl. Fig. 2A), we found that UVA irradiation significantly increased hypermethylation in A375 melanoma cells, from over 15% at 10 J/cm^2^ to over 35% at 40 J/cm^2^. When A375 cells exposed to 10-40 J/cm^2^ UVA were treated with 200 pg/mL rhIL-6, the *GDF-15* promoter hypomethylation increased to 40% at 20 J/cm^2^ and to over 65% at 40 J/cm^2^, while hypermethylation decreased from 15% at 10 J/cm^2^ to less than 5% at 40 J/cm^2^ (Fig. 6C). Intermediate methylation levels were observed in primary melanoma cell line (WM-115) originating from primary tumor, but reduced levels of methylation were observed in A375/WM-266-4 melanoma cell line which were derived from lymphatic nodes of metastatic tumor, these were predominantly hypomethylated as observed in *GDF-15* promoter even without UVA irradiation (0 J/cm^2^). This reduction in methylation of non-irradiated melanoma cells did not change with 100 or 200 pg/mL IL-6 supplementation. *GDF-15* hypomethylation correlated with its secreted expression, which increased proportionally with IL-6 concentrations (2 to 200 pg/mL). Notably, 10-30 J/cm^2^ UVA reduced GDF-15 secretion, but IL-6 addition significantly increased it, along with some enhancement from the demethylating agent Azacytidine, as measured by ELISA and western blotting. Conversely, the methylating agent methyl methane sulfonate (MMS) reduced GDF-15 secretion (Fig. 7A). Similar results were confirmed using dot blot analysis with GDF-15 biotin-labeled probes (Suppl Fig. 1F).

### *GDF-15* Orphan CpG promoter is hypomethylated and its expression is elevated in melanoma biopsies

**Fig. 8:**
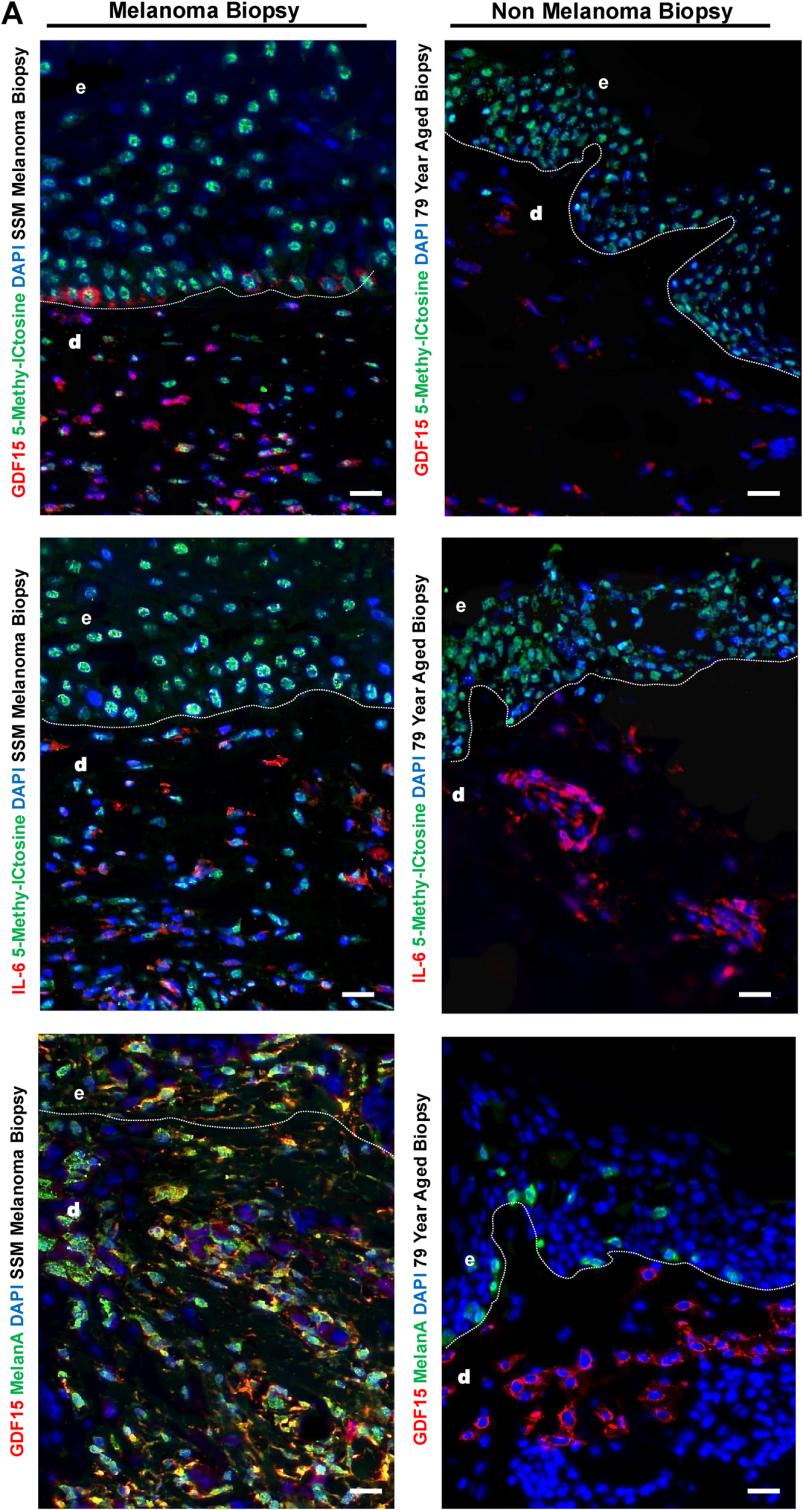
Melanoma tissue biopsy shows co-expression of GDF-15 and DNA methylation in dermal compartments in melanoma versus non-melanoma controls. Tissue sections from samples of SSM patients using anti-5MC (green) and anti-GDF15 (red) antibodies (**A** top panels). The results indicate that GDF-15 expression is much higher in melanoma biopsies as compared to healthy aged skin, as well as is also scantily hypermethylated, as shown with low 5MC expression. (**A** middle panels). Additionally, we observed that high IL-6 expression from (Red) as compared to the healthy aged skin, but was not associated with high methylation levels (Green) in the dermal compartment. Although aged skin biopsy also showed high IL-6 levels in the dermal compartment but were not in the vicinity of methylated cells. (**A** lower panels) GDF-15 expression was more prevalent in Melan A-positive metastatic melanoma cells, as shown by the co-stained yellow color, as compared to the healthy aged skin. Taken together, IL-6 induces hypomethylation in melanoma. To determine if GDF-15 expression correlates with hyper or hypomethylated regions in melanoma biopsies as compared to healthy aged biopsies, we performed immunostaining on ti cells, which are GDF-15 and MelanA positive.

**Fig. 9:**
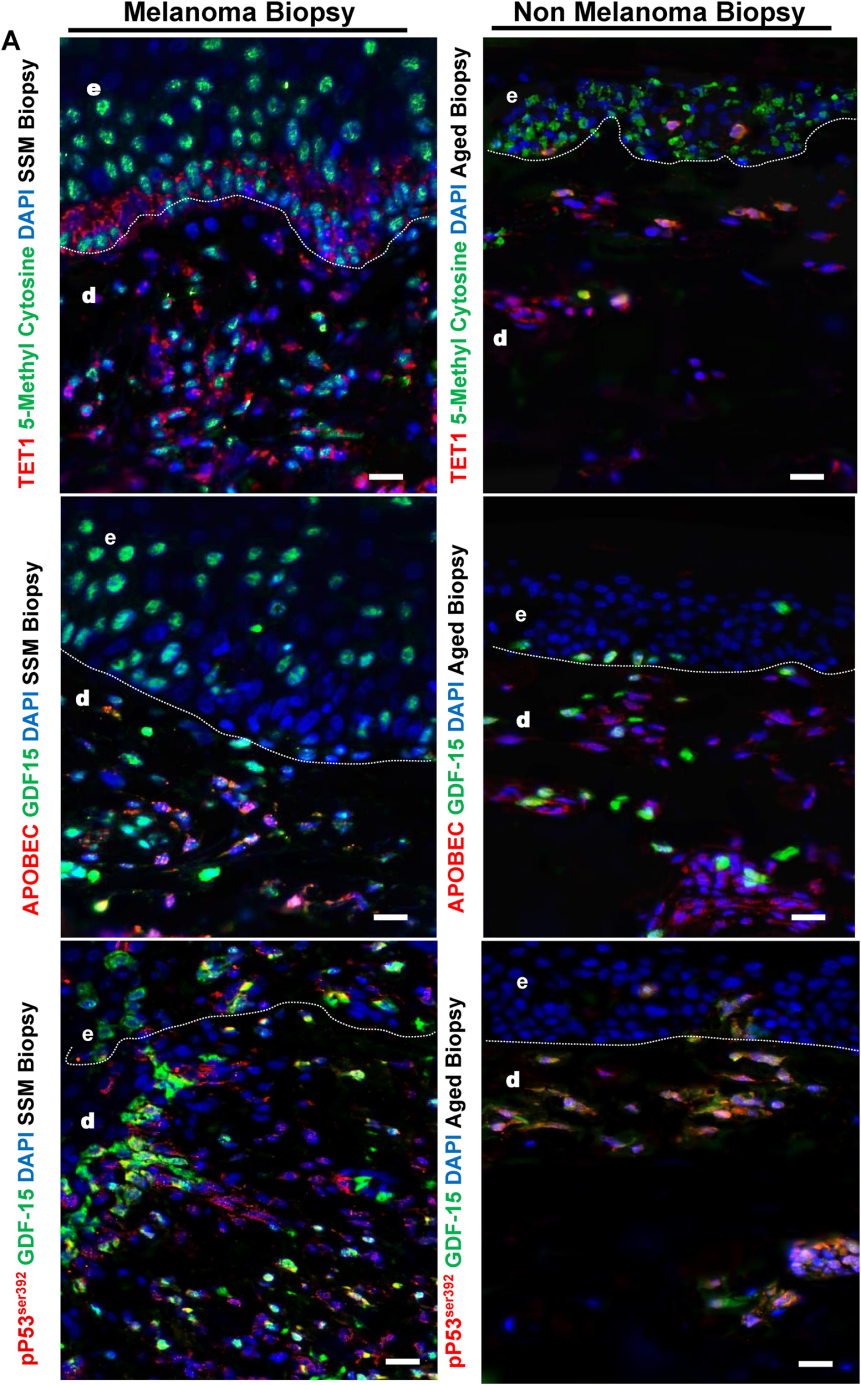
Melanoma tissue biopsy shows upregulation of DNA demethylation enzymes TET1 and APOEC in dermal compartments in melanoma versus non-melanoma controls. **A** upper panels also showed high TET1 (Red) both at basal and dermal layer in melanoma compared to aged skin biopsy levels in the dermal compartment which is a marker for 5hmc & hypomethylation but were not in the vicinity of methylated cells (Green), similarly (**A** middle panel) showed by APOBEC (Red) at dermal layer in melanoma compared to aged skin, which is a marker for hypomethylation but were not in the vicinity of methylated cells (Green). (**A** lower panel), Finally, we compared WT phospho-p53^Ser392^ Red) is expressed in GDF-15 positive cells (Green), and we observed that WT p53^Ser392^ was 50% positive for GDF-15 and 50% negative in melanoma biopsies, as compared to the healthy aged skin, where most cells were WT p53^Ser392^ positive. Finally, we sought to determine if GDF-15 is also positive for mutated phospho-P53^N329Y^.

**Fig. 10:**
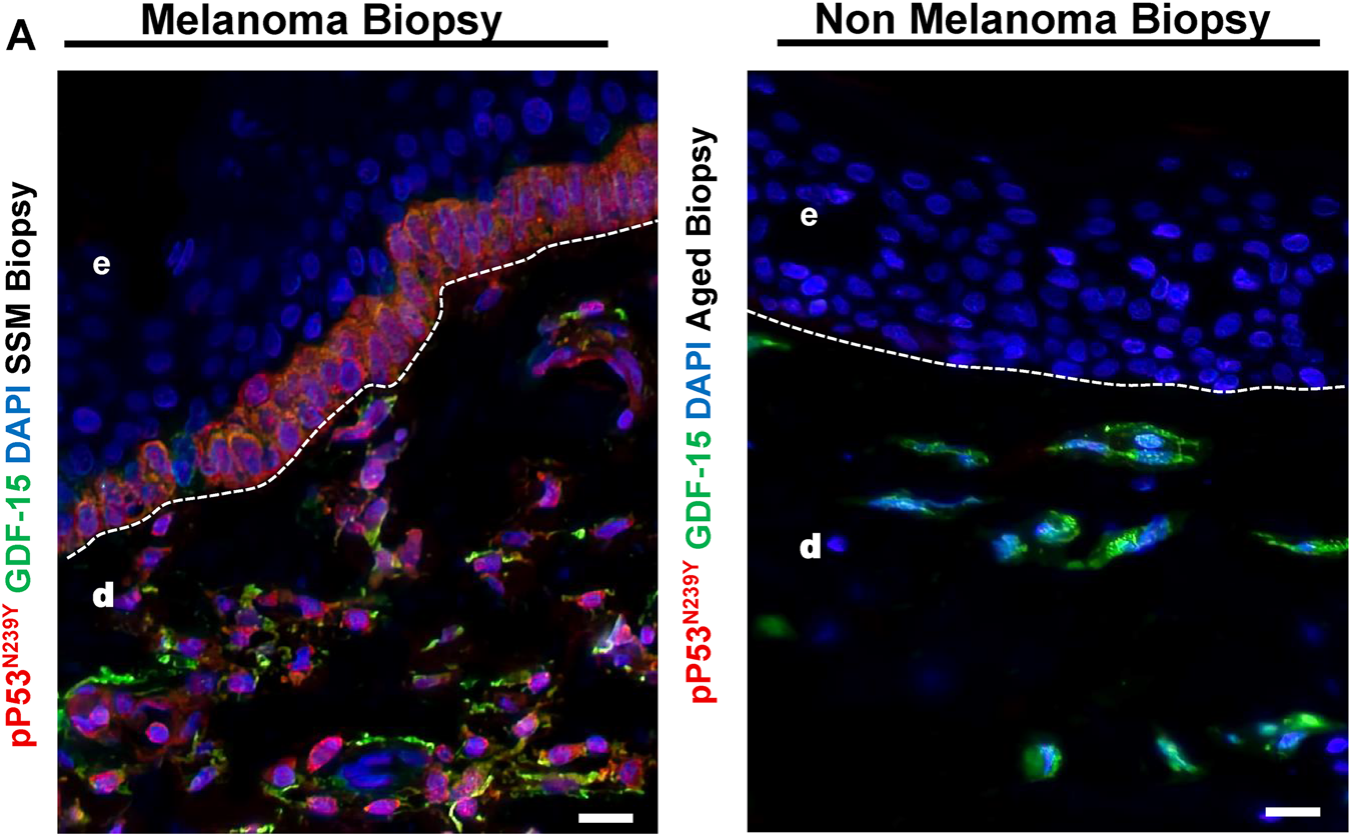
Melanoma tissue biopsy shows upregulation of mutated p53N293Y in dermal compartments in melanoma versus non-melanoma controls. **A** The immunostaining shows the basal layer is positive for Mutated P53 as well as GDF-15, including the dermal compartment; however, non-melanoma biopsies did not show any mutated Phospho-P53N239Y but some GDF-15 cells in dermis.

To determine if GDF-15 expression correlates with hyper- or hypomethylated regions in melanoma biopsies as compared to healthy aged biopsies, we performed immunostaining on tissue sections from samples of SSM patients using anti-5MC (green) and anti-GDF15 (red) antibodies. The results indicate that GDF-15 expression is much higher in melanoma biopsies as compared to the healthy aged skin, as well as is also scantily hypermethylated, as shown with low 5MC expression. (Fig. 8A. top panel) Additionally, we observed that high IL-6 expression from (Red) as compared to healthy aged skin but was not associated with high methylation levels (Green) in the dermal compartment. Although aged skin biopsy also showed high IL-6 levels in the dermal compartment, but were not in the vicinity of methylated cells (Fig. 8A, middle panels). GDF-15 expression was more prevalent in Melan A-positive metastatic melanoma cells, as shown by the co-stained yellow color, as compared to the healthy aged skin. Taken together, IL-6 induces hypomethylation in melanoma cells, which are GDF-15 and MelanA positive (Fig. 8A, lower panels). Also high TET1 (Red) both at basal and dermal layer in melanoma compared to aged skin biopsy levels in the dermal compartment which is a marker for 5hmc & hypomethylation but were not in the vicinity of methylated cells (Green) (Fig. 9A, upper panels), similarly we showed by APOBEC (Red) at dermal layer in melanoma compared to aged skin (Fig. 9B, middle panel), which is a marker for hypomethylation but were not in the vicinity of methylated cells (Green). Finally, we compared if phospho-active WT p53 in Red) is expressed in GDF-15 positive cells (in Green), and we observed that WT pP53 was 50% positive for GDF-15 and 50% negative in melanoma biopsies as compared to the healthy aged skin, where most cells were WT pP53 positive (Fig. 10A).

### Interleukin-6 regulates *GDF-15* gene promoter activity in melanoma cells

**Fig. 11:**
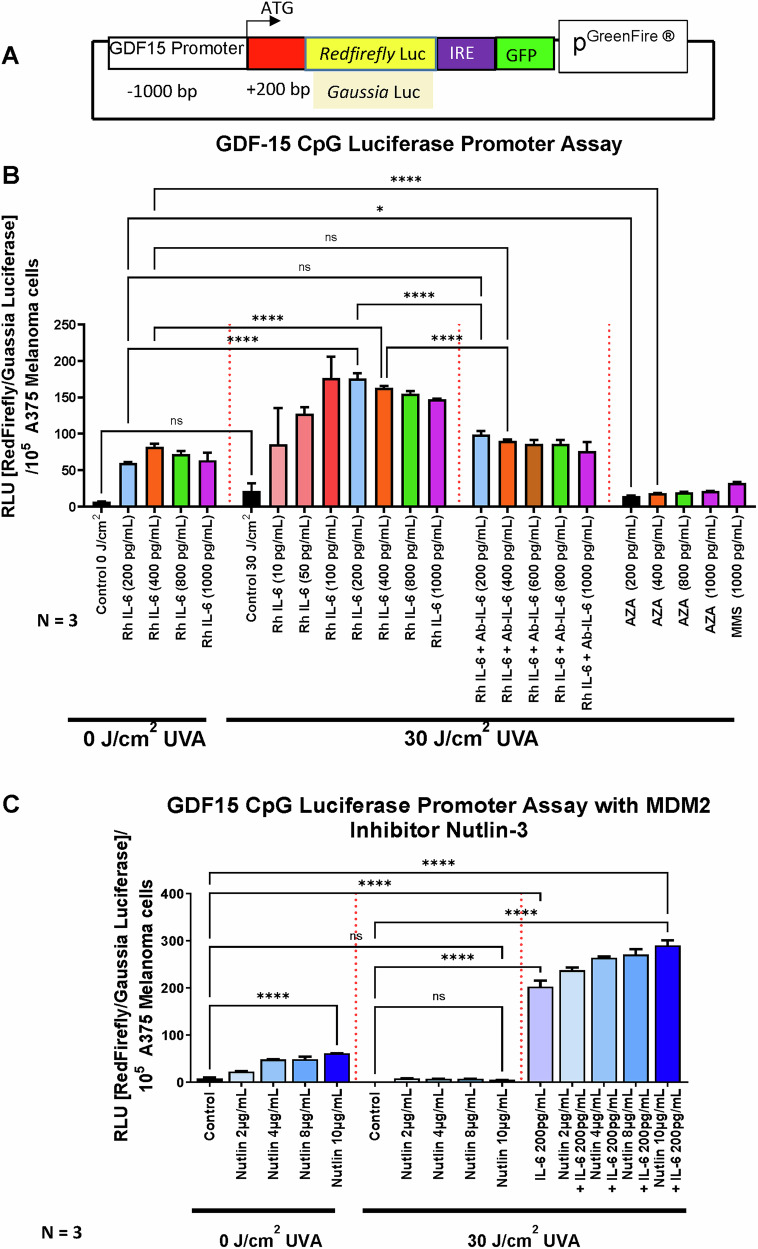
p53 transcribes GDF-15 expression via IL-6-mediated promoter hypomethylation in post-irradiated metastatic melanoma cells. **A** Luciferase assay was performed using pGreen® Red firefly-GFP/Basal Gaussia firefly on GDF-15 promoter from -1000bp in the 5’UTR to + 200 bp downstream ATG codon. The stable transfected cells were GFP sorted. Luciferase activity from red firefly was normalized with Gaussia firefly per 10^5^ A375 cells (**B**). Luciferase activity of GDF-15 5’ UTR promoter was higher in post-irradiated melanoma cells undergoing genotoxic stress supplemented with various doses of Rh IL-6 in comparison to luciferase activity from non-irradiated melanoma cells with Rh IL-6 supplementation. Additionally, post-irradiated melanoma cells supplemented with various doses of Rh IL-6 and enhanced GDF-15 promoter activity could be significantly reduced further when various doses of Anti-IL-6 antibody were added, showing the specificity of IL-6 in GDF-15 promoter activity. Global methylating and demethylating agents like MMS and AZA did not show significant promoter activity. **C** Transcription factor p53 binding at the orphan CpG promoter sites of GDF-15 was confirmed on post irradiated melanoma cells supplemented with IL-6 200 pg/mL and addition of various doses (2ug/mL to 10ug/mL) of MDM2 inhibitor Nutlin-3. This effect was not visible if the post-irradiated cells were not supplemented with IL-6, showing that the transcription factor p53 cannot act upon the hypermethylated GDF-15 promoter. Furthermore, non-irradiated melanoma cells also with supplementation of Nutlin-3 at various doses showed moderate GDF-15 promoter activity, probably due to basal IL-6 and p53 levels.

**Fig. 12:**
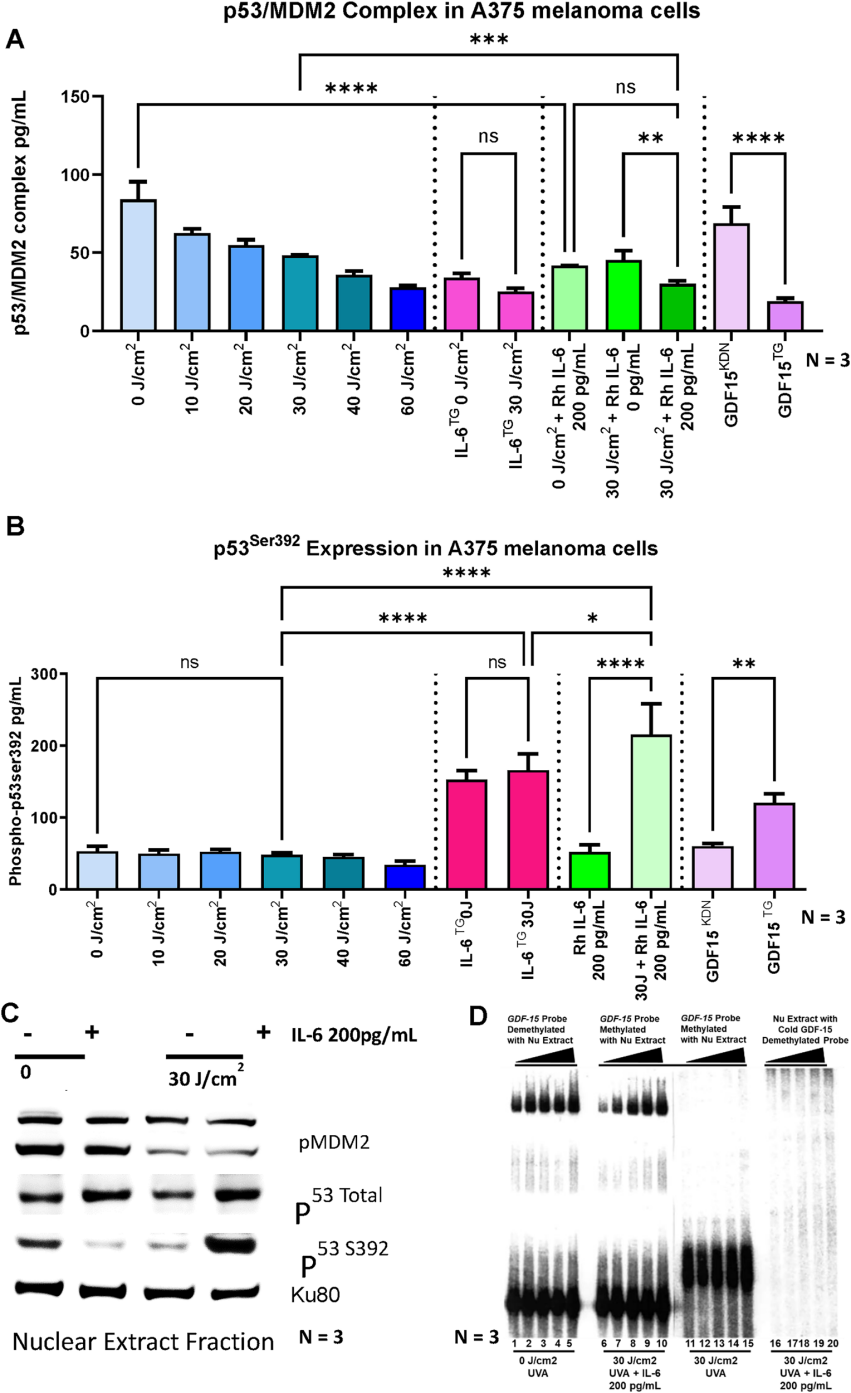
p53/MDM2 stability is reduced with IL-6 supplementation of post-irradiated melanoma cells in comparison to non-irradiated melanoma cells. **A** p53 degradation via Ubiquitination and loss of stability via complexing with MDM2 was analyzed with p53/MDM2 ELISA, where post-irradiated melanoma cells with increased UVA dose show decreased p53/MDM2 stability, whereas supplementation of IL-6 on post-irradiated melanoma showed further decrease. Non-irradiated IL-6^TG^ and irradiated IL-6^TG^ melanoma cells did not show much difference.GDF-15^KDN^ and GDF-15^TG^ served as positive and negative controls. **B** To decipher if loss of P53/MDM2 stability leads to more activated p53 we performed Phospho-p53^Ser392^ ELISA assay. Post-irradiated melanoma cells did not show high phospho-p53^Ser392^ activity, but when supplemented with IL-6, they showed significantly enhanced activated p53 levels in comparison to non-irradiated melanoma cells supplemented with IL-6. IL-6^TG^ irradiated or non-irradiated did not show any significant difference. GDF-15^TG^ and GDF-15^KDN^ served as positive and negative controls. **C** Western blotting using nuclear extracts from A375 pre- and post-irradiated melanoma cells with or without IL-6 supplementation shows increased expression of total p53 and phospho-p53Ser392 but reduced phospho-MDM2 on post-irradiated melanoma with IL-6 supplementation in comparison to post-irradiated melanoma without IL-6 supplementation; however non non-irradiated melanoma showed no difference in phospho-MDM2, reduction in total p53, and phospho-p53^Ser392^ with IL-6 supplementation. Ku80 served as a nuclear loading control. **D** EMSA assay was performed with GDF-15 methylated and non-methylated probes, showing that post-irradiated melanoma cells nuclear extracts (Nu extracts) displayed greater binding to methylated GDF-15 probes with IL-6 supplementation in comparison to without IL-6 supplementation. However, demethylated GDF-15 could bind to non-irradiated A375 Nu extracts. A cold demethylated probe served as a negative control. *P < 0.01, ***P < 0.001, ****P < 0.0001 by ANOVA multiple comparison with *Tukey’s* posthoc test.

To investigate whether IL-6, a key component of irradiated senescent fibroblasts, regulates the *GDF15* gene promoter, we stably transfected a luciferase reporter construct spanning -1000 to +40 bp of the *GDF-15* promoter into A375 melanoma cells (Fig. 11A). Increasing IL-6 concentrations enhanced the *GDF15* promoter activity of UVA exposed A375 melanoma cells in comparison to a significantly reduced *GDF15* promoter activation in A375 melanoma cells unexposed to 30 J/cm^2^ UVA irradiation. To further support our hypothesis, the addition of an IL-6 neutralizing antibody reduced *GDF15* promoter activity. The global DNA demethylating agent Azacytidine (AZA) and the methylating agent methyl methane sulfonate (MMS) did not fully restore or suppress promoter activity as effectively as Rh IL-6, suggesting that IL-6 promotes the demethylation of *GDF15* promoter CpG islands. (Fig. 11B). To explore the role of the tumor suppressor protein p53 in *GDF15* transcription, we supplemented MDM2 inhibitor Nutlin-3, in the presence and absence of IL-6 and either under non-irradiated or 30 J/cm^2^ UVA-irradiated conditions (Fig. 11C). Nutlin alone slightly increased *GDF15* promoter activity in non-irradiated melanoma cells, while showing a more significant upregulation when A375 melanoma cells where exposed to UVA followed by IL-6 200 pg/mL stimulation. This suggests a potential collaboration between p53 and IL-6 in *GDF15* transcription in A375 melanoma cells when exposed to UVA irradiation. In support of this notion, employing a p53/MDM2 complex stability assay, a significant reduction in P53/MDM2 complex formation was found in A375 melanoma cells exposed to 30 J/cm^2^ UVA irradiation and supplemented with IL-6 (Fig. 12A).

### Interleukin-6 phosphorylates p53 at position Ser392, which binds to CpG p53 sites of *GDF-15* promoter and thereby activates it in A375 melanoma cells

To investigate the role of IL-6 in activating the tumor suppressor protein p53, we analyzed all eight phospho-forms of p53 using lysates from both UVA-irradiated and non-irradiated A375 melanoma cells supplemented with Rh IL-6, *GDF-15* knockdown, and *GDF-15* overexpressing A375 melanoma cells. We performed a phospho-specific p53 antibody ELISA and normalized the results with basal p53 levels (Suppl. Figures 2C, 2D). Our findings showed that 30 J/cm^2^ UVA irradiation downregulated phospho-p53 at ser392, while Rh IL-6 supplementation upregulated it. Demethylating agents like Azacytidine also enhanced phospho-p53 ser392 levels under non-irradiated conditions. In *GDF-15* overexpressing A375 melanoma cells, we observed elevated levels of phospho-p53 ser392, whereas *GDF-15* knockdown cells exhibited reduced phospho-p53 ser392 levels, indicating that IL-6 promotes phosphorylation of p53 at ser392, facilitating *GDF-15* transcription in A375 melanoma cells (Fig. 12B). IL-6 overexpressing A375 melanoma with or without UVA irradiation did not show any significant change. We confirmed these results with lysates from A375 melanoma cells subjected to various doses of UVA irradiation, with and without Rh IL-6 supplementation, but no P53 activation was observed. The lysates of GDF-15 overexpressing A375 melanoma cells subjected to UVA-irradiation and IL-6-supplementation also did not show P53 activation. Similar results were obtained from western blotting (Fig. 12C) and melanoma biopsies (Fig. 6A), where p53 was active at ser392. To assess whether p53 can effectively bind and activate at the 5’ UTR of *GDF-15* under methylating versus demethylating conditions, we conducted an EMSA using nuclear extracts from UVA-irradiated and non-irradiated A375 melanoma cells, with or without Rh IL-6 supplementation (Fig. 12D). We found that methylated *GDF-15* probes (lanes 6-10) efficiently bound to the DNA of post-UVA irradiated melanoma cell lysate when subjected to Rh IL-6, confirming that p53 binds and activates *GDF-15*, while methylated probes (lanes 11-15) without Rh IL-6 did not bind and activate p53.

### IL-6 differentially enhances *GDF-15* transcription through WT p53^ser392^/Mutated p53^N239Y^, TET1/APOBEC activation, and subsequent phospho-STAT3 activation

**Fig. 13:**
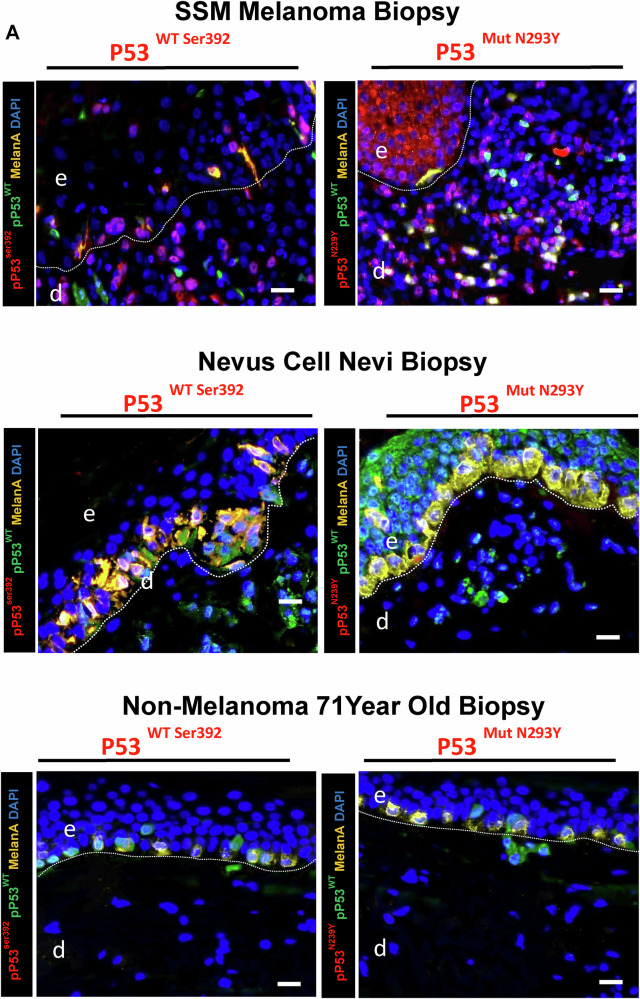
p53^Ser392^ and p53^N239Y^ show increased staining in SSM melanoma in comparison to nevi or non-melanoma controls. **A** Immunofluorescence staining using Anti-phospho-p53^Ser392^ and Anti-phospho p53N239Y antibody on Metastatic melanoma biopsies showed enhanced activated phospho-p53Ser392 (Red) and Mutated phospho-p53N239Y (Red) expression in comparison to nevus and aged healthy skin biopsies. On the contrary, the basal p53 expression was higher in nevus and aged healthy skin compared to Metastatic melanoma. We also show that enhanced Phospho-p53^N239Y^ staining clarifies the role of p53 being activated, despite apoptosis contribution remains obscure due to the loss of gene function in p53.

**Fig. 14:**
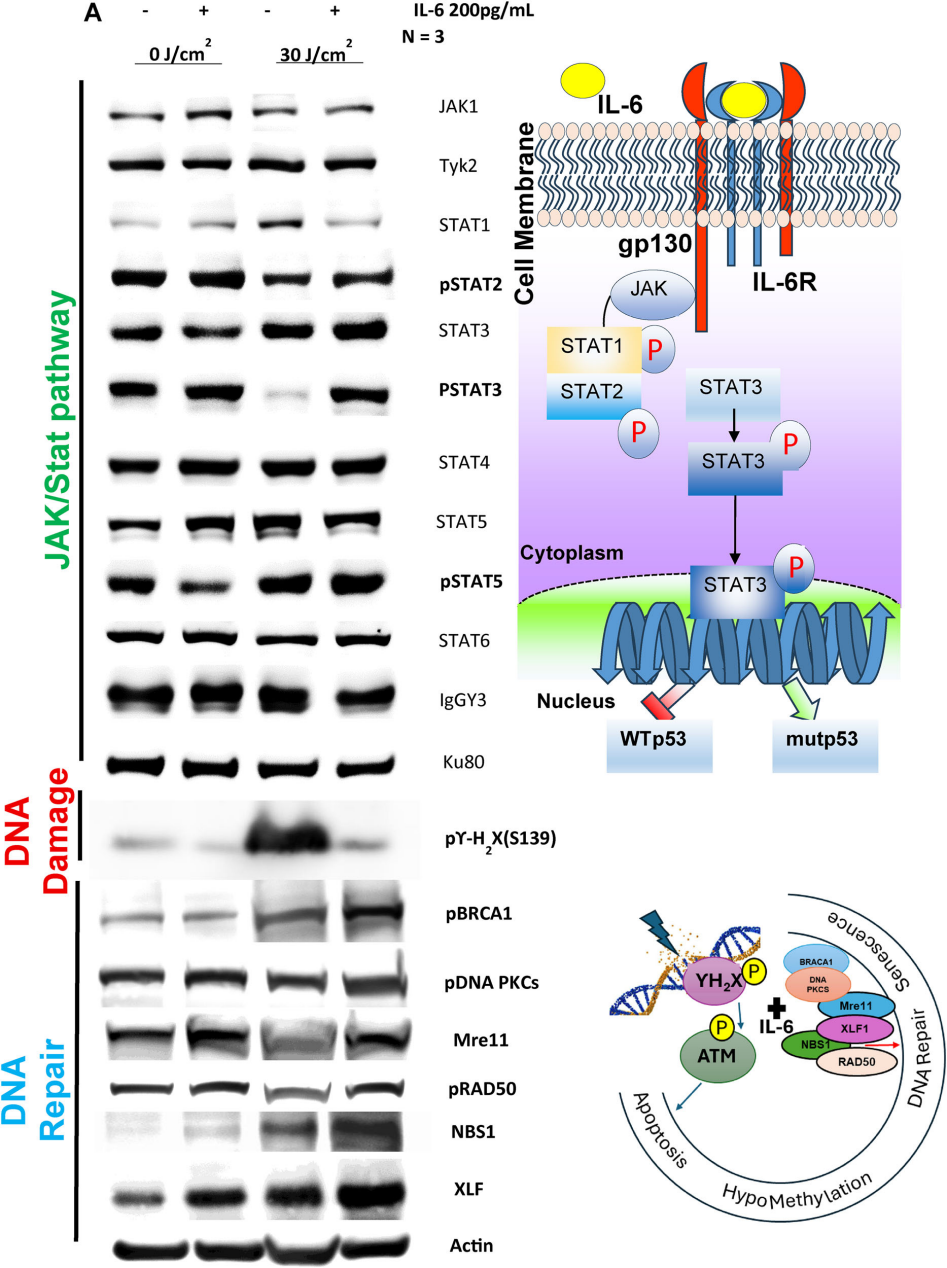
Transcription factor signaling via IL-6/STAT2-3/p53Ser392/GDF-15 and DNA damage repair BRCA/PKC/RAD50/NBS1/Mre gets upregulated with IL-6 supplementation of post-irradiated melanoma cells. **A** To find the missing link between IL-6 signaling and p53 transcription factor-mediated GDF-15 activation, western blotting with anti-STAT family proteins was explored, showing that post-irradiated A375 melanoma supplemented with IL-6 significantly upregulates phospho-STAT2/3 and phospho-STAT5 in comparison to non-IL-6 supplemented post-irradiated melanoma cells. 30 J UVA causes DNA damage using ϒ-H_2_X as a DNA damage marker, but also supplementation of IL-6 reduces that DNA damage. Furthermore, we also show that apart from DNA hypomethylation of anti-apoptosis gene GDF-15, DNA repair enzymes are also elevated with the supplementation of IL-6 on post-irradiated cells, especially RAD50, BRCA1, DNA PKCs, Mre11, NBS1, and XLF.

**Fig. 15:**
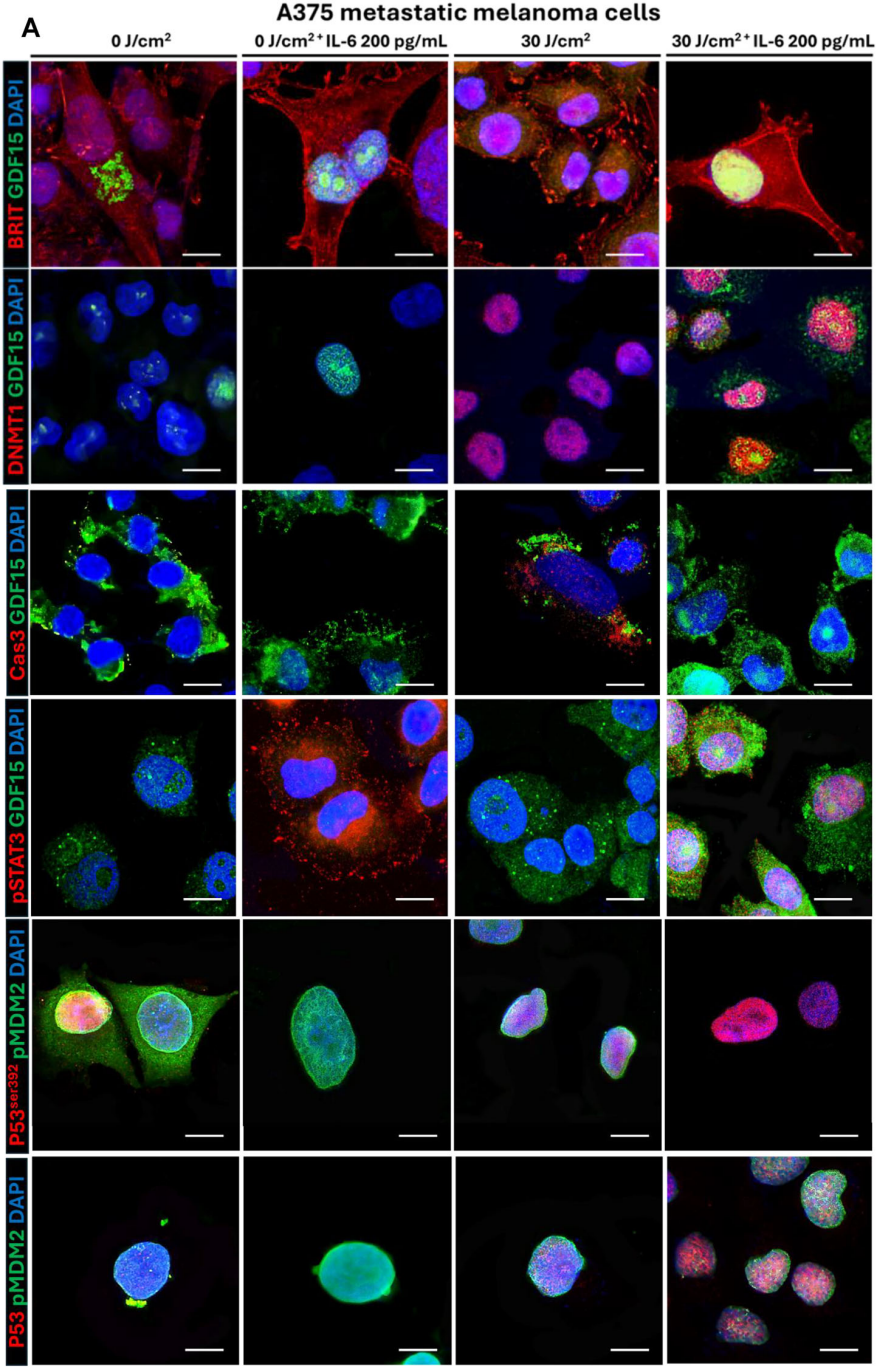
Transcription factor signaling via IL-6/STAT2-3/p53^Ser392^/GDF-15 and IL-6/APOBEC/GDF-15 axis-mediated promoter hypomethylation together might contribute toward melanoma progression. **A** Immunostaining using anti-BRIT antibody, also known as MCPH1, is a tumor suppressor gene crucial for DNA repair, cell cycle checkpoints, and preventing genome instability, shows stronger expression both in the cytosol and plasma membrane, in comparison to 30J UVA irradiation, where expression is only observed at cell foot/appendages. Immunostaining using [Anti-phosphop53^Ser392^/Red and Anti-phospho-MDM2/Green] antibody on metastatic melanoma A375 cell line showed enhanced activated phospho-p53^Ser392^ and significantly reduced phospho MDM2 expression post irradiation with IL-6 supplementation in comparison to non-irradiated cells. [Anti-GDF-15/green and Anti-DNMT1/Red] antibody on metastatic melanoma A375 cell line showed enhanced nuclear expression of GDF-15 and decreased expression of DNMT1 with post-irradiated IL-6 supplementation in comparison to non-irradiated cells. [Anti-GDF-15/green and Anti-Caspase 3/Red] antibody on metastatic melanoma A375 cell line showed enhanced GDF-15 expression and decreased caspase 3 post irradiated with IL-6 supplementation in comparison to non-irradiated cells. [Anti-GDF-15/green and phospho-Anti-STAT3^705^/Red] antibody on metastatic melanoma A375 cell line showed enhanced GDF-15 expression and phospho-Anti-STAT3^705^/Red] post irradiation with IL-6 supplementation in comparison to non-irradiated cells.

**Fig. 16:**
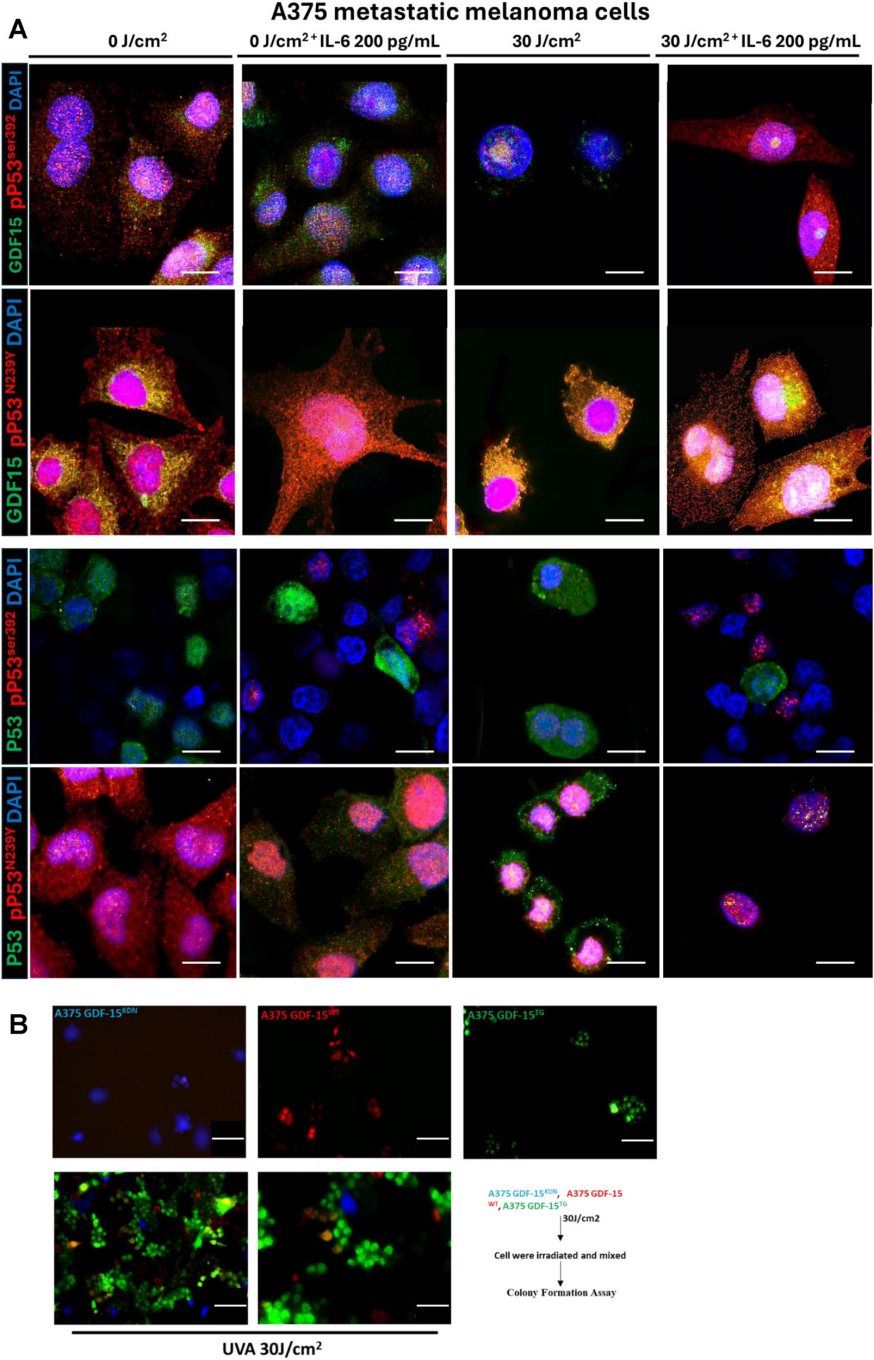
Transcription factor signaling via the mutated p53^N239Y^/GDF-15 axis could regulate GDF-15-mediated promoter hypomethylation better than p53^Ser392^, and together with an elevated GDF-15, both might contribute toward melanoma survival under stress conditions as shown by the CFU assay. **A** Immunostaining staining using mutated phospho anti-p53N239Y antibody and anti-GDF-15 (upper panel) shows that nuclear expression of the mutated p53 is most of the time present in the melanoma cells, the GDF-15 localization in the nucleus during post-UVA irradiation makes promoter hypomethylation via p53^N239Y^/GDF-15 axis better as compared to p53^Ser392^/GDF-15 axis where the p53^Ser392^ expression in the nucleus is enhanced only after post-UVA irradiation. This effect was also reconfirmed with WT p53 (lower panel). **B** Colony Formation Units (CFU) assay was carried out on post-irradiated 10^5^ A375 melanoma cells, which are either GDF-15^KDN^/blue or GDF-15^WT^/Red, or GDF-15^TG^/Green. The cells are sorted and post-irradiated and mixed further in equal proportions in Human methylcellulose with complete cytokine media (IL-6 20 ng/mL, HSC005, R&D system) shows that GDF-15^KDN^ shows poor survival as compared to GDF-15WT or GDF-15TG. IL-6 in the methylcellulose could equally hypomethylate GDF-15^WT^ and GDF-15^TG^, but high endogenous GDF-15 levels in GDF-15TG might have given early protection against UVA-induced apoptosis. Taken together, we observe that increased activated p53 along with loss of phosho-MDM2 and epigenetic unlocking of GDF-15 with reduced DNMT1 and enhanced APOBEC as shown in Suppl figure 2E. Together fathoms pro-survival in melanoma under genotoxic stress. *P < 0.01, ***P < 0.001, ****P < 0.0001 by ANOVA multiple comparison with *Tukey`s* post hoc test. −as represented by the bar denotes 200 times magnification.

Staining metastatic melanoma, Nevi and Aged healthy biopsies with an anti-phospho-p53Ser^392^ [The phosphorylation at Ser392 is also associated with the formation of p53-containing condensates involved in transcription initiation and loss of growth suppressor function] and Mutated anti-phospho-p53N239Y antibody [asparagine 239 position (N239Y), can significantly impair p53’s ability to function as a tumor suppressor] in comparison to WT Basal p53 (Green) were carried out to understand the expression of p53 in different conditions. We observe in comparison to healthy and Nevi biopsies, where WT Basal p53 (Green) was elevated, Melanoma showed elevated levels of both activated phospho-p53^Ser392^ and activated phospho-p53^N239Y,^ with the latter being higher (Fig. 13A). Metastatic melanoma, Nodular melanoma, and non-classified melanoma all showed enhanced Phospho-p53Ser392 staining, showing p53 is activated (Not shown). To explore the connection between IL-6 and p53 activation at Ser392, we examined various phosphorylated STAT transcription factors. We found that phospho-STAT2, STAT3, and STAT5 were upregulated following UVA exposure and Rh IL-6 supplementation. Our data suggests that pSTAT3 can activate both wild-type and mutated p53, suggesting that mutated p53 can also be phosphorylated by STAT3. This notion would be supported by our findings involving pPTEN, pCDKs, pmTOR, and caspase 3 being downregulated with IL-6 supplementation post UVA and rescue from apoptosis, and promotes cellular proliferation. We also observe here that IL-6 is also involved in repair of DNA Damage by upregulation DNA damage repair protein complex (BRCA1, Mre11, Rad50, NBS1, XLF) on supplementation to post UVA treated melanoma cells (Fig. 14A) Confocal Immunostaining images with anti-phospho-p53^Ser392^ and anti-phospho-MDM2^Ser166^ antibodies revealed reduced levels of phospho-MDM2 and elevated phospho-p53 Ser392 staining following UVA irradiation with Rh IL-6 supplementation (Fig. 15A). Confocal Immunostaining images with anti-phospho-p53^Ser392^ and anti-phospho-p53^N239Y^ antibodies revealed that post UVA IL-6 treatment leads to increased localization of the both phosphor-p53ser392 and mutated p53N239Y where the mutated shows increased localization (Fig. 16A). We also assessed double immunostaining of DNMT1 alongside GDF-15. We found that IL-6 supplementation and UVA irradiation decreased DNMT1 and increased GDF-15 expression in hypomethylated nuclear regions. To understand whether hypomethylation of the *GDF-15* promoter via downregulation of DNMT1 can lead to functional rescue of the melanoma cells against apoptosis, additionally, we additionally measured active caspase 3 levels and GDF-15 expression in UVA-irradiated and IL-6-supplemented melanoma cells. (Fig. 15A, Fig. 5A, Suppl. Figure 2E). IL-6 supplementation in non-irradiated melanoma cells reduced caspase 3. By contrast, IL-6 supplementation of A375 melanoma cells subjected to UVA irradiation resulted in high GDF-15 expression, but significantly reduced caspase 3 levels as opposed to high caspase 3 expression in UVA irradiated A375 melanoma cells in the absence of IL-6. We observed that STAT3 tyrosine 705 (STAT3tyr705) levels decreased after UVA irradiation, but IL-6 supplementation restored its expression (Fig. 15A). Colony Formation Units (CFU) assay was carried out on post-irradiated 10^5^ A375 melanoma cells which are either GDF-15^KDN^ (blue) or GDF-15^WT^ (red)or GDF-15^TG^ (green) Melanoma cells are sorted and after UVA irradiation, A375 melanoma cells were mixed in equal proportions in human methylcellulose with complete cytokine media. Under these conditions, GDF-15^KDN^ shows poor survival as compared to GDF-15^WT^ or GDF-15^TG^. IL-6 in the methylcellulose could equally hypomethylate GDF-15^WT^ and GDF-15^TG,^ but high endogenous GDF-15 levels in GDF-15^TG^ might have given early protection against UVA-induced apoptosis. These results collectively suggest that the anti-apoptotic gene *GDF-15* protects against damaging UVA irradiation (Fig. 16B).

## Discussion

The role of UVA irradiation in the progression of melanoma, particularly in the context of senescent fibroblasts, remains inadequately understood. A pivotal player in this dynamic is IL-6, which is markedly upregulated in senescent fibroblasts following UVA exposure when compared to their young fibroblasts. Interestingly, while IL-6 levels diminish in melanoma cells subjected to 30 J/cm^2^ UVA irradiation (a UVA dose approximately equivalent to a 15% minimal erythema dose). This decrease in IL-6 is consistent and proportional to escalating doses of UVA irradiation. Among the various genes influenced by UVA, a noteworthy candidate from the TGF-β family is growth differentiation factor 15 (GDF-15/MIC-1/NAG-1), which is elevated in the serum of metastatic melanoma patients [20] and in several other cancer types [21–23]. GDF-15 is increasingly recognized as a crucial diagnostic marker for melanoma, with its expression intricately linked to differential methylation patterns [24]. Successful knockdown of *GDF-15* has been associated with enhanced suppression of metastatic melanoma [24]. While previous research has outlined the mechanistic pathways involving GDF-15 through the AKT/PI3K/PTEN signaling axis in melanoma, the specific contributions of UVA and the tumor microenvironment to melanoma progression have remained largely unexplored. In this study, we elucidate the mechanism by which IL-6 activates GDF-15 through hypomethylation of orphan promoters, mediated by the IL-6/STAT3/p53 signaling axis. Although the role of IL-6 in promoting melanoma migration around cancer-associated fibroblasts (CAFs) is already established [25], we observed that irradiated melanoma cells exhibit heightened migration under genotoxic stress when exposed to conditioned media (CM) from UVA-irradiated senescent and young fibroblasts, despite a reduction in IL-6 [26] and IL-6R expression within the melanoma cells. Conversely, GDF-15 secretion was significantly higher in melanoma cells than in senescent or young fibroblasts following UVA irradiation. Notably, CM from UVA-exposed senescent fibroblast cells appeared to rescue post-UVA-exposed melanoma cells from apoptosis. Indeed, the *GDF-15* orphan CpG promoter was found to be hypomethylated, contrasting with the global DNA hypermethylation induced by IL-6 [27]. Furthermore, IL-6 overexpression in young fibroblasts yielded the highest survival rates for melanoma cells post-UVA, alongside a myriad of pleiotropic effects that influenced cell cycle regulation, energy homeostasis, and anti-apoptotic pathways. Previous studies have also demonstrated that IL-6 induces a reduction in cyclin-dependent kinases (CDKs) in primary melanoma [28] and enhances apoptosis in TNF-α resistant melanoma cells [29]. However, in our case, in metastatic melanoma WM-266-4, the cells are not resistant to TNF-alpha, but were always found to be hypermethylated, suggesting reduced transcription regardless of UVA exposure, and IL-6 prevented cellular apoptosis. For the first time, we identified that IL-6 induces hypomethylation of the *GDF-15* promoter through enhanced expression of cytidine deaminases like apolipoprotein B mRNA editing catalytic polypeptide-like (APOBECs [30]) and Thymine-DNA Glycolases (TDGs [31]), coupled with a decrease in DNA methyltransferase 1 DNMT1 expression. Importantly, the transcription of *GDF-15* was found to be executed by phospho-p53 at Ser392 or phospho-p53^N239Y^ via the IL-6/STAT1/2/3 axis. Previous reports indicate that UVB exposure enhances p53 phosphorylation at Ser392 and promotes apoptosis in melanoma, particularly with p53Ser392 knockdown in SKH1 hairless mice [32] and p53 transcribes *GDF-15* [33]. We also found that IL-6 binds to and hypomethylates the *GDF-15* promoter at p53 CpG sites following UVA treatment, which can be repressed using Nutlin-3, which prevents stabilization of p53 via disrupting the interaction between p53 and MDM2. A375 melanoma has wild-type p53 but has been reported as inactive in previous studies, limiting its role in apoptosis due to factors like increased MDM2/X [34] expression, instability of the MDM2/p53 complex, and downregulated p53 or USF1 [35], leading to pro-tumorigenic effects [36]. In fact, mutation of p53 at N239Y was found in the nucleus of melanoma biopsies and melanoma cells. We did not observe a decrease in active p53 or p53-mediated inhibition of the G1 cell cycle phase via P21 or reduced CDKs. Additionally, metastatic melanoma biopsies showed more enhanced *GDF-15* hypomethylation, with elevated p53Ser392 levels correlating with increased colony-forming units (CFUs) in post-UVA-treated melanoma cells. Taken together, we report that UVA-induced IL-6 promotes *GDF-15* gene promoter hypomethylation while activating p53Ser392 or p53N239Y via the STAT3 axis, eventually enhancing melanoma cell survival under genotoxic stress of UVA irradiation. Treatment of patients with GDF-15 inhibitor Ponsegromab (Phase II Clinical trial) [37–39] and IL-6 inhibitor Tocilizumab [40–43] might show a better prognosis likely by promoting apoptosis in cancer and melanoma patients. Although once diagnosed with melanoma, no melanoma patients are exposed to UVA, nevertheless, keeping the p53 mutated cells transcription at bay, the combined chemotherapy treatment may hold promise for treatment of melanoma patients thereby reducing chances of melanoma remission.

## Methods and materials

### Melanoma cell lines

Melanoma cell lines [wild-type (A375 MM, WM-115, WM-266-4) and genetically modified (A375 IL-6^TG^, A375 GDF-15^KDN^, GDF-15^TG^) were used. Melanoma cell lines were grown in DMEM media with 10% FCS supplemented (2 mM L-glutamine and 100 IU of penicillin-streptomycin/mL of DMEM.

### Transwell migration assay

Migration of A375 melanoma cells was assessed by the Transwell chamber technique (Corning GmbH, Germany) with slight adaptations to an earlier described technique [44]. The lower compartment of the chamber was loaded with 600 µl of conditioned medium derived from supernatants with or without 30 J/cm^2^ UVA irradiated old and young dermal fibroblasts or recombinant chemokines diluted to concentrations of 1 nM, 10 nM, and 30 nM in serum-free medium (Ultra CULTURE medium, Lonza Cologne, Germany). The upper chambers were loaded with or without 30 J/cm^2^ UVA irradiated 10^5^ A375 melanoma cells/well in a serum-free medium. Following a 6 hr incubation period at 37 °C, 5% CO_2_, perforated filters were removed, the upper chamber and the polycarbonate membrane were cleaned with cotton ear buds, and the membrane was further fixed and stained with DiffQuick® Stain (Medion Diagnostics AG, Switzerland). The migrated cell count was analyzed from three technical replicates in 7 different high-power fields (20X). The experiment was independently repeated three times. The statistical significance of the migration index in different groups was calculated using ANOVA multiple comparisons with Tukey´s posthoc test. P-values less than 0.0001 were considered significant.

### Antibody arrays and ELISA

To identify SASP factors released by senescent fibroblasts upon UVA irradiation, which may promote tumor progression, young and senescent fibroblasts were irradiated with 30 J/cm^2^ of UVA and incubated for 12 hours to generate the conditioned medium (CM) which was first normalized to the cell number and thereof were subjected to Ray-Biotech Human Cytokine G series 4000 antibody arrays for cytokines, chemokines and their corresponding receptors. The array was blocked with 100 µl blocking buffer for 30 minutes at RT with gentle shaking. 70ul of samples were added to each well with 1ul of internal control and further incubated at RT with gentle shaking for 2 hours. The well was washed 2 times, 10 minutes each with wash buffer I and 5 minutes each with II. Add 70µul of Biotinylated antibody (prepared by adding 300µul of blocking buffer) to each well and incubate overnight at 4 °C. The primary antibody was decanted and washed 3 times, 2 minutes each, with wash buffer I and II, and the Streptavidin-tagged IR-dye 800 secondary antibody was diluted 1:5000 and incubated 70 µl in each well. Further incubated for 2 hours in the dark and thereafter washed twice for 10 minutes each with buffers I and II, rinsed with distilled water. The arrays were centrifuged at 1000 rpm for 3 minutes and dried at RT and imaged by the LICOR imaging device. In parallel, normalized CM was analyzed on an ELISA kit (R & D Systems) according to the manufacturer’s instructions.

### Annexin V-Apoptosis assay

The apoptosis was measured in the wildtype and transgenic A375 MM cells using Annexin V antibody coupled to APC against inner membrane phospholipid phosphatidylserine (PS) of the apoptotic cells which flips outside in apoptotic cells, (BD Pharmingen, Cat# 550474. The melanoma cells of different genotypes were co-stained with Sytox Blue dye, Thermoscientific, Cat# S34857) which is permeable in dead cells, were regarded as early apoptosis when Annexin-V was only positive, late apoptosis when positive for both Sytox Blue and were regarded as dead when only Sytox positive. A375 melanoma cells were exposed to either with or without UVA and either with or without CM derived from senescent HDF and further incubated for 12 hours. The cells were washed with cold PBS, and 10^5^ cells were resuspended in 100 µl of 1x binding buffer (BD Pharmingen, 556454). 5 µl of Annexin V and 1 µl of SytoxBlue/SytoxRed were added and incubated at room temperature in the dark for 15 minutes. Cells were washed 2 times with 1x binding buffer and resuspended in 400 µl of 1x binding buffer to be analyzed by flow cytometry.

### Immunofluorescence and immunochemistry

Immunostaining of cryosections and paraffin sections obtained either from the skin from the skin of healthy individuals, as well as from melanomas (TissueArray.Com LLC, USA, #Malignant melanoma and healthy skin, ME1004h, ME482a) was performed using a previously described protocol (Wang et al., 2006). Paraffin sections of melanomas were incubated with antibodies against [IL-6 #12912], [C-Cas3#9664], [C-Parp#94885], [pMDM2#3521], [p53#2527], [p53Ser392# 65390], [5-MC#MABE1081, Millipore], [pSTAT3#9145], [DNMT1#5032], [Actin#4967], [GDF-15#79996] were purchased from Cell signal Isotype IgG served as negative control, and Alexa Fluor 488 and Alexa Fluor 555 (Molecular Probes) served as secondary antibodies. Immunohistochemistry of melanoma sections were incubated with secondary goat ([Anti-Rabbit-Biotin; BA-1000] and [Anti-Mouse-Biotin; BA-2000]; Vector Laboratories) antibodies and further developed with ([ABC-AP; AK-5003] and [ABC-Elite; PK-6103]; Vector Laboratories) and substrates ([Vector Blue Alkaline Peroxidase; SK-5300], [ImmPACT DAB HRP; SK-4105] and [ImmPACT NovaRed HRP; SK-4805] Vector Laboratories). Photomicrographs were taken using a Zeiss Axiophot microscope and AxioVison 4.8 software (Zeiss).

### In vivo tumor assay

Male SCID mice 8 weeks of age were injected subcutaneously in the flank with 10^6^ A375 melanoma cells either alone or in combination with replicative senescent (REP SEN, old) and non-senescent (NON-SEN; young overexpressing IL-6) fibroblasts in a total volume of 200 µl. Tumor volumes were calculated with caliper measurements performed weekly to monitor and track tumor growth. Five mice of the following genotypes were assessed. (*n* = 5 per group): 1) A375 irradiated 30 J/cm^2^; 2) A375MM irradiated 30 J/cm^2^ + (REP SEN)/ replicative senescent FF95 fibroblasts at CPD 68); 3) A375MM non-irradiated*;* 4) A375 MM irradiated 30 J/cm^2^ + (NON-SEN)/young fibroblasts overexpressing IL-6^TG.^ All animal studies were conducted according to the institutional guidelines conforming to international standards under the approval of the Ethical Committee of the NCSR “Demokritos” (41/24–06–2021).

### Western blotting

Cells were stimulated with chemokines for 1 h, harvested, and lysed with 1x RIPA containing protease and phosphatase inhibitors (Roche) for a 20 min incubation period on ice. Lysates were centrifuged at 13.000 rpm for 30 min. Protein quantification was performed by BCA assay (Pierce). Lysates were loaded with 4x LDS sample buffer (Nupage) and subjected to electrophoresis in a 12% Bis-Tris polyacrylamide gel (Novex, Life Technologies). Antibodies such as [IL-6 #12912], [GP-130 #3732], [pATM #5883], [pCHK2 #2661], [pATR #30632], [CHK1#2G1D5], [Cyclin E1 #20808], [Cds25A #3652], [p21 #2947], [p27 #3686], [pAKT #4060], [mTOR #2972], [PTEN #9188], [pmTOR#5536], [pPTEN#9551], [pRB#8516], [B-Catenin#8480], [C-Cas3#9664], [C-Parp#94885], [AIF#4642], [BAX#5023], [CoxIV #4850], [pATF-4#11815], [pMDM2#3521], [p53#2527], [p53Ser392 #65390], [p53Ser6#9285], [p53Ser9#9288], [p53Ser15#9286], [5-MC #MABE1081,Millipore], [p53Ser20#9287], [p53Ser33#2526], [p53Ser37#9289], [p53Ser46#2521], [JAK1#3344], [Tyk2#14193], [STAT1#7649], [pSTAT24441], [STAT3#12640], [pSTAT3#9145], [STAT4#2653], [STAT5#25656], [pSTAT5#4322], [STAT6#9362], [IGGY3#3788], [Ku80#2753], [TDG#99105], [AID#4949], [APOBEC#41494], [TET#18950], [DNMT1#5032], [Actin#4967], [GDF-15#79996], [pϒ-H_2_X#2577], [pBRCA1#9009], [DNA PKCs#4602], [pRAD 50#99215], [Mre11#4895], [NBS1#14956], [XLF#2854] were purchased from Cell signal, [Mutated P53 N235K,N239Y, #orb11211] was procured from Biorbyt. Antibodies were diluted 1:2000 in 3% SKMT and incubated overnight at 4 °C. Visualization was performed by enhanced chemiluminescence (ECL kit, Amersham Biosciences).

### EpiTect Methyl II PCR assay

Epitect Methyl II Melanoma PCR array was procured from Qiagen (# EAHS-8080Z, 96-well) and was screened with cells from melanocytes, primary melanoma, and metastatic melanoma incubated with or without the conditioned medium from old and young fibroblast cells exposed to 30 J/cm^2^ UVA irradiation or left unirradiated. The isolation of genomic DNA and restriction enzyme treatment were carried out according to the manufacturer´s protocol. The percentage methylation was determined using the formula % Hypermethylation F_HM_ = Cmd/(Cmo-Cmsd), % Hypomethylation F_UM_ was calculated using the formula % F_UM_ = Cms/(Cmo-Cmsd), and intermediate methylation was calculated using F_IM_ = 1- F_HM_-F_UM_.

### MELP (MspI Enrichment Linker PCR) methylation assay

The CpG methylation site within the promoter regions of the GDF-15 was analyzed using MethPrimer^25,^ and primers were designed using Methyl Primer Express® software (sequences are in Supplementary Table 2). Instead of using classical HpaII sites, we used MspI sites, which were more specific to internal cytosine methylation of CpG islands and less frequent in the total number of sites in the promoter region than HpaII. The methylation percentage was calculated using the formula % Hypermethylation FHM = Cmd/(Cmo-Cmsd), % Hypomethylation FUM was calculated using the formula % FUM = Cms/(Cmo-Cmsd), and intermediate methylation was calculated using FIM = 1- FHM-FUM. Where Cmo is the delta Ct value obtained from a mock sample without enzyme, Cmd is the delta Ct value obtained from methylation-insensitive enzyme (Msp1), Cms is the delta Ct value obtained from the methylation-sensitive enzyme (SmaI/XmaI), and Cmsd is the delta Ct value obtained from methylation-sensitive and insensitive enzymes (MspI+SmaI) or (MspI+xmaI).

### Luciferase Reporter Assay

The luciferase assay was carried out by cloning promoter -1000bp to +200 bp *GDF-15* gene upstream of luciferase in pGreenFire® (pGF1-mCMV, System Biosciences, TR010PA-P) and Control (pGF1-CMV, System Biosciences, # TR011PA-1). The A375 cells were transduced, and positive cells were selected with GFP fluorescence sort. The assay was carried out using (Promega Firefly Luciferase assay kit, #16186. The 10^5^ A375 melanoma cells were irradiated with or without UVA 30 J, as well as in the presence or absence of RhIL-6 of varying doses, and subjected to luciferase estimation as per the manufacturer’s protocol.

### P53/MDM2 complex assay

P53/MDM2 assay complex was determined by sandwich ELISA using Immunoset® p53/MDM2 complex assay kit from ENZO Life Science International, ADI-960-070, as per the manufacturer’s protocol. Briefly, 10 µg of nuclear extract in 100 µl was obtained from wild-type A375 cells, which were UVA irradiated or left unirradiated, and RhIL-6 supplementation, as well as from Transgenic A375 GDF-15^KDN^, GDF-15^TG^ cells. The nuclear extracts were incubated with stably coated dried p53 antibody, 1:250 diluted in coating buffer for 60 minutes at room temperature. The plates were washed 4 times with 400 µl of washing buffer and further incubated with 100 µl of 1:250 diluted MDM2 antibody for 1 hour at room temperature. Wash 4 times with 400 µl and further incubated with 100 µl of 1X Streptavidin-HRP conjugate antibody for 30 minutes, washing was repeated, and 100 µl of TMB was pipetted into each well and incubated for 30 minutes in the dark at room temperature. The reaction was stopped with 100 μL of 1 N HCl, and reading was taken at 450 nM. Recombinant human P53/MDM2 Complex standards were prepared using reconstituted 4 parts of p53 (1.2 µg/mL) into one part of MDM2 (0.32 µg/mL) and thereafter incubating for 1 hour and plated using 2-fold serial dilution.

### DNA binding assay (EMSA)

Electromobility assay (EMSA) was carried out using 1ul of 100pMol of Biotinylated probes of 20–25 bp length. Complementary probes, both unmethylated and methylated at 5 cytosines (The methylated cytosine GFD15 probes from Thermoscientific GmbH, Germany), were annealed using Annealing buffer (New England Biolabs, B7002S) in 20 µl volume following a standard PCR protocol (95 5 min 1 cycle, drop of −1.5 °C/cycles for 50 cycles, 4 °C infinite). EMSA kit from (Panomics AY1000) was used as per the manufacturer´s instruction with minor changes (the binding complex consisted of 6ul of nuclear extract, 1 µl of Poly d(I-C) 1 ug/ul, 2 ul of 5x Binding buffer, 1 µl of biotinylated ligated TF probe) and was further run in a 10% TBE gel using 0.5% TBE buffer at 80 V. The gel was transferred to Amersham-Nylon+ (GE Healthcare, RPN119B) using 0.5% TBE buffer at 300 mA for 1 hour. The membrane was shortly dried on a paper towel and crossed-linked by Stratagene automatic UV crosslinker for 3 minutes and blocked for 15 minutes. 8 µl of 1ug/ml of streptavidin-linked HRP was added in the blocking buffer and the membrane was incubated for 1 hour and was furthered washed in buffer and developed using 1x of (20x LumiGLO, Cell signal,7003) chemiluminescence. The data was analyzed using Fusion software (Vilber Lurmat GmbH).

### Anchorage-independent growth assay

The anchorage-independent growth assay was performed according to the manufacturer´s instructions (CellBio Labs Inc.). Briefly, 10^5^ melanoma cells or melanocytes in DMEM without FCS were mixed with 1.2% top agar and 2X DMEM without FCS in a 1:1:1 ratio. 75 µl of this mixture/well was placed in each well of a 96-well flat-bottom plate, resulting in 10^4^ cells/well. The plate was incubated for 15 min at 4 °C, and thereafter cells containing soft agar were overlaid with 100 µl of serum-free medium or CM from old or young fibroblasts. The plates were incubated at 37°C, in an atmosphere of 5% CO_2,_ for 7 days and were analyzed using a Zeiss inverted microscope. The total number of colonies was counted after dissolving the agar and incubation with agar with SYBR green. The fluorescence intensity as a measure of colony formation was determined at 485/520 nanometers.

### Me-Dot blot Assay

Bisulfite-converted DNA was taken from A375 cells treated either with RhIL-6 at different concentrations or from A375 cells incubated with UVA irradiated senescent human dermal fibroblast. Up to 2 µg bisulfite converted DNA was denatured with 10x volume of (0.4 M NaOH, 10 mM EDTA) for 15 minutes. The DNA was further blotted on the Zeta-Probe membrane (BioRad, # 9201419), which was pretreated with distilled water for 5 minutes. The blotted membrane was washed with 0.4 M NaOH and neutralized with 2x SSC buffer, dried, and UV cross-linked (Automatic mode). The cross-linked membrane was hybridized for 12 hours in hybridization buffer (5x SSPE, 0.5% SDS, 5x Denhardt’s solution) with 4nMol 3’-Biotinylated-TEG sequence probes at Tm-5 °C (where the putative methylated Cytosine of the CpG seq was replaced with deoxyUracil) against the GDF-15 gene at p53 promoter binding sites. The DNA-oligoProbe was dot blotted and further washed 3 times, 5 minutes each, with (10X SSPE and 0.5% SDS) buffer. The membrane was further incubated with Anti-Biotin HRP conjugate (1:2000) (Cell Signal, #7075S) for 120 minutes and washed for 15 minutes with 1x TBST and further developed with 20x LumiGLO (Cell Signal, #7003) chemiluminescence.

### Demethylation assay

DNA demethylation was determined by inverse proportional calorimetric quantification of 5-methyl-cytosine binding using a DNA demethylase Activity/Inhibition assay kit (Epigentek Groups Inc, P-3008) as per the manufacturer’s procedure. Briefly, 10 µg of nuclear extract were obtained from Wildtype A375 cells with or without UVA and RhIL-6 treatment, as well as from Transgenic A375 cells. The nuclear extracts were incubated with stably coated methylated DNA constructs in 1X Demethylase assay buffer for 90 minutes at 37°C. The wells were washed 3 times with 150 µl of wash buffer and incubated with 50 µl of 5-methyl-cytosine capture antibodies for 1 hour at room temperature, further washed with 150 µl of wash buffer, and incubated for 30 minutes with detection antibody. The plates were washed four times with 150 µl of wash buffer. Further 50 µl of enhancer, 100 µl of developer solution, and 100 µl of stop solution were sequentially added and read at 455 nM with a reference of 655 nM The Demethylase activity and Inhibition were calculated as follows.

$${\mathrm{Demethylase}}\,{\mathrm{activity}}({\mathrm{OD}}/{\rm{h}}/{\mathrm{mg}})=\frac{[{\mathrm{OD}}\,({\mathrm{control}}-{\mathrm{blank}})-{\mathrm{OD}}({\mathrm{sample}}-{\mathrm{blank}})]}{[{\mathrm{Protein}}\,{\mathrm{Amount}}({\upmu} {\rm{g}})/1000] \times \,{\mathrm{Hour}}}$$

$${\mathrm{Inhibition}}\, \% =\frac{(1-[{\mathrm{OD}}({\mathrm{control}}-{\mathrm{blank}})-{\mathrm{OD}}({\mathrm{no}}\,{\mathrm{inhibitor}}\,{\mathrm{sample}}-{\mathrm{blank}})]\times 100 \% ]}{[{\mathrm{OD}}({\mathrm{control}}-{\mathrm{blank}})-{\mathrm{OD}}({\mathrm{inhibitor}}\,{\mathrm{sample}}-{\mathrm{blank}})]}$$

DNA demethylase activity is calculated using the formula:

$$\begin{array}{lll}{\rm{Activity}} ({\rm{ng}}/{\rm{h}}/{\rm{mg}})&=&[{\rm{OD}} ({\rm{control}}-{\rm{blank}})-{\rm{OD}}({\rm{sample}}-{\rm{blank}})]\\&&\left.{\rm{Slope}} \times {\rm{protein}}\, {\rm{Amount}} (\upmu {\rm{g}})/1000\right] \times {\rm{ Hour}} \end{array}$$

## Supplementary information

Supplementary Figures

Western Blots as supplementary files

## Data Availability

Original data is available upon request. The full-length, uncropped original western blots are shown in the ‘Supplementary Material’.
