# Supplementary material for: The dark side of the light (UVA): melanoma microenvironment and cell survival strategies
Source: Cell Death Discov. 2025 Oct 20;11:466. doi: 10.1038/s41420-025-02751-y (PMC12537921; doi:10.1038/s41420-025-02751-y)
Supplement: Supplementary file 1 — Supplementary Figures [file 41420_2025_2751_MOESM1_ESM.docx]

**Supplementary Figure 1.**

| Name | Ratio UVA Senescence/  Young | | Ratio Non-irradiated Senescence/  Young | Fold  Change | P Value |
| --- | --- | --- | --- | --- | --- |
| Decorin | | 5,4 | 7,16 | 0,76 | 0,013855 |
| IL-6 | | 3,5 | 1,14 | 3,12 | 0,01466 |
| GRO | | 3,5 | 2,31 | 1,55 | 8,24E-05 |
| IL-8 | | 3,0 | 1,96 | 1,56 | 0,002792 |
| MMP-1 | | 2,3 | 2,88 | 0,81 | 0,006735 |
| MCP-1 | | 1,7 | 0,98 | 1,73 | 0,175439 |
| GDF-15 | | 1,1 | 2,20 | 0.50 | 0,001166 |
| MCP-3 | | 1,5 | 2,50 | 0,62 | 0,066321 |
| DPPIV/CD26 | | 2,1 | 1,39 | 1,54 | 0,00035 |
| MMP-10 | | 1,8 | 1,00 | 1,89 | 1,75E-05 |
| IL-2Ra | | 1,7 | 1,65 | 1,04 | 0,460907 |
| IGF-BP-6 | | 1,7 | 1,72 | 1,001 | 0,843818 |
| ANGPTL4 | | 1,7 | 1,10 | 1,54 | 0,001972 |
| Beta 2 M | | 1,6 | 0,99 | 1,64 | 0,002705 |
| ErbB2 | | 1,6 | 1,63 | 0,98 | 0,792633 |
| Cathepsin S | | 1,5 | 1,27 | 1,23 | 2,23E-06 |
| CD23 | | 1,5 | 1,19 | 1,28 | 0,005407 |
| EpCAM | | 1,5 | 1,72 | 0,87 | 0,216611 |

**(A)** Antibody array depicts the list of differentially regulated genes expression with conditioned medium (CM) form young and old senescence fibroblast either UVA irradiated or non-irradiated.

**(B)**

**UVA irradiation of A375 MM cells leads to downregulation of IL-6**

**FF95 CPD 60**


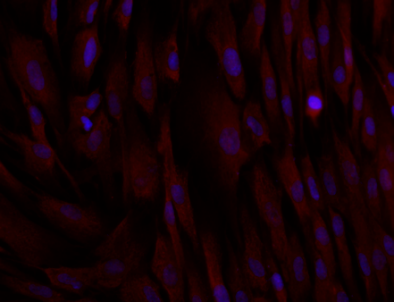

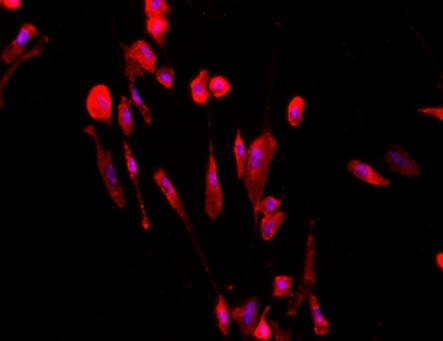


**40X**

**40X**

**IL-6**

**0 J**

**IL-6**

**30 J**


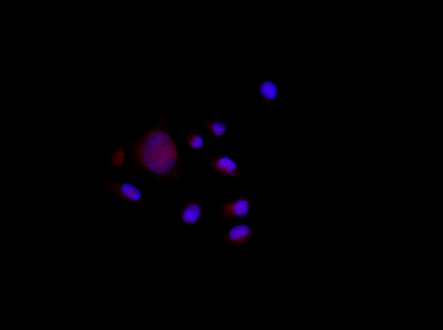

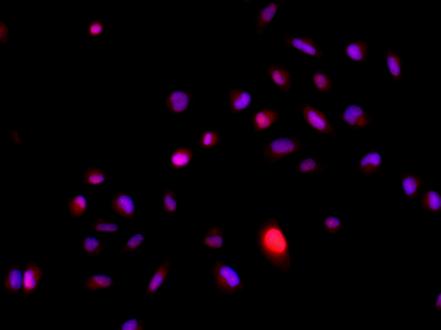


**40X**

**40X**

**GDF-15**

**0 J**

**GDF-15**

**30 J**


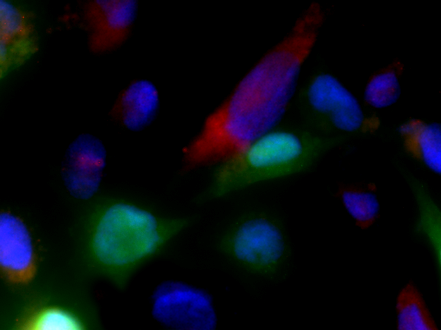

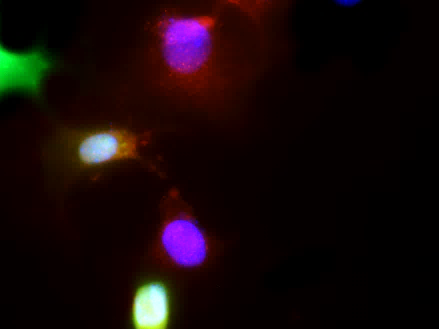


**IL6-DDK**

**IL6-DDK**

**100X**

**100X**

**0 J**

**30 J**

**A375**

**FF95 CPD 60**


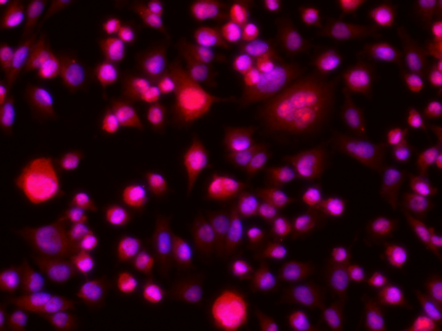

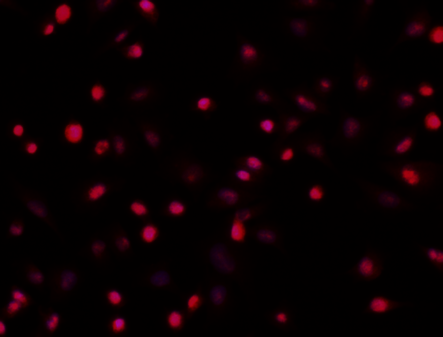


**IL-6**

**A375**

**IL-6**

**40X**

**0 J**

**30 J**

**40X**

**A375**

**FF95 CPD 60**

*

**(C) UVA irradiation of A375 MM cells leads to downregulation of IL-6 and IL-6R**


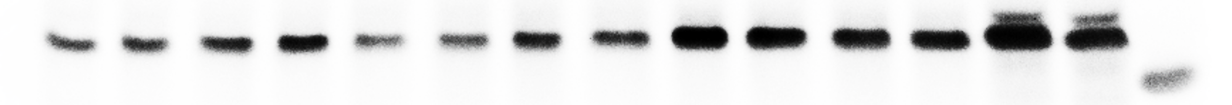

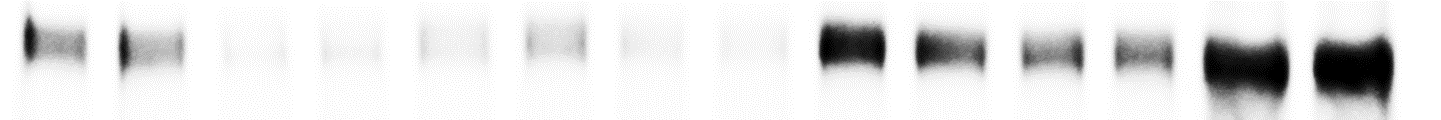


**IL-6**

**GP130**

**30J/cm^2^**

**+ RhIL-6**

**30J/cm^2^**

**0J/cm^2^**

**A375 MM cells**

**β-Actin**


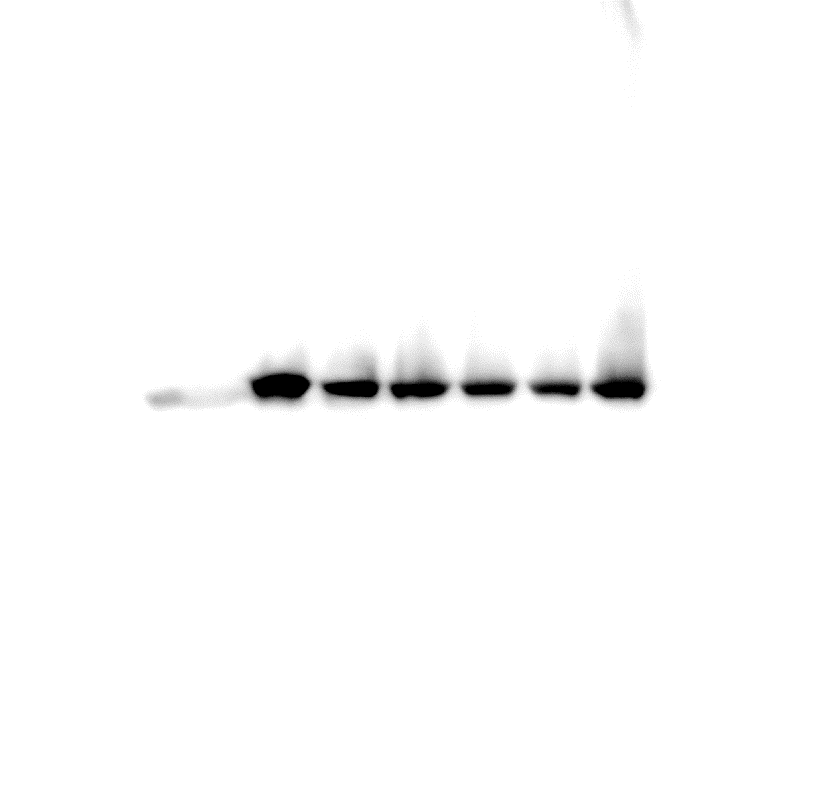


**Southern western blot showing methylation of melanoma cells as detected with anti-5-methylation cytosine antibody**


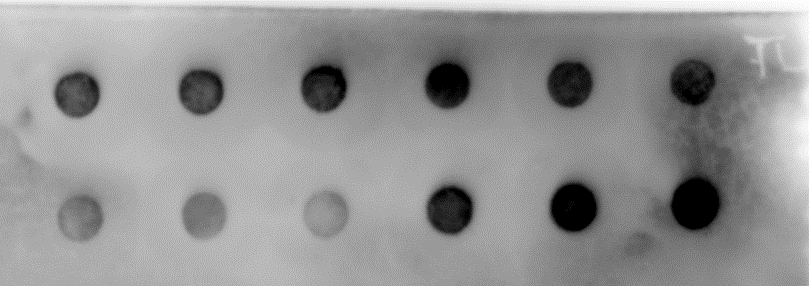

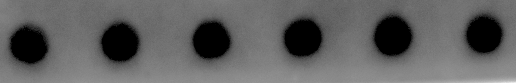


**Anti-5methylcytosine**

**Ethidium bromide staining**

Control A375

UVA 30J/cm2

5-Azacytidine

IL-6 100 pg/mL

**A375 MM**


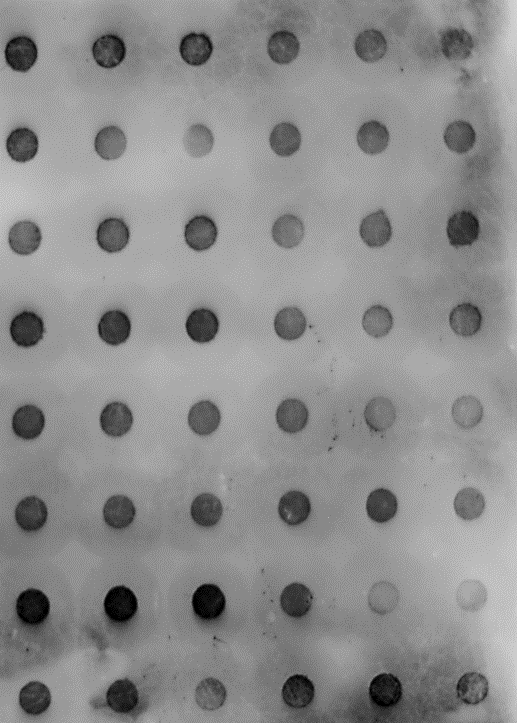


**WM-266-4 MM**

**SK-Mel2 MM**

**BLM MM**

WM-266-4 Control

UVA 30J/cm2

5-Azacytidine

IL-6 100ng/mL

SK-Mel2 Control

UVA 30J/cm2

5-Azacytidine

IL-6 100ng/mL

BLM Control

UVA 30J/cm2

5-Azacytidine

IL-6 100ng/mL

**Anti-5methylcytosine**

Control

Control

Control

UVA 30J/cm2

UVA 30J/cm2

UVA 30J/cm2

5-Azacytidine

5-Azacytidine

5-Azacytidine

IL-6 100 pg/mL

IL-6 100 pg/mL

IL-6 100 pg/mL

**(D)**

**(E)**

**Meth-Dot blot showing GDF-15 methylation of melanoma cells as detected with GDF-15 promoter specific methylation hybridization probes**.

**(F)**

**GDF-15 MethProbe**

**A375 MM**

**GDF-15 MethProbe**

**WM-266-4**


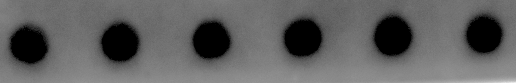


**Anti-Biotin HRP**

**IL-6** **10 ng/µL**

**UVA**  **0J/cm^2^**

**IL-6** **10 pg/mL**

**IL-6** **100 pg/mL**

**IL-6** **200 pg/mL**

**IL-6** **0 pg/mL**

**UVA 30J/cm^2^**

**Ethidium Bromide**

0 ng/ml to 200 pg/mL IL-6 with UVA and 5 mc antibody

0 ng/ml to 200 pg/mL of IL-6 w/o UVA and 5MC antibody


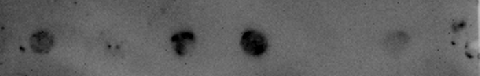

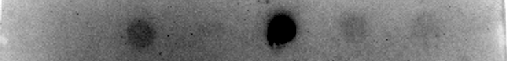


**Supplementary Table 1**

F(HM) = Δct Md/(Δct Mo- Δct Msd)

F(UM) = Δct Ms/(Δct Mo- Δct Msd)

F(IM) = 1-F(HM)-F(UM)

AAAAAA = Gene Specific Primer


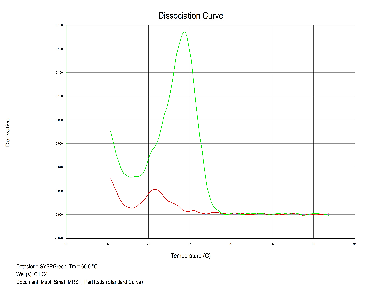

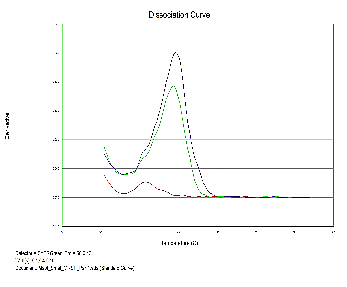

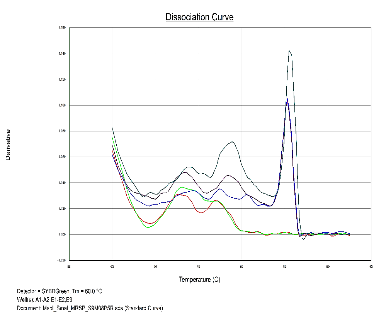

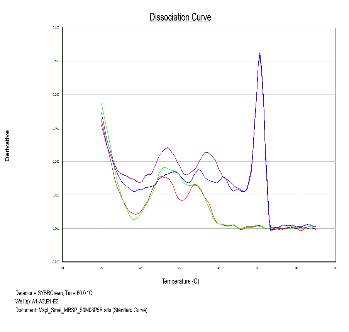


30J/cm^2^

0J/cm^2^

SmaI


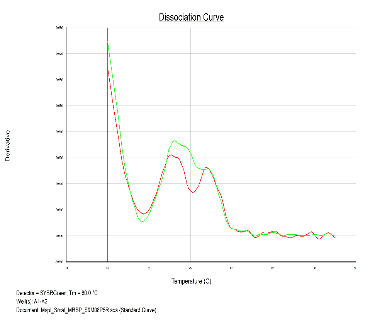


No Enzymes

30J/cm^2^

0J/cm^2^

SmaI

MspI

30J/cm^2^

0J/cm^2^

30J/cm^2^  + IL-6 200ng

30J/cm^2^

0J/cm^2^

30J/cm^2^  + IL-6 200ng

Tm = 75-80^◦^C

CCCGGGAAAAAA

SmaI

(FP)

GGG

Tm = 60^◦^C

SmaI

MspI

30J/cm^2^

0J/cm^2^

| **Symbol** | **Melanocyte 30 J/cm^2^ UVA** | | **WM-115**  **CM YNG 0 J/cm^2^ Fibroblast** | | **WM-115**  **CM YNG 30 J/cm^2^ Fibroblast** | | **WM-115**  **CM OLD**  **0 J/cm^2^ Fibroblast** | | **WM-115**  **CM OLD**  **30 J/cm^2^ Fibroblast** | | **WM-266-4**  **CM OLD 0 J/cm^2^ Fibroblast** | |
| --- | --- | --- | --- | --- | --- | --- | --- | --- | --- | --- | --- | --- |
|  | **HM%** | **UM%** | **HM%** | **UM%** | **HM%** | **UM%** | **HM%** | **UM%** | **HM%** | **UM%** | **HM%** | **UM%** |
| **ADAMTS18** | **10,04** | **51,02** | **0,06** | **99,4** | **1,52** | **98,48** | **2,01** | **97,99** | **2,55** | **97,45** | **2,22** | **30,92** |
| **AKAP12** | **3,67** | **96,33** | **0,09** | **99,91** | **0,39** | **99,61** | **0,84** | **99,16** | **0,68** | **99,32** | **3,82** | **98,18** |
| **CDH3** | **7,81** | **92,19** | **0,56** | **99,44** | **0,47** | **99,53** | **0,67** | **99,33** | **0,07** | **99,93** | **1,52** | **98,48** |
| **CDH8** | **8,06** | **91,94** | **1,04** | **98,96** | **2,06** | **97,94** | **1,57** | **98,49** | **2,85** | **97,15** | **8,86** | **91,14** |
| **DNAJC15** | **5,53** | **94,47** | **35,57** | **39,65** | **48,06** | **40,72** | **40,2** | **24,78** | **48,87** | **39,57** | **24,06** | **47,21** |
| **DPP4** | **8,20** | **91,8** | **0,46** | **22,53** | **0,63** | **24,67** | **0,45** | **33,93** | **1,32** | **30,41** | **2,12** | **15,45** |
| **ENC1** | **3,43** | **96,57** | **0,58** | **99,42** | **1** | **99** | **0,77** | **29,26** | **1,99** | **98,01** | **10,75** | **89,25** |
| **GDF15** | **86,19** | **13,8** | **11,59** | **10,85** | **18,61** | **9,28** | **8,77** | **10,4** | **8,88** | **20,18** | **9,12** | **90,88** |
| **IRF18** | **2,40** | **97,6** | **0,09** | **99,91** | **0,27** | **43,48** | **0,3** | **45,03** | **0,44** | **38,98** | **1,65** | **32,6** |
| **LRP2** | **10,49** | **89,51** | **1,52** | **98,48** | **2,45** | **97,55** | **1,81** | **98,19** | **3,93** | **96,07** | **12,7** | **87,3** |
| **LRRC2** | **5,60** | **94,4** | **0,04** | **99,96** | **0,46** | **99,54** | **0,02** | **99,98** | **0,51** | **99,49** | **1,56** | **98,44** |
| **MME** | **13,98** | **86,02** | **4,71** | **95,29** | **9,42** | **90,58** | **7,35** | **92,65** | **19,44** | **80,56** | **20,1** | **27,26** |
| **PPP1R3C** | **10,04** | **89,96** | **2,99** | **19,45** | **6,3** | **14,61** | **6,58** | **4,3** | **15,99** | **84,01** | **6,35** | **2,72** |
| **PPP2R4** | **2,95** | **97,05** | **0,16** | **99,84** | **0,15** | **99,85** | **0,18** | **99,82** | **0,41** | **99,59** | **0,93** | **99,07** |
| **PRDX2** | **10,99** | **89,01** | **0,74** | **16,35** | **0,32** | **18,43** | **0,87** | **18,83** | **1,61** | **17,61** | **1,05** | **42,74** |
| **QPCT** | **19,42** | **80,58** | **50,42** | **0,17** | **45,1** | **0,31** | **48,07** | **0,15** | **99,69** | **1,31** | **1,79** | **98,21** |
| **RASEF** | **5,79** | **94,21** | **3,54** | **3,29** | **4,7** | **3,41** | **3,08** | **1,63** | **4,32** | **32,59** | **23,33** | **14,54** |
| **RB1** | **2,31** | **97,69** | **0,01** | **99,99** | **0** | **100** | **0** | **100** | **0,01** | **99,99** | **0,01** | **99,99** |
| **SOCS2** | **10,95** | **89,05** | **0,5** | **99,5** | **1,66** | **98,45** | **1,61** | **98,39** | **2,57** | **97,43** | **3,35** | **47,41** |
| **SOCS3** | **0,45** | **99,55** | **0,63** | **99,37** | **0,07** | **99,93** | **0,29** | **99,71** | **0,3** | **99,7** | **1,01** | **98,99** |
| **THBD** | **27,96** | **72,04** | **6,53** | **93,47** | **11,37** | **88,63** | **11,84** | **88,61** | **16,71** | **83,29** | **27,86** | **31,23** |
| **TNFRSF10A** | **4,89** | **95,11** | **29,06** | **2,82** | **28,68** | **1,59** | **38,33** | **0,99** | **37,01** | **0,99** | **0,74** | **25,78** |
| **TP53INP1** | **1,32** | **98,68** | **99,64** | **0,36** | **99,16** | **0,84** | **99,85** | **0,15** | **98,48** | **1,52** | **10,72** | **89,28** |
| **TPM1** | **1,40** | **98,6** | **0,95** | **99,05** | **1,2** | **98,8** | **1,21** | **98,79** | **1,56** | **98,44** | **7,37** | **92,63** |
| **Color Code** | **0-6%** |  | **6-20%** |  | **>21%** |  | **>41%** |  | **>61** |  | **>81** |  |
| **ADAMTS18** | ADAM mettalopeptidase with thrombospondin type 1 motif, 18 | | | | | | | | | | | |
| **AKAP12** | A kinase (PRKA) anchor protein 12 | | | | | | | | | | | |
| **CDH3** | Cadherin 3, type 1, P-Cadherin (placental) | | | | | | | | | | | |
| **CDH8** | Cadherin 8, type 2 | | | | | | | | | | | |
| **DNAJC15** | DnaJ (Hsp40) homolog, subfamily C, member 15 | | | | | | | | | | | |
| **DPP4** | Dipeptidyl-peptidase 4 | | | | | | | | | | | |
| **ENC1** | Ecotodermal-neural cortex (with BTB-like domain) | | | | | | | | | | | |
| **GDF15** | Growth and differentiation factor 15 | | | | | | | | | | | |
| **IRF18** | Interferon regulatory factor 8 | | | | | | | | | | | |
| **LRP2** | Low density Lipoprotein related protein 2 | | | | | | | | | | | |
| **LRRC2** | Leucine rich repeat containing 2 | | | | | | | | | | | |
| **MME** | Membrane mettalo-endopeptidase | | | | | | | | | | | |
| **PPP1R3C** | Protein phosphatase 1, regulatory (inhibitory) subunit 3C | | | | | | | | | | | |
| **PPP2R4** | Protein phosphatase 2A activator, regulator subunit 4 | | | | | | | | | | | |
| **PRDX2** | Peroxiredoxin 2 | | | | | | | | | | | |
| **QPCT** | Glutaminyl-peptide cyclotransferase | | | | | | | | | | | |
| **RASEF** | RAS and EF-hand domain containing | | | | | | | | | | | |
| **RB1** | Retinoblastoma 1 | | | | | | | | | | | |
| **SOCS2** | Suppressor of cytokine signaling 2 | | | | | | | | | | | |
| **SOCS3** | Suppressor of cytokine signaling 3 | | | | | | | | | | | |
| **THBD** | Thrombomodulin | | | | | | | | | | | |
| **TNFRSF10A** | Tumor necrosis factor receptor superfamily, member 10a | | | | | | | | | | | |
| **TP53INP1** | Tumor protein p53 inducible nuclear protein | | | | | | | | | | | |
| **TPM1** | Tropomyosin 1 (alpha) | | | | | | | | | | | |

**Legend to Supplementary figure 1.**

The supplementary figure 1 shows the **(1A)** Antibody array with the list of differentially regulated genes expressed in the conditioned medium (CM) form young and old senescence fibroblast either UVA irradiated or non-irradiated, with fold change and P-values. The experiment was repeated individually 3 times and normalized with GAPDH expression levels. The statistical significance was calculated using Students T-TEST with * = p<0.05, **= p<0.005, *** =p <0.0005. **(1B)** Immunostaining micrographs show the downregulation of IL-6 expression in 0 J/cm^2^ and 30J/cm^2^ UVA irradiated A375 melanoma cells (upper panel left) and 0 J/cm^2^ and 30J/cm^2^ UVA irradiated Fibroblast cells (upper panel right). Immunostaining micrographs show the downregulation of GDF-15 expression in 30J/cm^2^ UVA irradiated A375 melanoma cells in comparison to control 0 J/cm^2^ (Lower panel left). Immunostaining micrographs of fibroblast cells expressing IL-6-DDK Tagged using anti-DDK antibody (Red) and A375 melanoma cells stained with FITC green at 0 J/cm^2^ and after 30J/cm2 (Lower panel right). Note that IL-6 expressed by fibroblast (Anti-DDK ab) are taken up by A375 melanoma cells. **(1C)** UVA irradiation leads to downregulation of IL-6 and IL-6R in A375 melanoma cells. **(1D, 1E)** Southwestern blotting using DNA from different melanoma cells where the cells were either irradiated with 30J/cm^2^ UVA or non-irradiated or treated with 100 ng/mL of Rh IL-6 or 100 ng of Azacytidine. All the groups were later blotted with 5-methyl-cytosine Antibody. We observe Rh IL-6 at 100 ng/mL does a global hypermethylation of the melanoma DNA (1**F**) Using the Meth-dot blot, A375 and WM-266-4 melanoma cells were either non irradiated or irradiated and then treated with different doses of recombinant human IL-6 from 0 ng/mL to 200 ng/mL. The cell DNA was isolated and probed with methylated GDF-15 promoter sequence coupled to Biotin. Secondary Antibody against Biotin coupled to HRP was further used to hybridize the development of the membrane using chemiluminescence. Although Rh IL-6 at 100 ng/mL does a global hypermethylation of the melanoma DNA but increasing IL-6 ng/mL leads to hypomethylation of GDF-15 promoter DNA.

**Legend to Supplementary Table 1.**

The supplementary Table 1 shows melanoma cells co-cultured with CM from 30 J/cm² UVA-exposed human dermal senescent fibroblasts (HDF) which were analyzed using the Epitect® methyl PCR array, exhibited alterations in the methylation states of CpG islands across melanocytes and primary and metastatic melanoma cell lines. These included hypermethylation (FHM), intermediate methylation (FIM), and hypomethylation (FUM). Notably, UVA-irradiated CM from senescent fibroblasts resulted in altered hypomethylation levels in the primary melanoma cell line WM-115. Specifically, the anti-apoptotic gene GDF-15 showed 9% hypomethylation when incubated with CM from young, irradiated fibroblasts and 20% hypomethylation when co-cultured with CM from irradiated senescent fibroblasts. In contrast, the metastatic cell line WM-266-4 demonstrated 90% hypomethylation of GDF-15 (Fig. 3B). For comparison, melanocytes served as controls, showing 86% hypermethylation for GDF-15. Taken together CM from Senescence fibroblast alters post irradiated melanoma

**Supplementary Figure 2.**

**(A) Graphical representation of methylation sensitive restriction PCR(MSRP) of *GDF-15***

**Gene promoter and MRSP.**


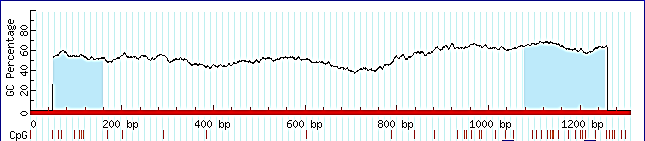


ATG

+4kb

-47 kb

-920 kb

No Enzymes

**SmaI**

**MspI**

**MspI + SmaI**

Bisulphite conversion

Methyl restriction specific PCR amplification of DNA with Methylated and Non-methylated primers and in combination.


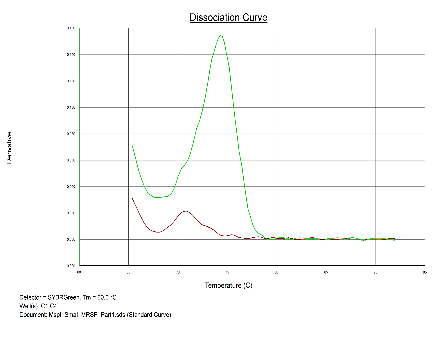

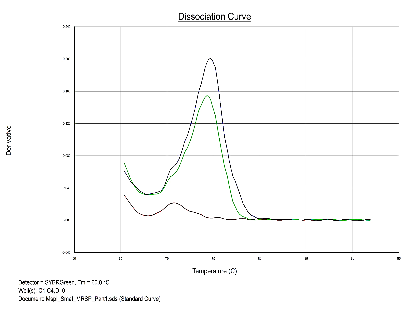

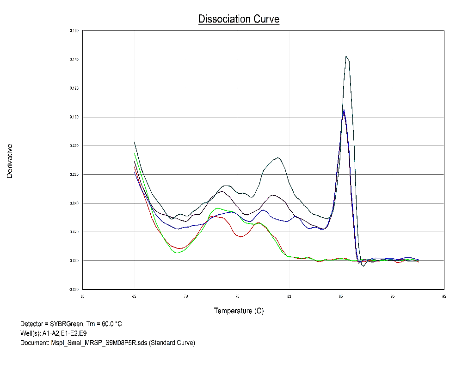

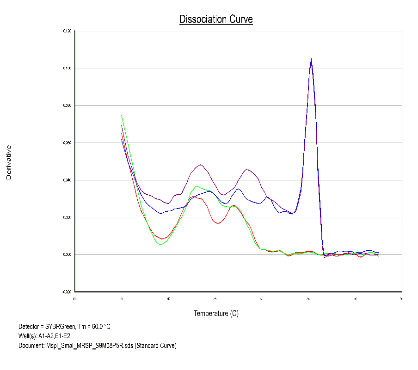


0J/cm^2^

30J/cm^2^


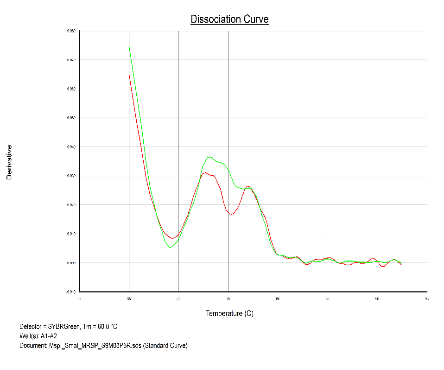


**No Enzymes**

0J/cm^2^

30J/cm^2^

**SmaI + MspI**

0J/cm^2^

30J/cm^2^

30J/cm^2^  + IL-6 200pg

0J/cm^2^

30J/cm^2^

30J/cm^2^  + IL-6 200pg

(FP)

**MspI**

0J/cm^2^

30J/cm^2^

**SmaI**

**(B)**

**SmaI**

F(HM) = Δct Md/(Δct Mo- Δct Msd)

F(UM) = Δct Ms/(Δct Mo- Δct Msd)

F(IM) = 1-F(HM)-F(UM)

xxxxxxxx = Gene Specific Primer

Tm = 75-80^◦^C

SmaI

(FP)

CCCGGGCAAGAA

(FP)

**SmaI is methylation sensitive, MspI is methylation insensitive restriction enzyme. Md = methylation dependent. Mo = Mock digest, Msd = Methylation dependent and sensitivity. HM = hypermethylated UM = Unmethylated.**

**(C)**

GAGCAG**GCGGAGACGGACAAAGT*CCGG***GGACTATAAAGGCCGGTCCGGCAGCATCTGGTC

(F Primer1) **MspI/HpaII**

AGTCC**CAGCTCAGAGCCGCAA*C***CTGCACAGCC***TCTAGA***ATG**CCCGG*G***CAAGAACTCAGGACGGTGAATGGCTCTC

(F Primer2) XbaI MspI/SmaI

CGGAGATGCTCCTGGTGTTGCTGGTGCTCTCGTGGCTGCCGCATGGGGGCGCCCTGTCTCTGGCCGAGGCGAGCCGCGCAAGTTT***CCCGGG***ACCCTCAGAGTTGCACTCCGAAGACTCCA**GATTCCGAGAGTTGCGGAAA**CGCTACGAGG

MspI/SmaI (R Primer2)

ACCTGCTAACCAGGCTGCGGGCCAACCAGAGCTGGGAAGATTCGAACACCGACCTCGTCCCGGCCCCTGCAGTCCGACCGACACCGGGGTGTCGCTCCAGACCTATGATGACTTGTTAGCCAAAGACTGCCACTGCATA***ACTAGT***TGAGCAGTCCTGGTCCTTCCACTGTGCACCTGCGCGGAGGACGCGACCTCAGTTGTCCTGCCCTGTGGAATGGGCTCAAGGTTCCTGAGACACCCGATTCCTGCCCAAACAGCTGTATTTATATAAGTCTGTTATTTATTATTAATTTATTGGGGTGACCTTCTTGGGGACTCGGGGGCTGGTCTGATGGAACTGTGTATTTATTTAAAACTCTGGTGATAAAAATAAAGCTGTCTGAACTGTT***GGATCC***

BamHI

**(D) Homology representation of pyro sequence of Bisulphite converted *GDF-15***

**Gene promoter.**


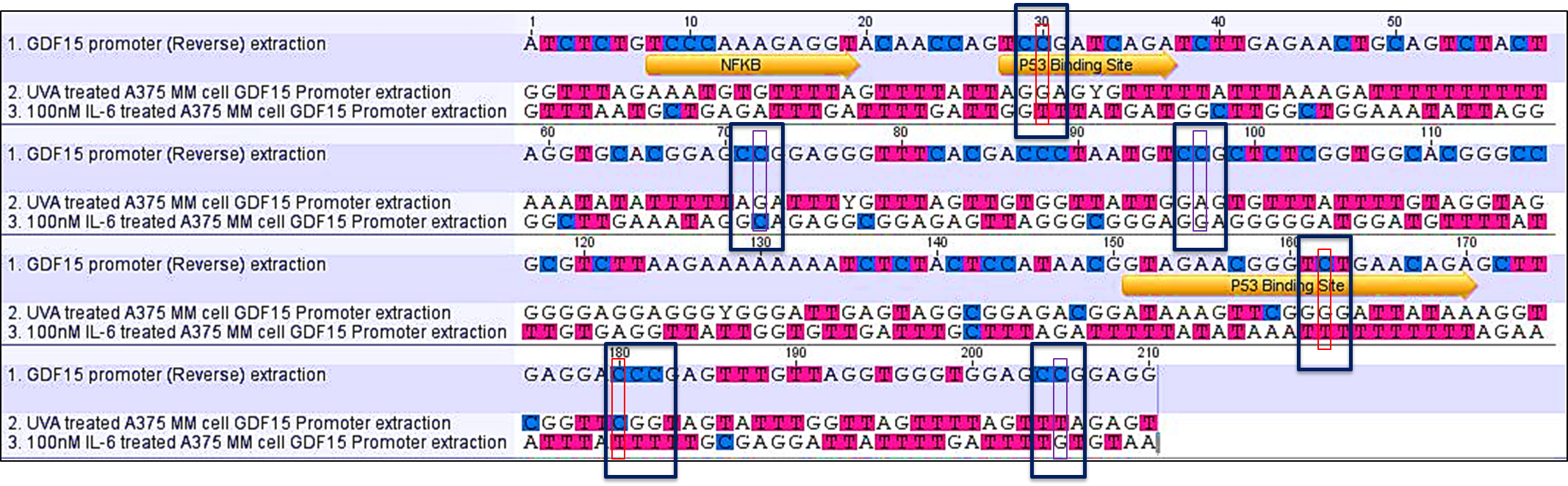


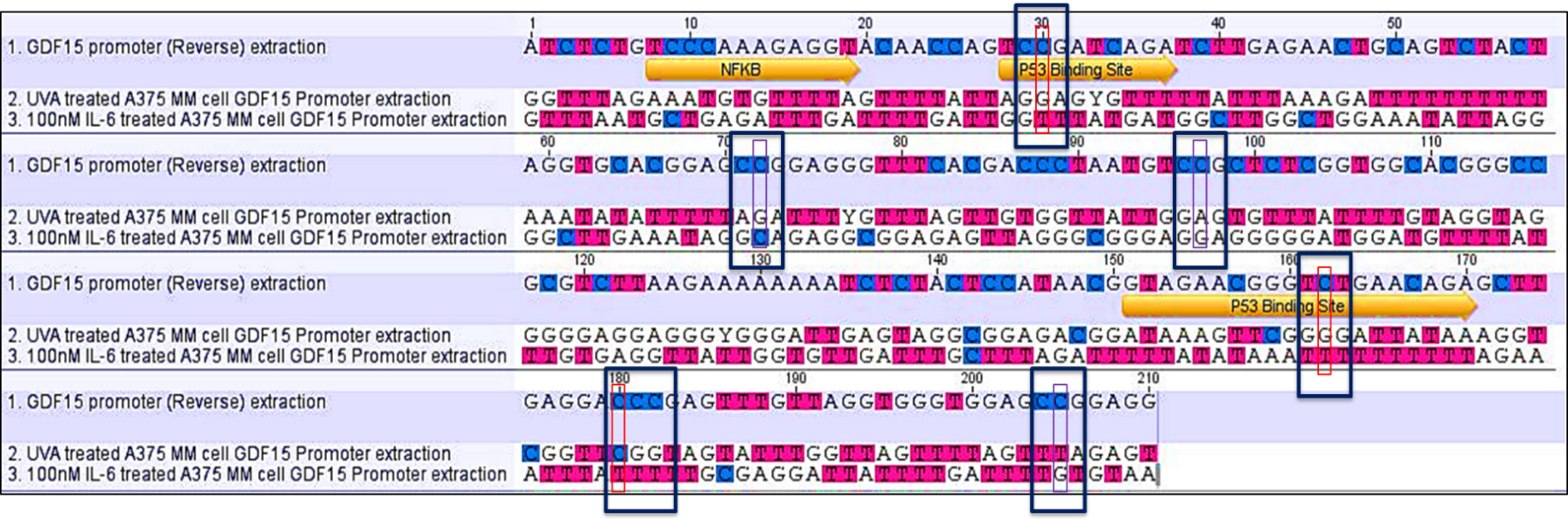


**GDF15 1.**

**Promter (-)**

**UVA (+) 2.**

**UVA+IL-6 (+) 3.**

**
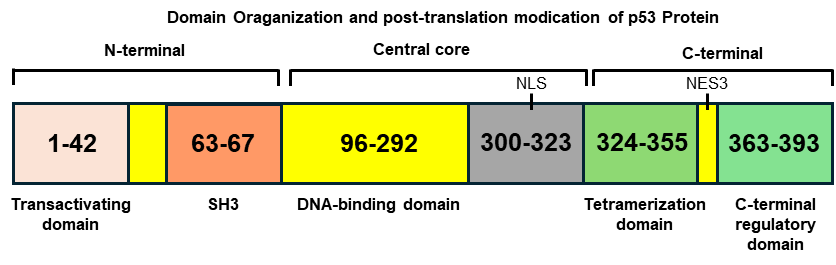
**


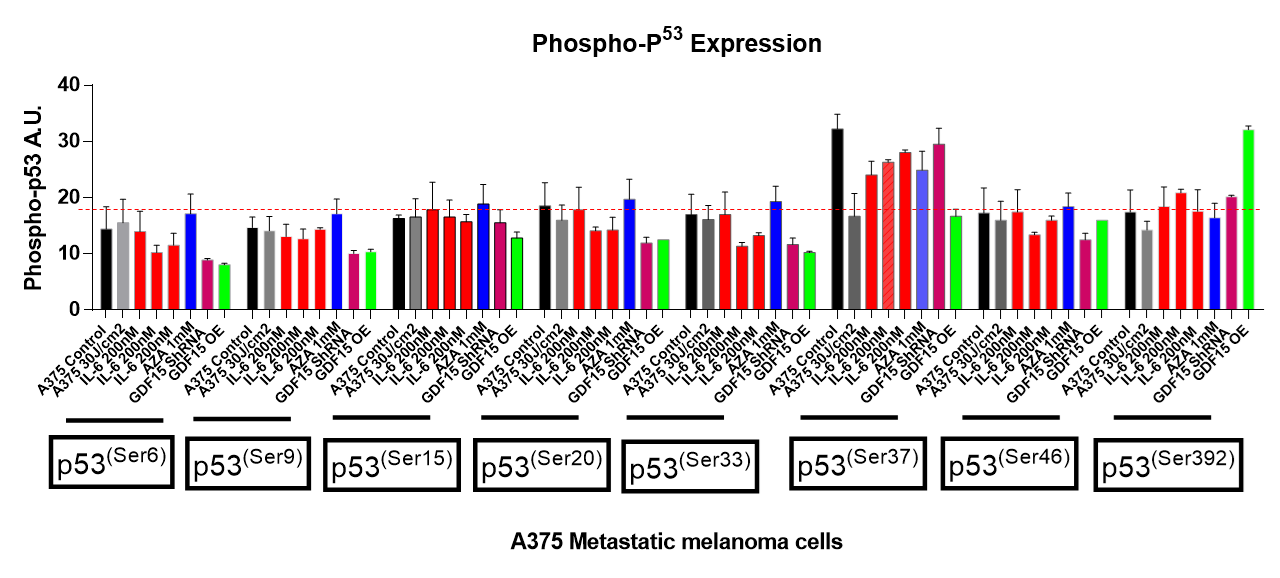


**IL-6 may regulate GDF-15 through P53^ser392^ Phosphorylation in a UVA irradiated melanoma cells**

**(F)**

**(E)**

**TDG may regulates the expression of GDF-15 through hypo-methylation**

**of GDF-15 promoter and could be induced by IL-6 through APOBEC**

**
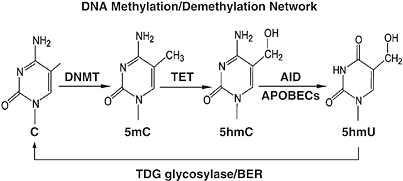

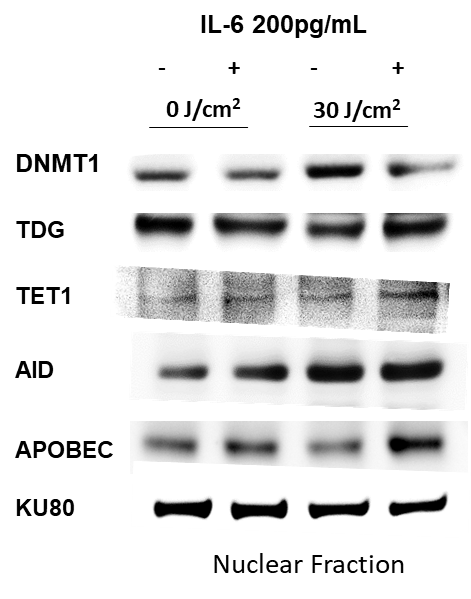
**

**(H))**

**(G)**

**(I)**


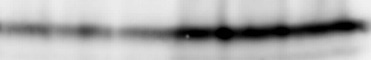

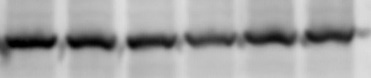


IL-6^WT^

IL-6^TG^

Actin

Anti-IL-6

A375 MM


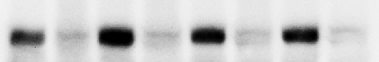

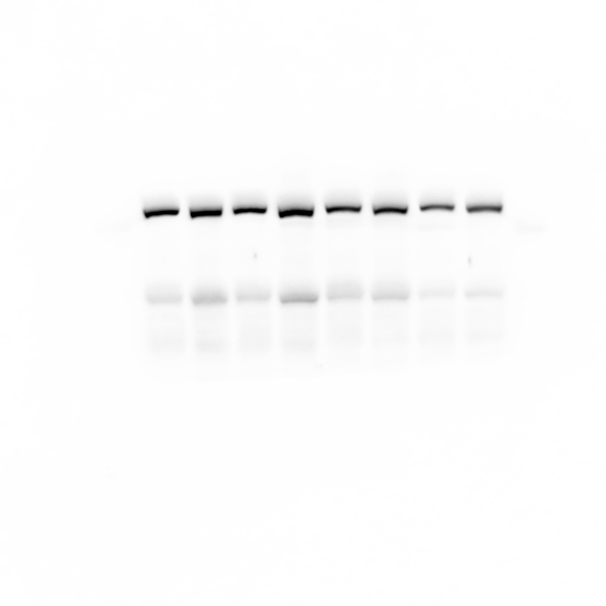


GDF-15^ShRNA1^

GDF-15^TG^

GDF-15^ShRNA1^

GDF-15^ShRNA2^

GDF-15^Scramble^

GDF-15^ShRNA2^

GDF-15^TG^

GDF-15^TG^

Actin

Anti-GDF-15

A375 MM

**(J)**

**Supplementary Table 2**

| **Primer** | **Sequences** |
| --- | --- |
| ***GDF15* Promoter Methyl-Sequencing Primer (F1)** | ***AGG GTT TCT CCA TGT TGG TCA GGC T*** |
| ***GDF15* Promoter Methyl-Sequencing Primer (R1)** | ***TGA AGC CAT CCT CAC AGG ATT CAT*** |
| ***GDF15* Promoter Methyl-Sequencing Primer (F2)** | ***AGA GAA GAG AGG AAA TCC CAT GGG C*** |
| ***GDF15* Promoter Methyl-Sequencing Primer (R2)** | ***CTC CTC CCC CTG CCT GCA GAG TA*** |
| ***GDF15* Promoter Methyl-Sequencing Primer (F3)** | ***GAC TCC CAG GCT GGA ATG GTG TCC TCA*** |
| ***GDF15* Promoter Methyl-Sequencing Primer (R3)** | ***AAT CTT CCC AGC TCT GGT TGG CCC*** |
| **SmaI/MspI Methyl Specific Restriction Primer (F1)** | ***GCG GAG ACG GAC AAA GTC*** |
| **SmaI/MspI Methyl Specific Restriction Primer (F2)** | ***CAG CTC AGA GCC GCA AC*** |
| **SmaI/MspI Methyl Specific Restriction Primer (R)** | ***TTTCCGCAACTCTCGGAATC*** |
| ***GDF15* Promoter Bisulphite Methylated (F)** | ***TTA TGT TCG GGT AAG AAT TTA GGA C*** |
| ***GDF15* Promoter Bisulphite Methylated (R)** | ***GAA ATA CAA CTC TAA AAA TCC CGA A*** |
| ***GDF15* Promoter Bisulphite Unmethylated (F)** | ***TAT GTT TGG GTA AGA ATT TAG GAT G*** |
| ***GDF15* Promoter Bisulphite Unmethylated (R)** | ***TTCA AAA TAC AAC TCT AAA AAT CCC A*** |
| ***GDF15* Pyrosequencing PCR Probe (F1)** | ***GGT TTA GAA ATG TGT TTT AGT TTT ATT AGG*** |
| ***GDF15* Pyrosequencing PCR Probe (R1)** | ***AAA CCA TTC ACC CTC CTA AAT TCT TAC C*-Biotin** |
| ***GDF15* Pyrosequencing PCR Probe (F2)** | ***AGG AAG GTG AAT GGT TTT TAG ATG T*** |
| ***GDF15* Pyrosequencing PCR Probe (R2)** | ***ACC CCC AAC CTA ATT AAC AAA TCC T*-Biotin** |
| ***GDF15* Pyrosequencing (Sequencing Primer)** | ***GTT GTG GTT ATT GGA GT*** |
| ***GDF15* promoter p53 EMSA 6 (F)-Demethylated** | **CAA CCT GCA CAG CCA TGC CC-Biotin** |
| ***GDF15* promoter p53 EMSA 6 (F)- Methylated** | **GTT GGA ^5^CGT GT^5^C GGT A^5^CG GG-Biotin** |
| ***GDF15* promoter p53 EMSA 6 (F)-COLD** | **CAA CCT GCA CAG CCA TGC CC** |
| ***GDF15* Promoter (F)** | **CGA TCA TCT TGC CCA GAC TTG TCT AGT CCC AGC TCA GA** |
| ***GDF15* Promoter (R)** | **TTA AGA CTC CCA GGG CCC TTT GAA CGC GCC** |
| ***GDF15 LNA unmethylated Oligonucleotide*** | **5TEX615-T+C+G+C+C+T+C+T+G+C+C** |
| ***GDF15 LNA methylated Oligonucleotide*** | **5Flour-G+iMe-dC+G+G+A+G+A+iMe-dC+G+G** |

**Legend to Supplementary figure 2.**

(**A**) Graphical representation of GDF-15 promoter from -1000 to + 4kb. (**B**) MSRP (Methylation Sensitive Restriction PCR) to analyze DNA methylation patterns by employing methylation-sensitive restriction enzymes (MSREs) like SmaI, in a PCR-based assay. Digested genomic DNA with an SmaI, which preferentially cleaves unmethylated CpG sites, followed by PCR amplification of the remaining DNA fragments gives peaks in melting plot.  When the DNA gets methylated, SmaI will not be able to digest and hence no amplification and vice versa. Here, in supplementary figure 2B, left panel has no amplification as there are no enzymes, middle panel methylated (30J) and unmethylated (0J) as MspI is insensitive to methylation, both have same amplification. Right panel (MspI + SmaI) has slightly higher amplification due to hypomethylation of GDF-15 promoter due to 200pg Rh IL-6, followed by both SmaI and MspI amplification of the DNA sequences. (Lower left panel) shows similar amplification at 0J/cm^2^ and no amplification at 30J/cm^2^ by methylation sensitive enzyme SmaI. (Lower middle panel) shows further upregulation after treatment with 200pg IL-6 showing hypomethylation of GDF-15 promoter and restriction mediated amplification by SmaI. (**C**) Human GDF-15 promoter sequence from +247 to -1000 kb with MSRE sites. (**D**) Homology representation in between Bisulphite converted *GDF-15* Gene promoter sequence taken from A375 melanoma treated with UVA (2), treated with UVA + IL-6 200ng (3) and control human GDF-15 sequence taken from NCBI database. CpG islands are marked where the C to T deamination took place which are most P53 transcription factor binding sites. These data indicate that increasing IL-6 supplementation of UVA irradiated melanoma cells can specifically hypomethylate the GDF-15 promoter, while UVA exposure induces global DNA hypermethylation. (**E**) Domain structure of p53 protein. (**F**) IL-6 may regulate GDF-15 through P53^ser392^ Phosphorylation in a UVA irradiated melanoma cell. Various p53 serine kinases ELISA were subjected to UVA irradiated and non-irradiated cell lysates as well as using Rh IL-6. A375 TG and A375 KDN served as controls. We observed that p53Ser392 TG showed the highest p53Ser392 expression as well as A375 GDF-15 ShRNA shows corresponding downregulation. Figure **(G & H)** denotes the pathway and DNA demethylation as well as western blot showing enzymes like APOBEC may regulate the expression of GDF-15 through hypo-methylation of GDF-15 promoter and could be induced by IL-6 through APOBEC. **(I & J)** Shows the western blot confirmation of GDF-15^KDN^ knockdown in A375 melanoma cells as well as IL-6 overexpression in A375 melanoma as A375^TG^

**Legend to Supplementary Table 2.**

Supplementary Table 2 shows the Methyl sequencing primer for GDF-15 promoter Forward and Reverse, SmaI/MspI Methyl Specific Restriction Primers (MSRP) for GDF-15 promoters. Bisulphite methylated and Unmethylated GDF-15 Promoter Primer sequence. GDF-15 Biotinylated Pyrosequencing PCR primer for GDF-15 promoter. GDF-15 promoter primers. EMSA probes methylated, unmethylated and Cold.
