# Supplementary material for: The dark side of the light (UVA): melanoma microenvironment and cell survival strategies
Source: Cell Death Discov. 2025 Oct 20;11:466. doi: 10.1038/s41420-025-02751-y (PMC12537921; doi:10.1038/s41420-025-02751-y)
Supplement: Supplementary file 2 — Western Blots as supplementary files [file 41420_2025_2751_MOESM2_ESM.pdf]

Figure 2. J Anti_IL-6 antibody

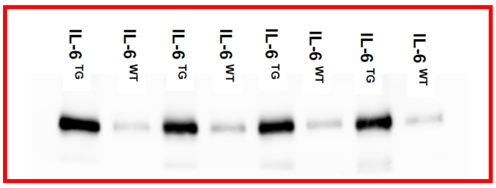

Figure 2. J Anti_Actin antibody

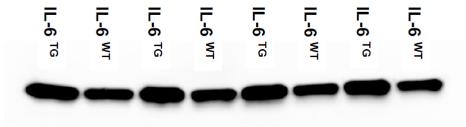

Figure 2. J Anti_pATM antibody

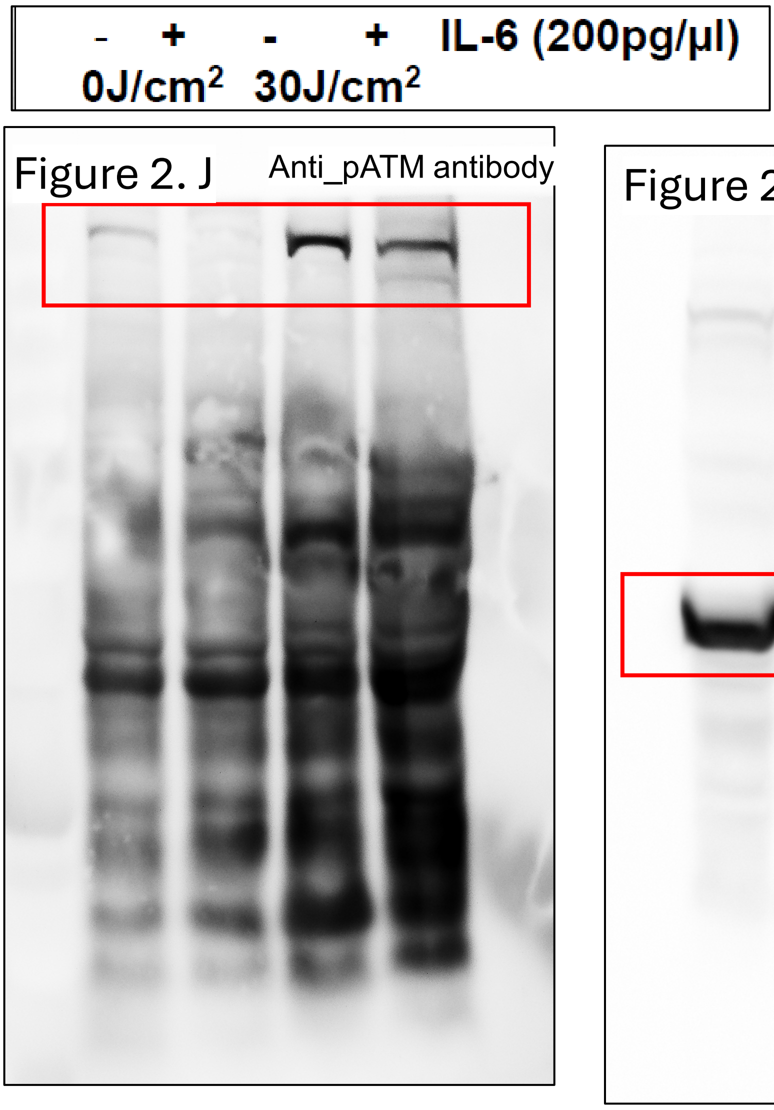

Figure 2. J Anti_Actin antibody

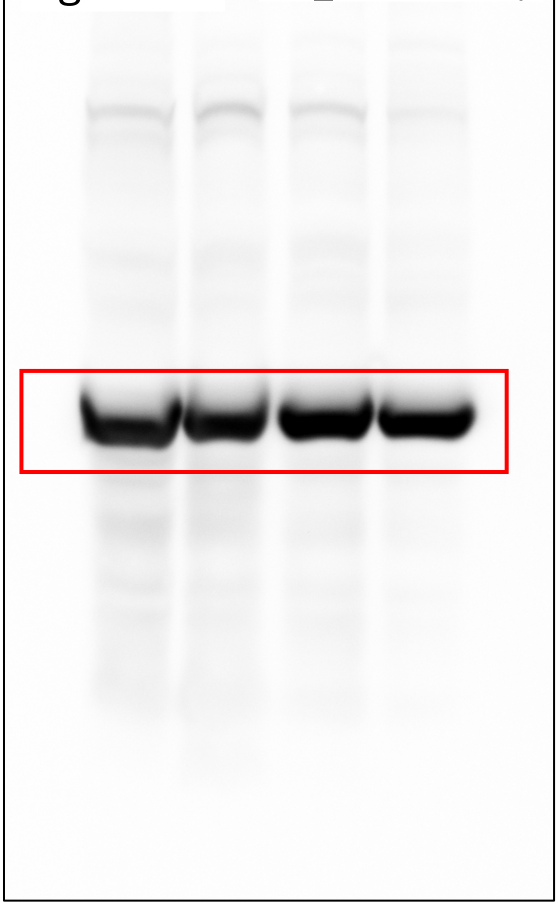

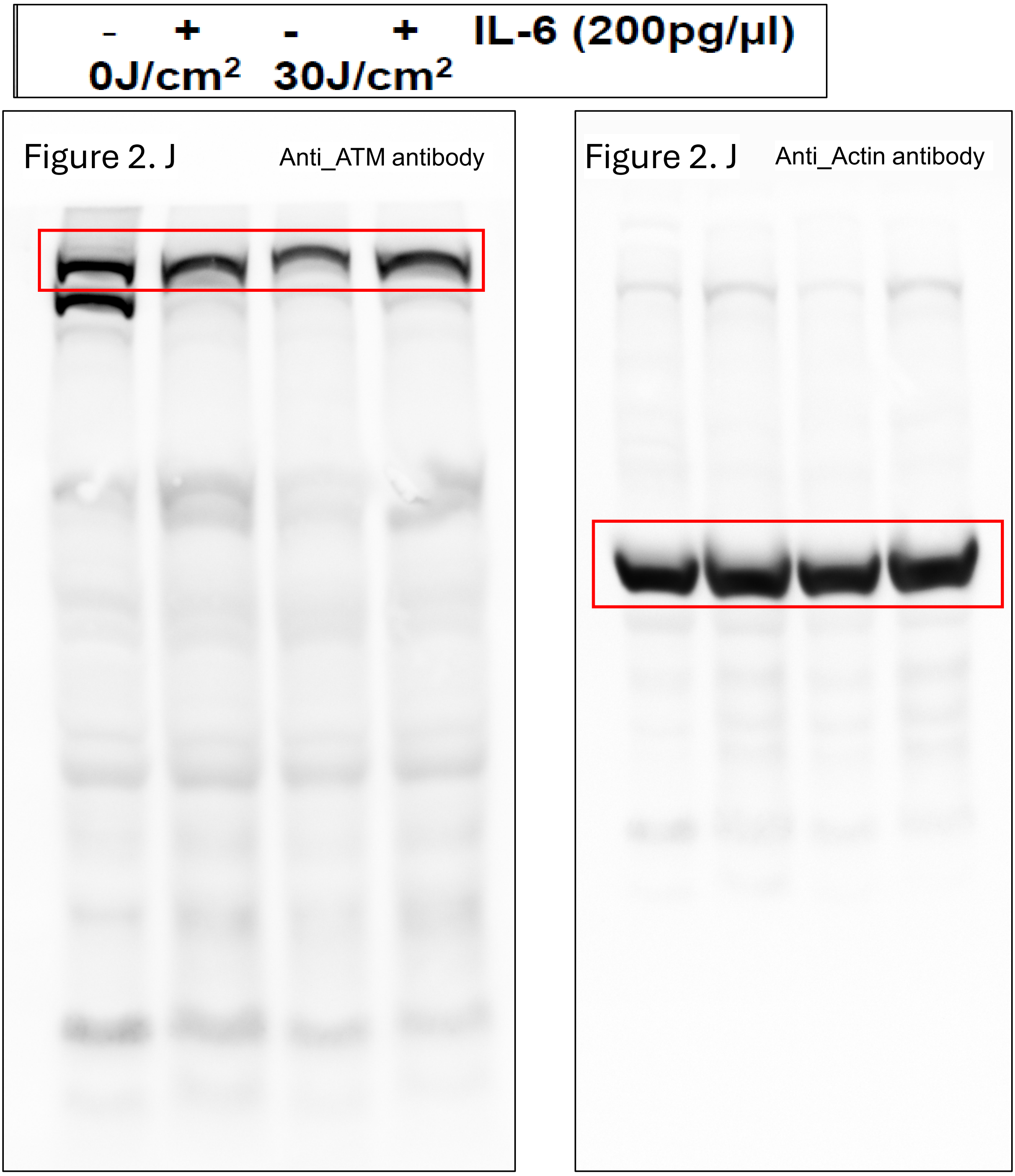
- + - + IL-6 (200pg/μl)
0J/cm2 30J/cm2
Figure 2. J
Anti_ATM antibody
Figure 2. J
Anti_Actin antibody

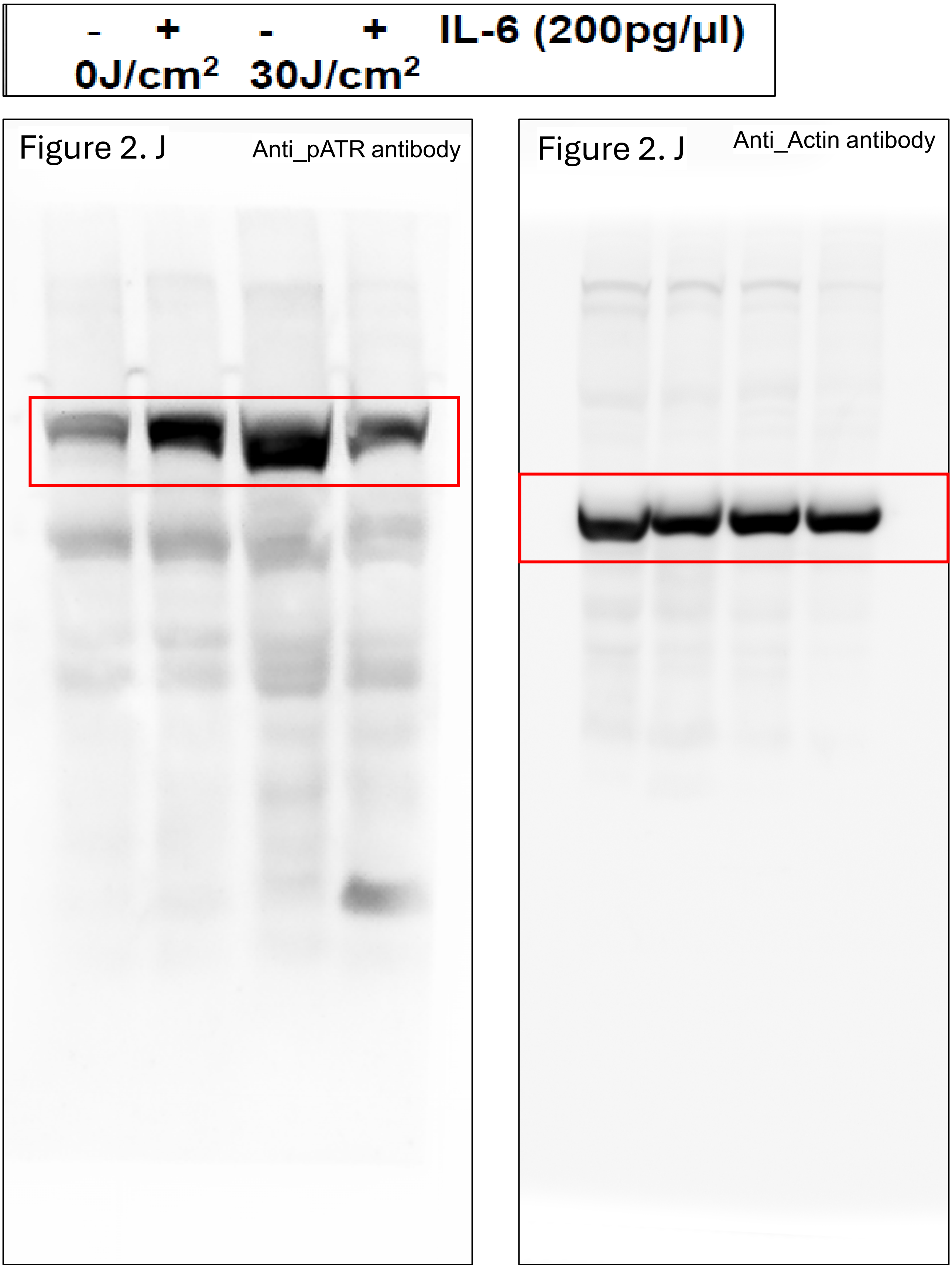

- + - + IL-6 (200pg/μl)
0J/cm2 30J/cm2
Figure 2. J
Anti_pATR antibody
Figure 2. J
Anti_Actin antibody

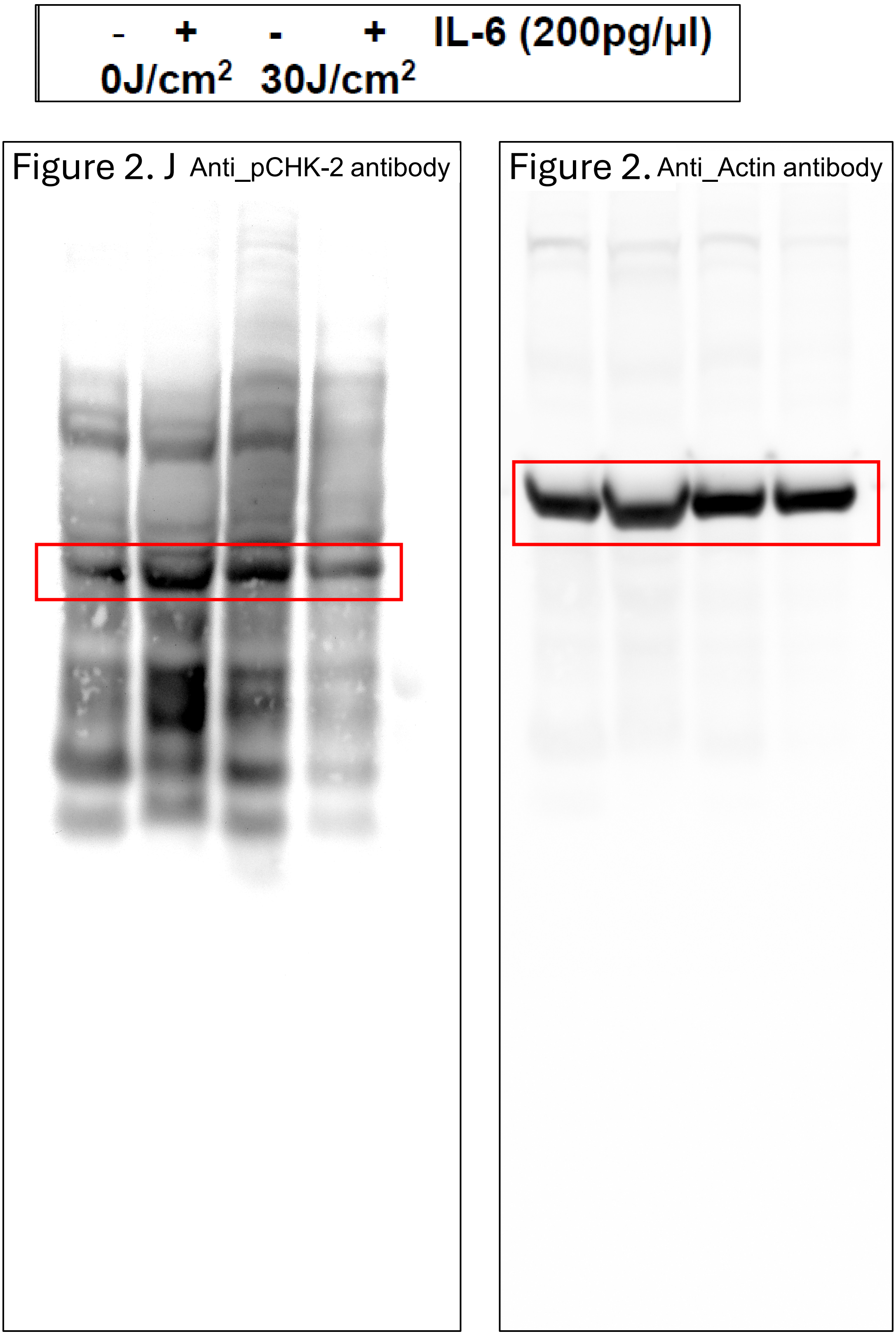
- + - + IL-6 (200pg/μl)
0J/cm2 30J/cm2
Figure 2. J Anti_pCHK-2 antibody
Figure 2. Anti_Actin antibody

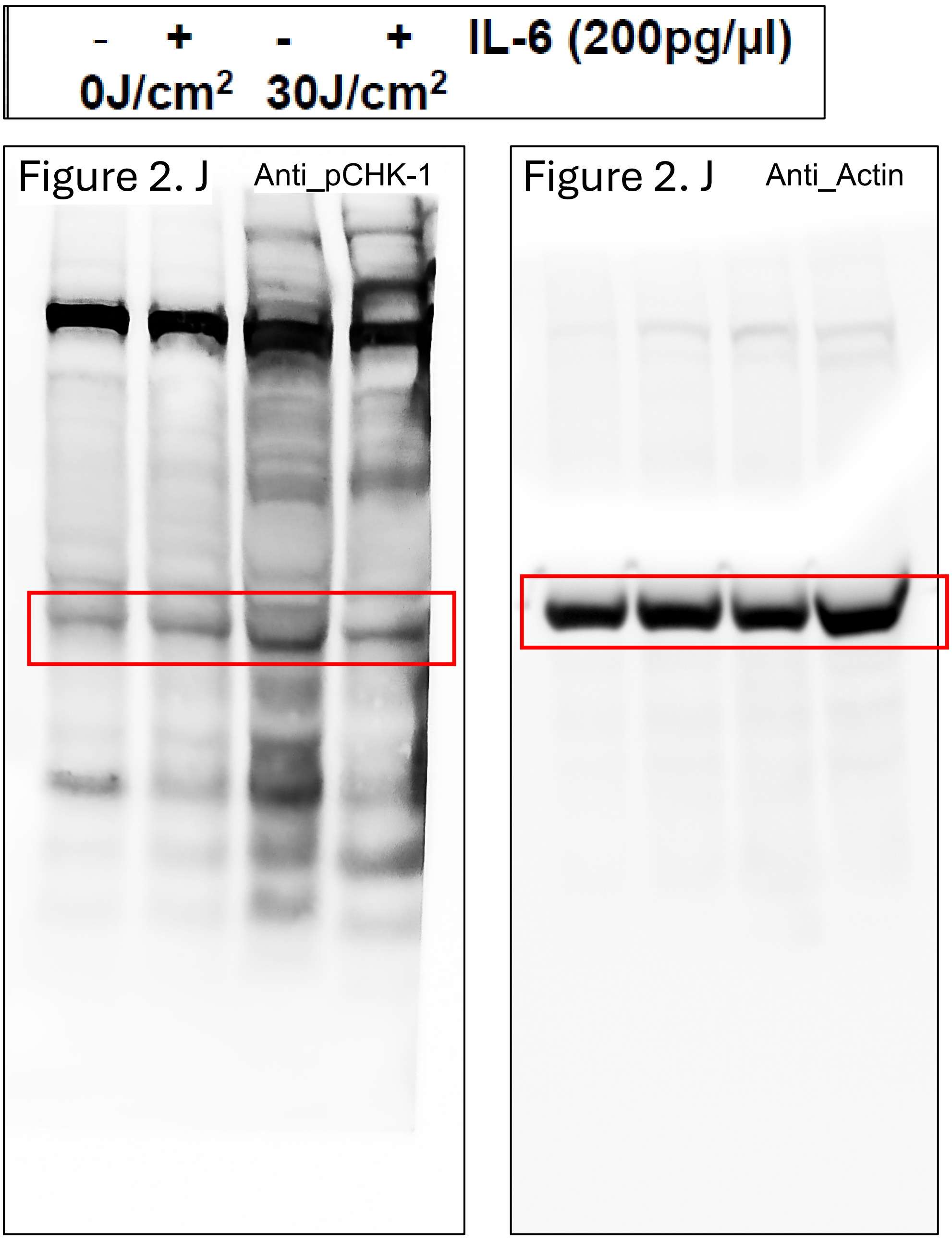
- + - + IL-6 (200pg/μl)
0J/cm2 30J/cm2
Figure 2. J
Anti_pCHK-1
Figure 2. J
Anti_Actin

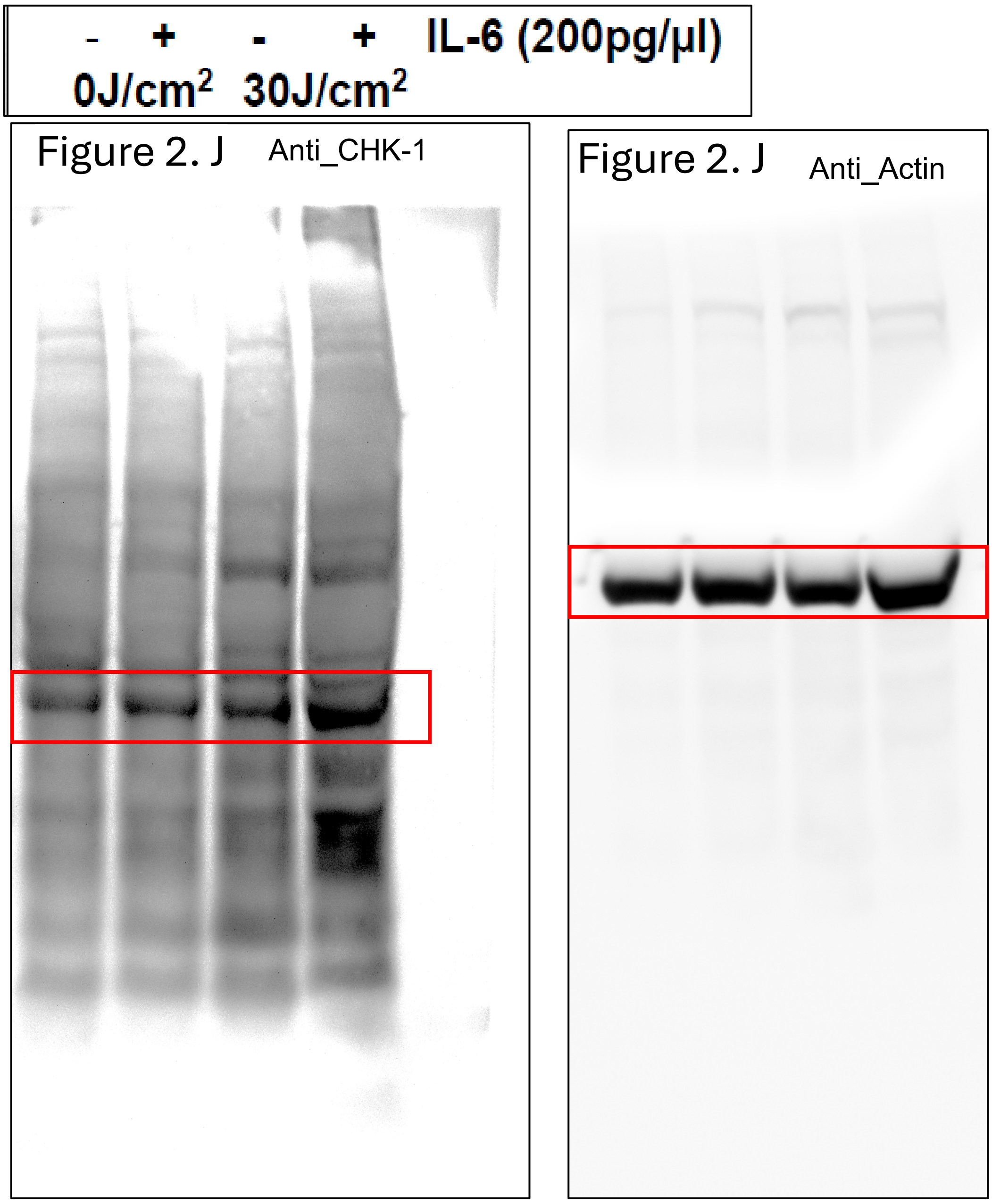
- + - + IL-6 (200pg/µl)
0J/cm2 30J/cm2
Figure 2. J
Anti_CHK-1
Figure 2. J
Anti_Actin

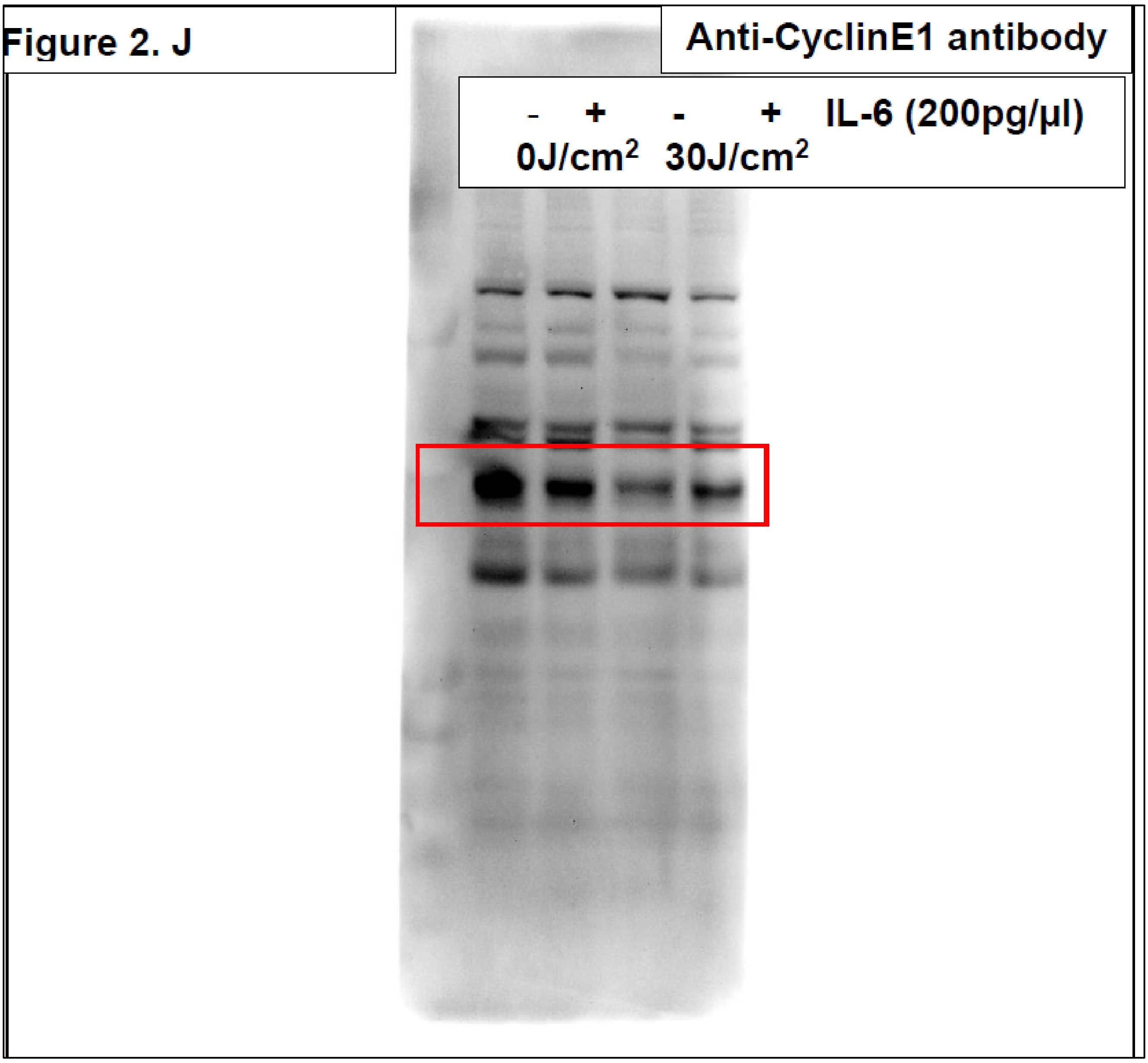
Figure 2. J
Anti-CyclinE1 antibody
- + - + IL-6 (200pg/μl)
0J/cm2 30J/cm2

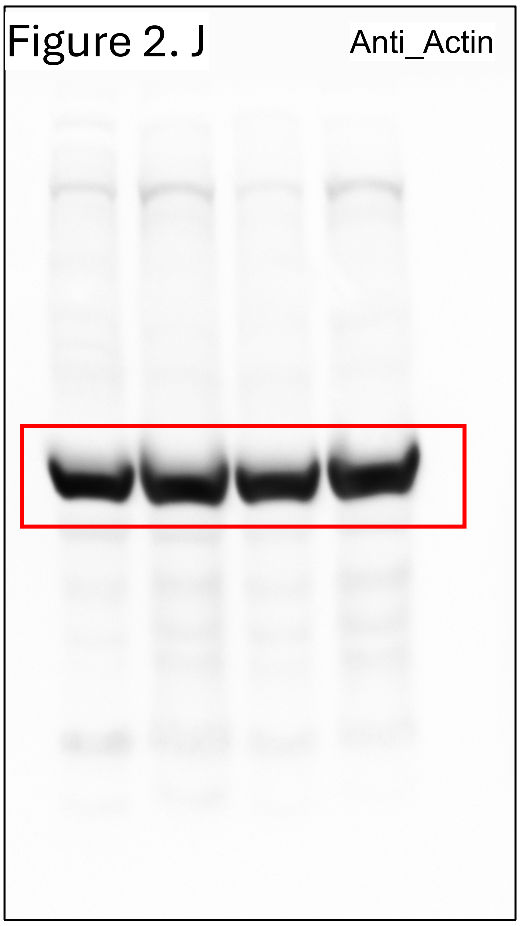
Figure 2. J
Anti_Actin

Figure 2. J

Anti-Cdc25a antibody

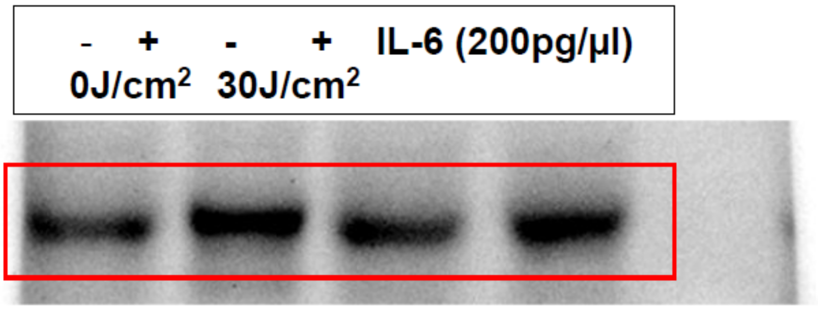

Figure 2. J

Anti_Actin

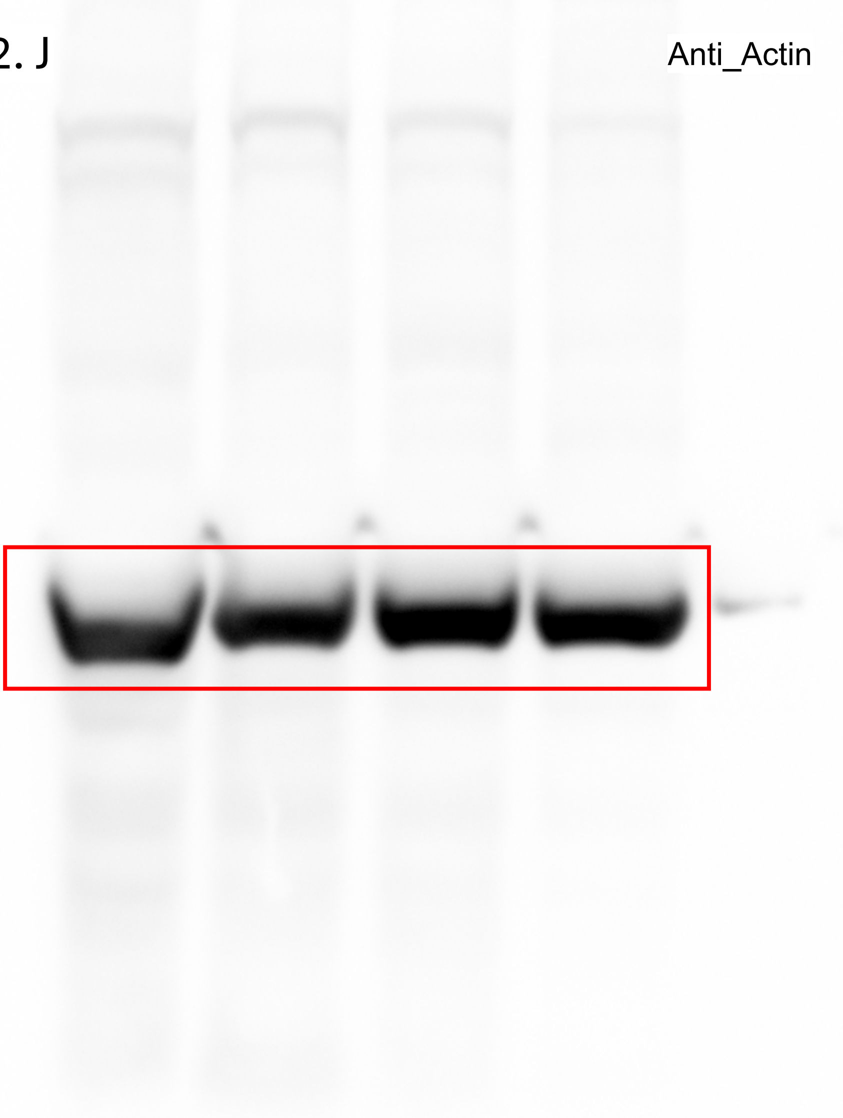

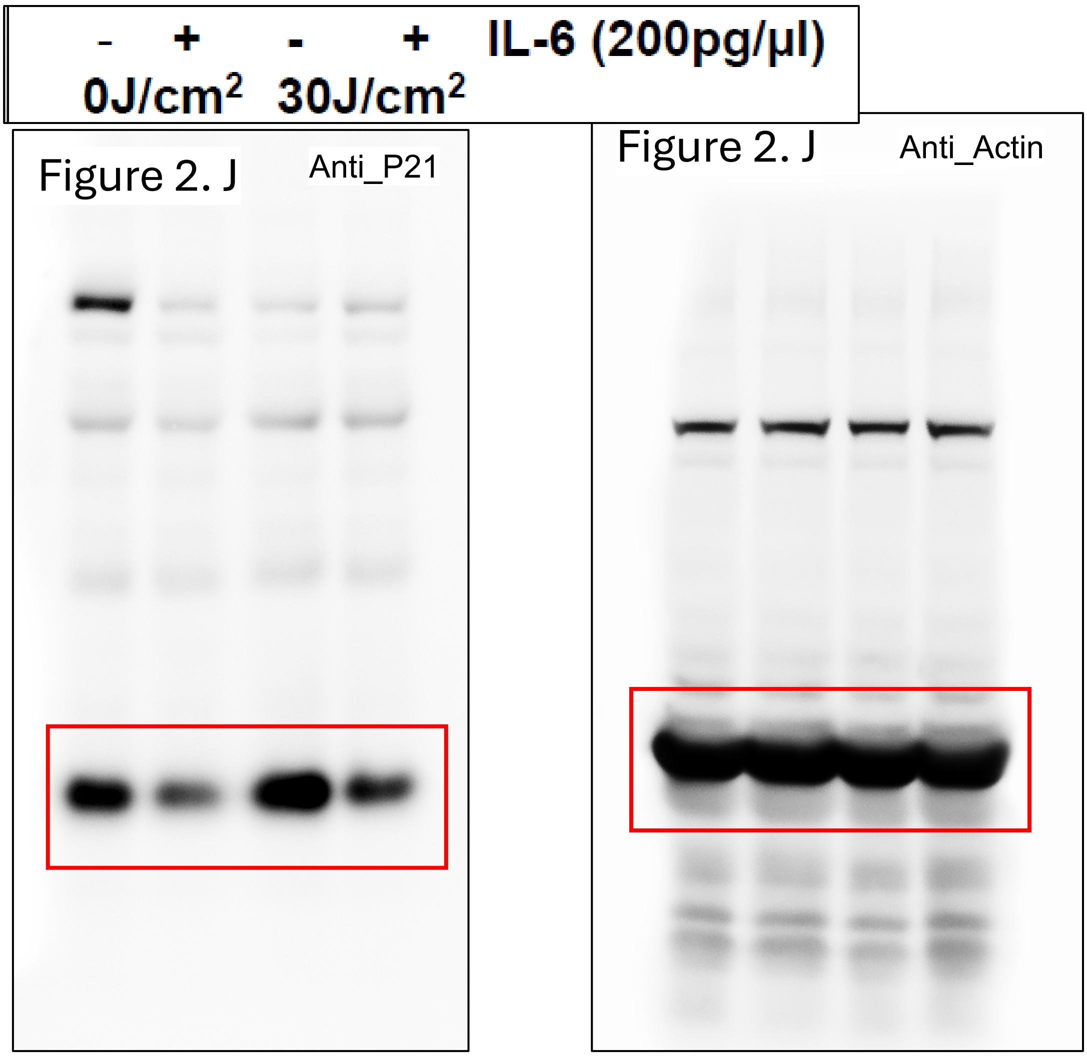
- + - + IL-6 (200pg/μl)
0J/cm2 30J/cm2
Figure 2. J
Anti_P21
Figure 2. J
Anti_Actin

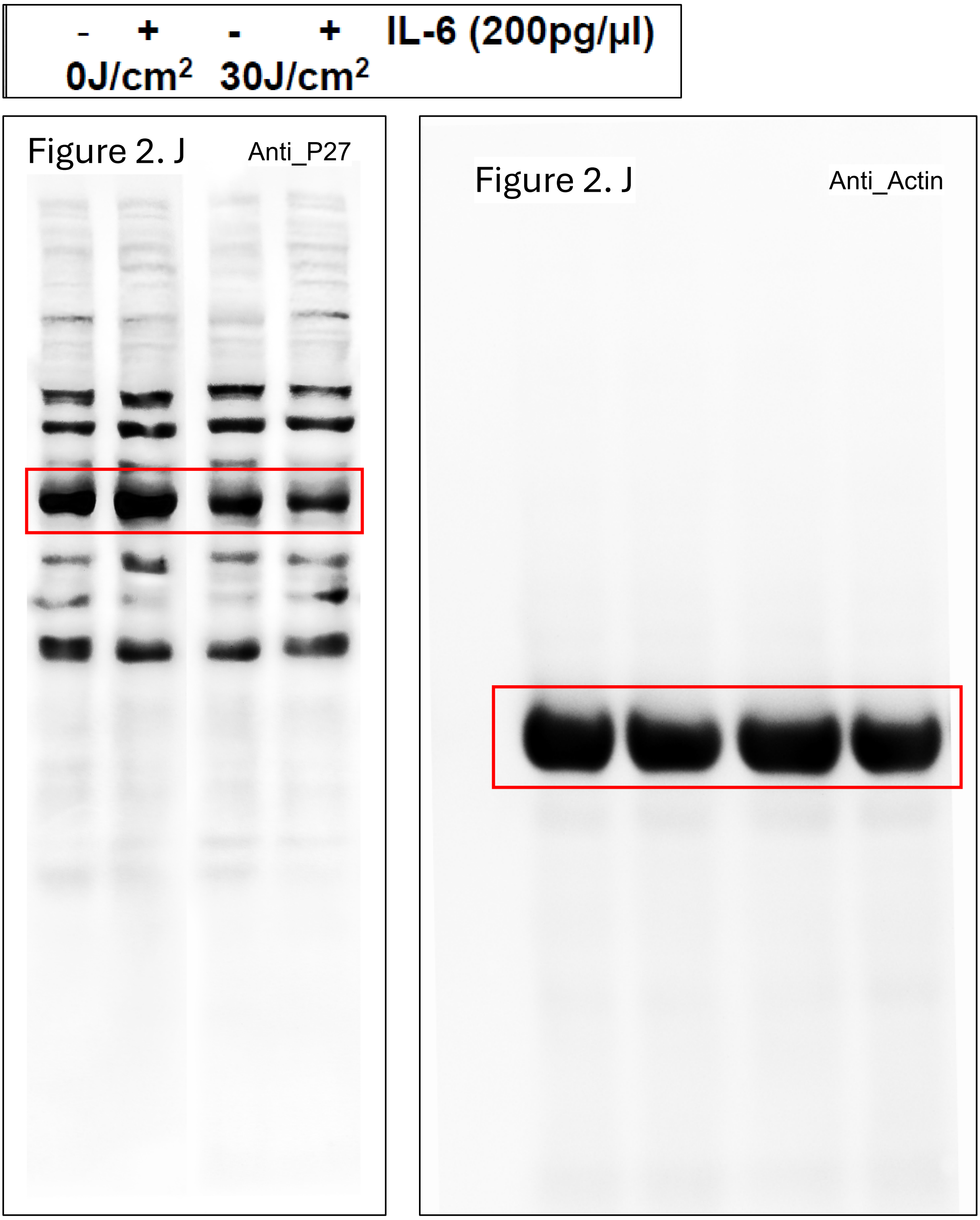
- + - + IL-6 (200pg/μl)
0J/cm2 30J/cm2
Figure 2. J
Anti_P27
Figure 2. J
Anti_Actin

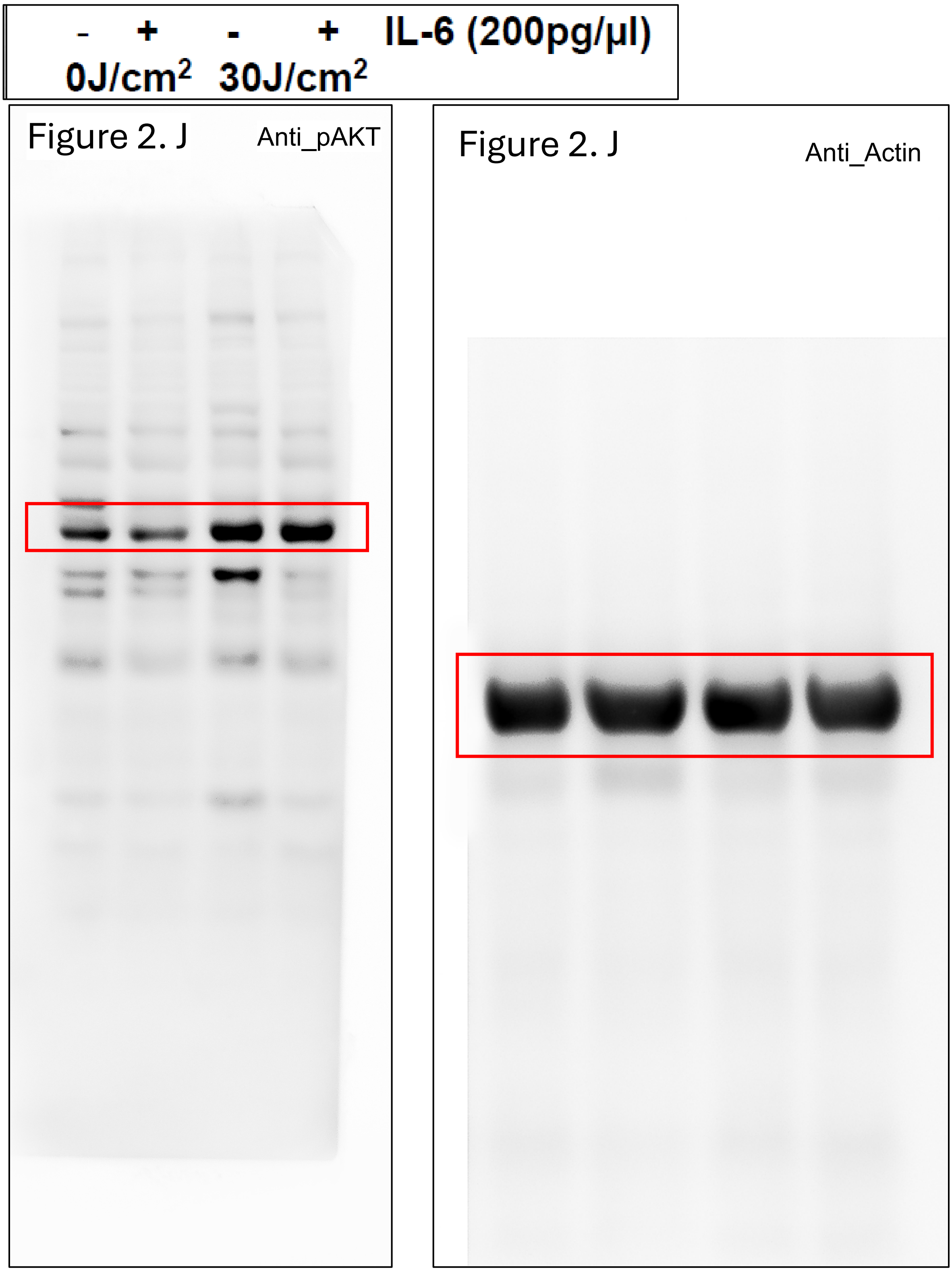
- + - + IL-6 (200pg/µl)
0J/cm2 30J/cm2
Figure 2. J
Anti_pAKT
Figure 2. J
Anti_Actin

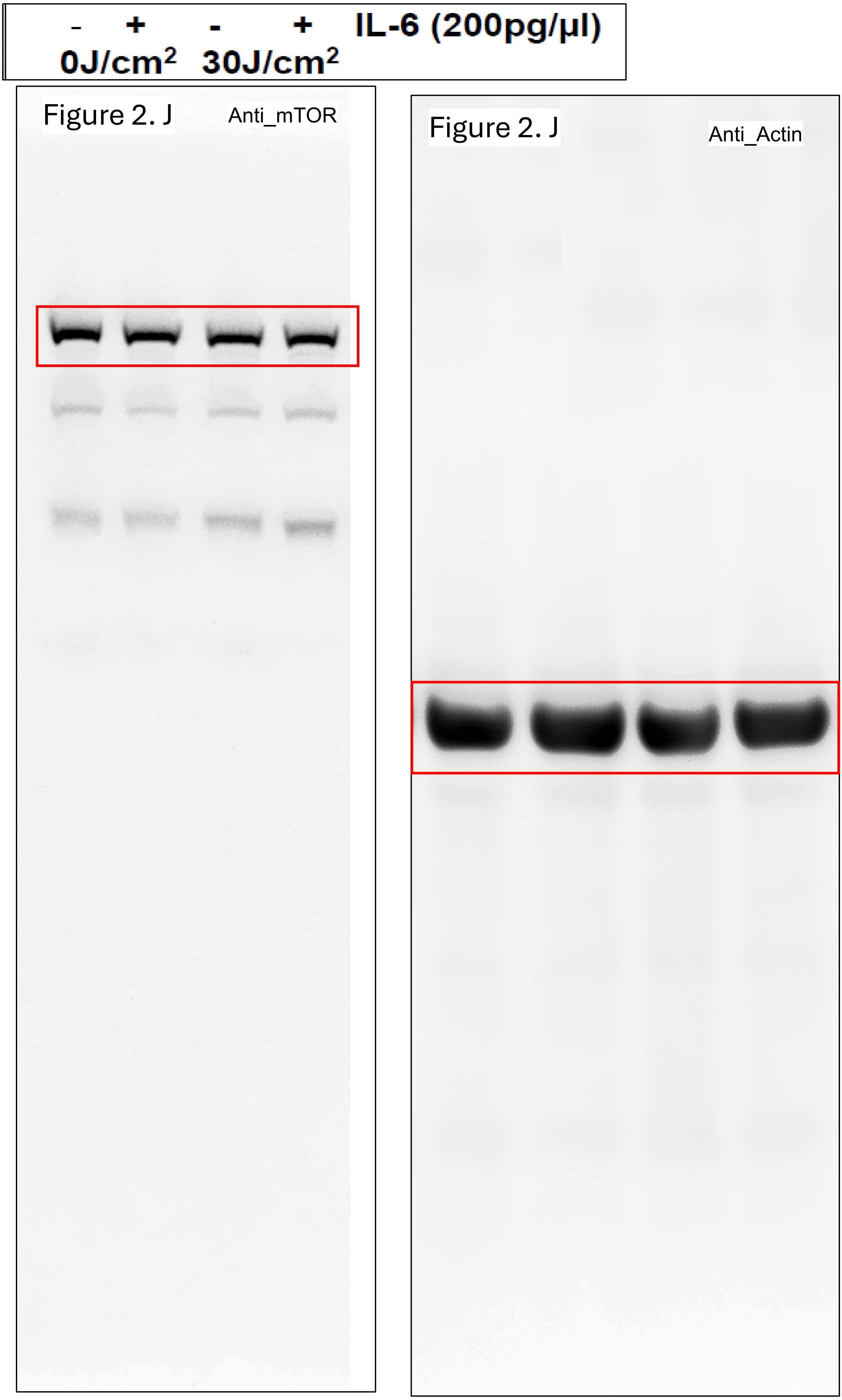
- + - + IL-6 (200pg/μl)
0J/cm2 30J/cm2
Figure 2. J
Anti_mTOR
Figure 2. J
Anti_Actin

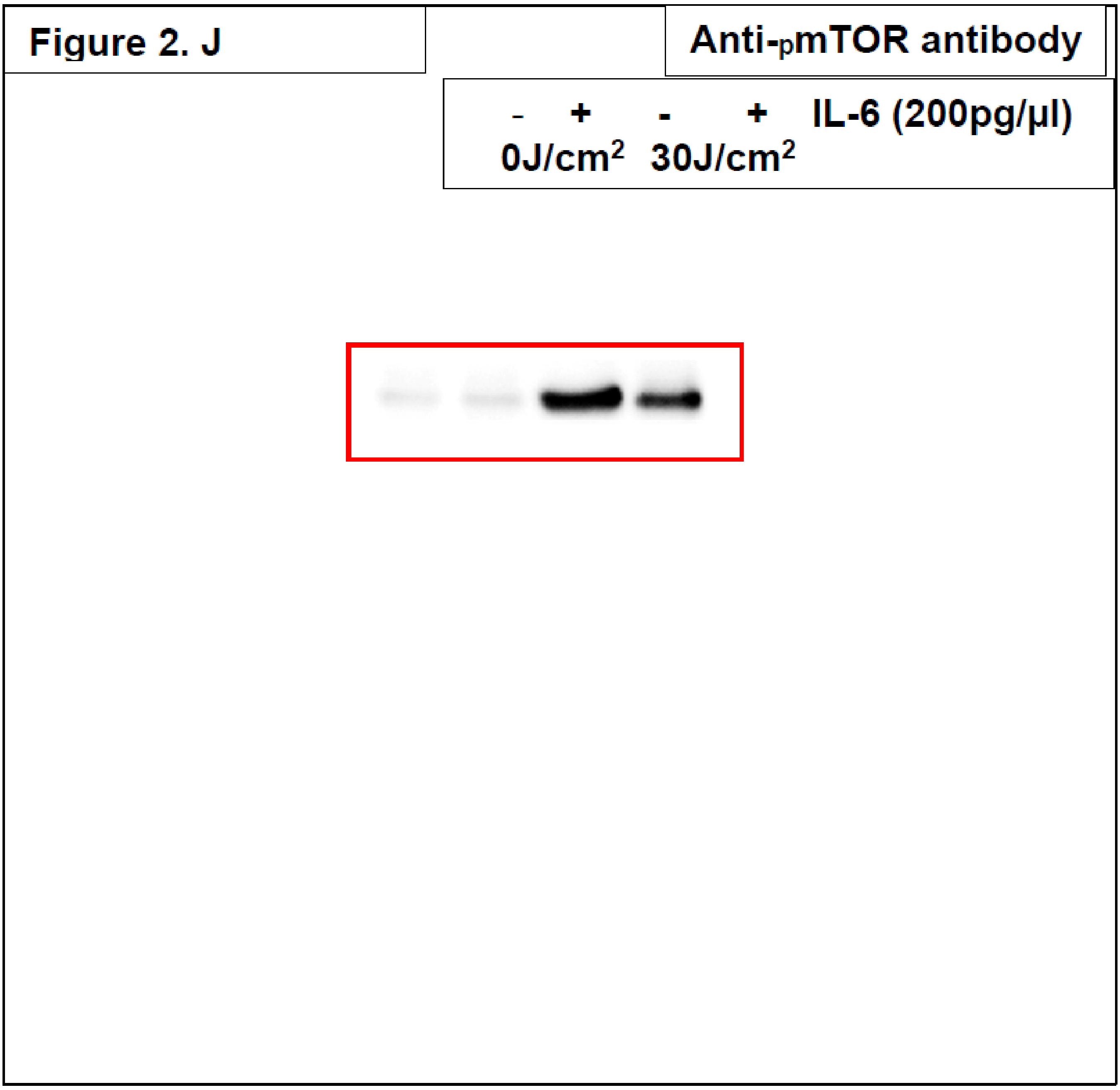
Figure 2. J
Anti-pmTOR antibody
- + - + IL-6 (200pg/µl)
0J/cm2 30J/cm2

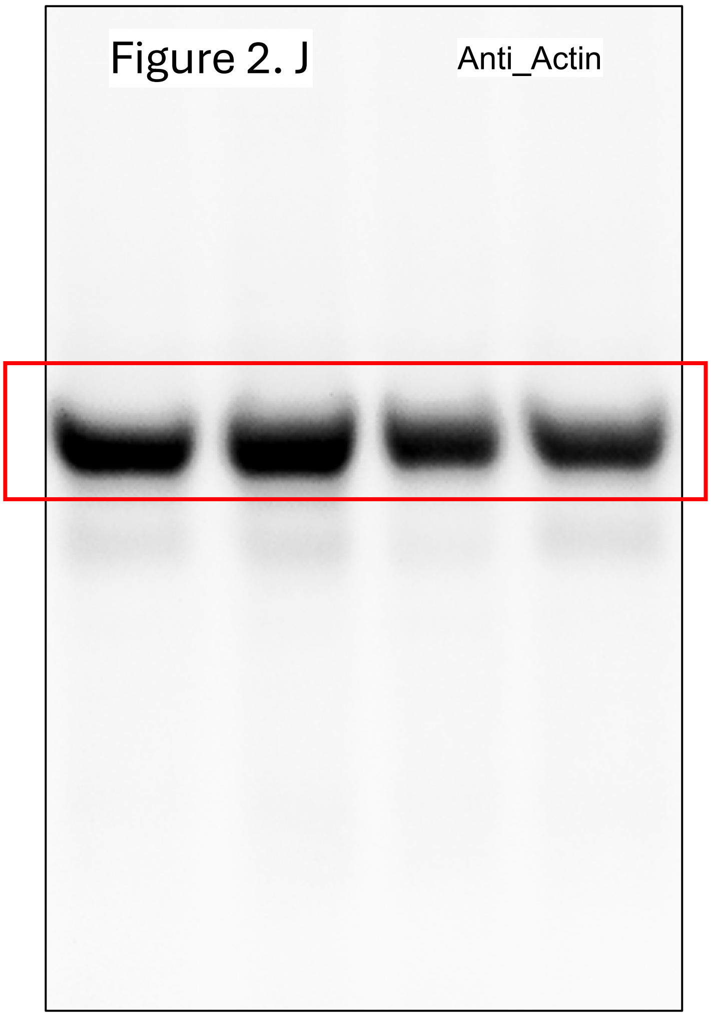
Figure 2. J
Anti_Actin

Figure 2. J

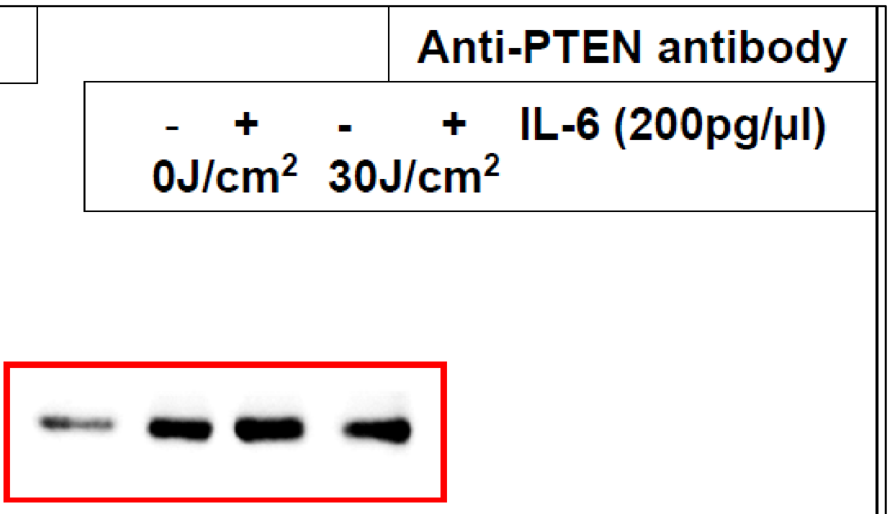

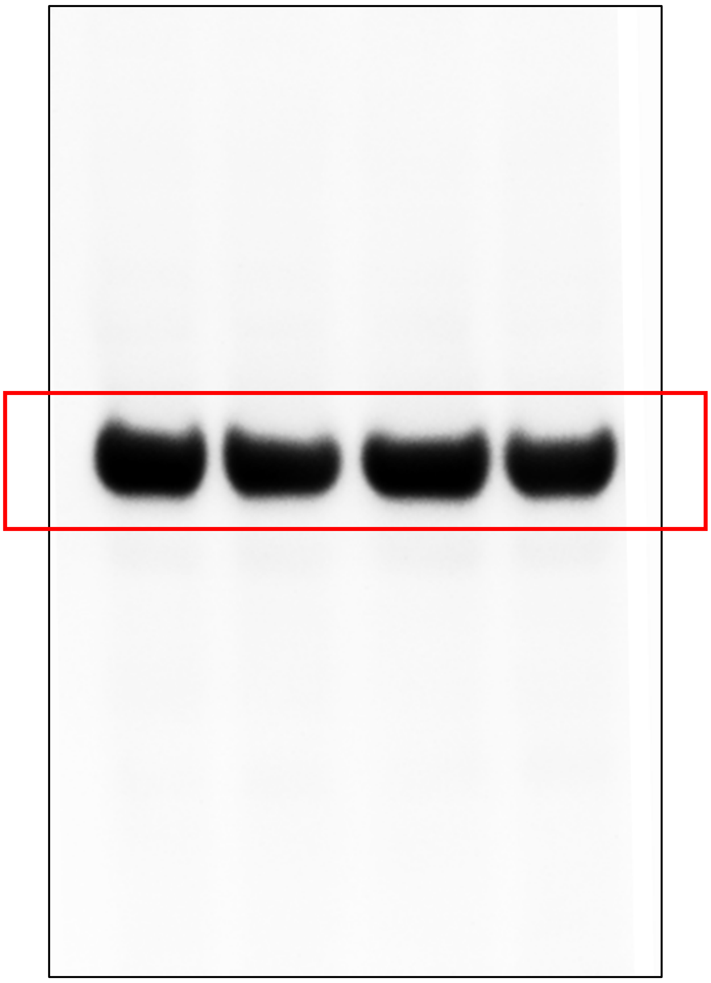

Figure 2. J

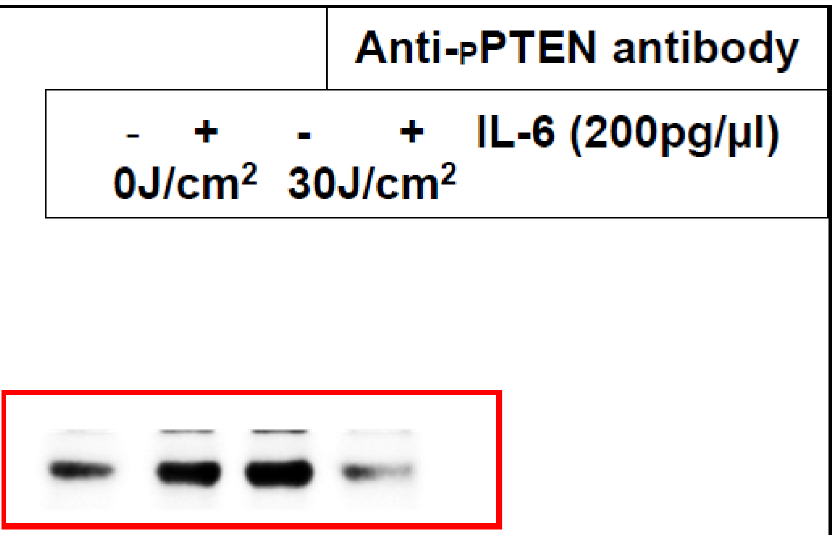

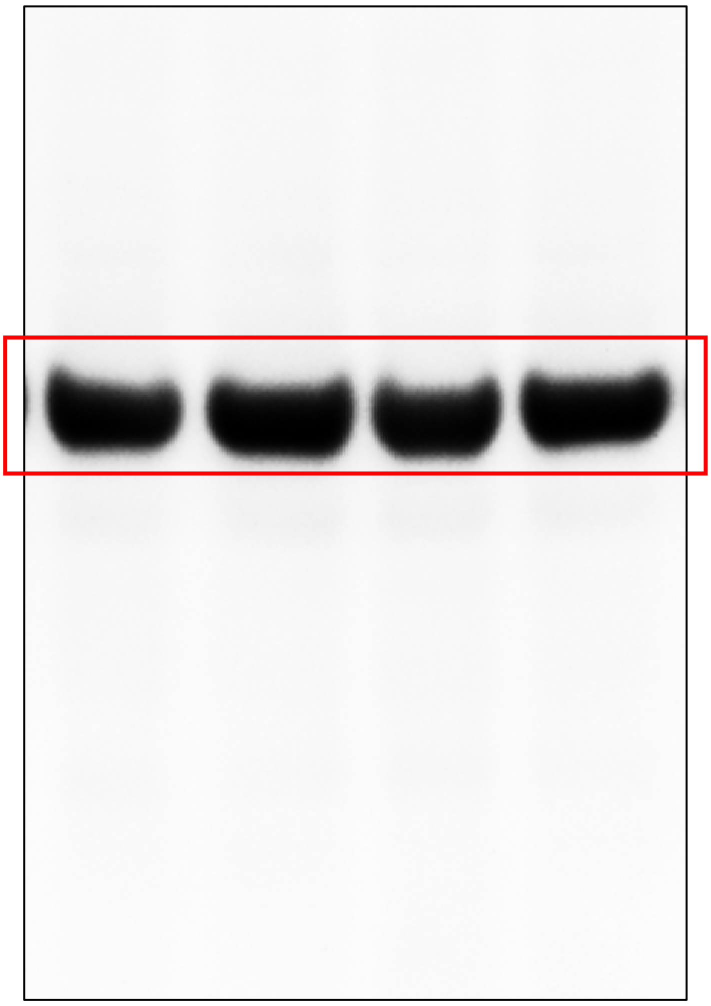

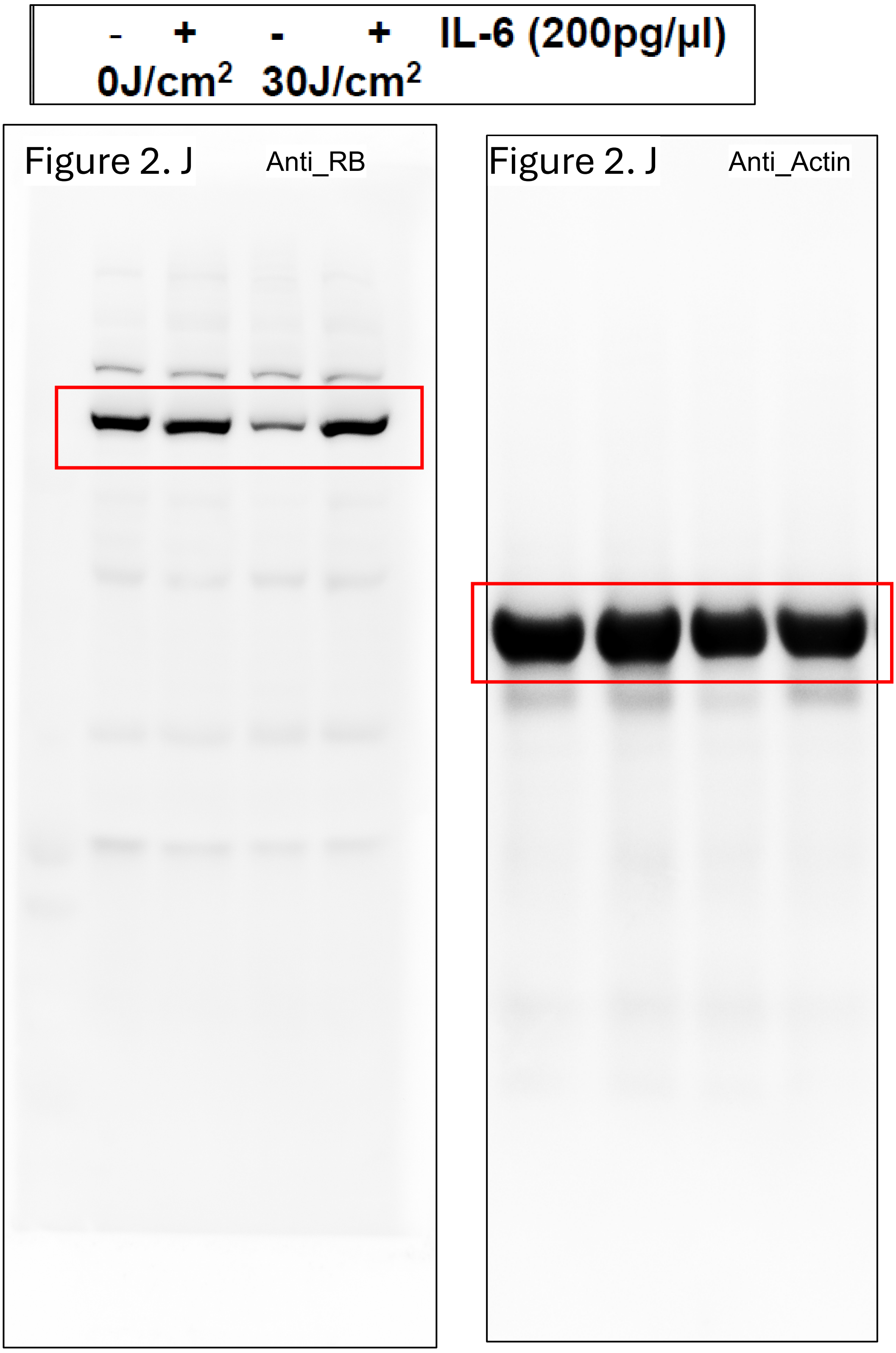
- + - + IL-6 (200pg/µl)
0J/cm2 30J/cm2
Figure 2. J
Anti_RB
Figure 2. J
Anti_Actin

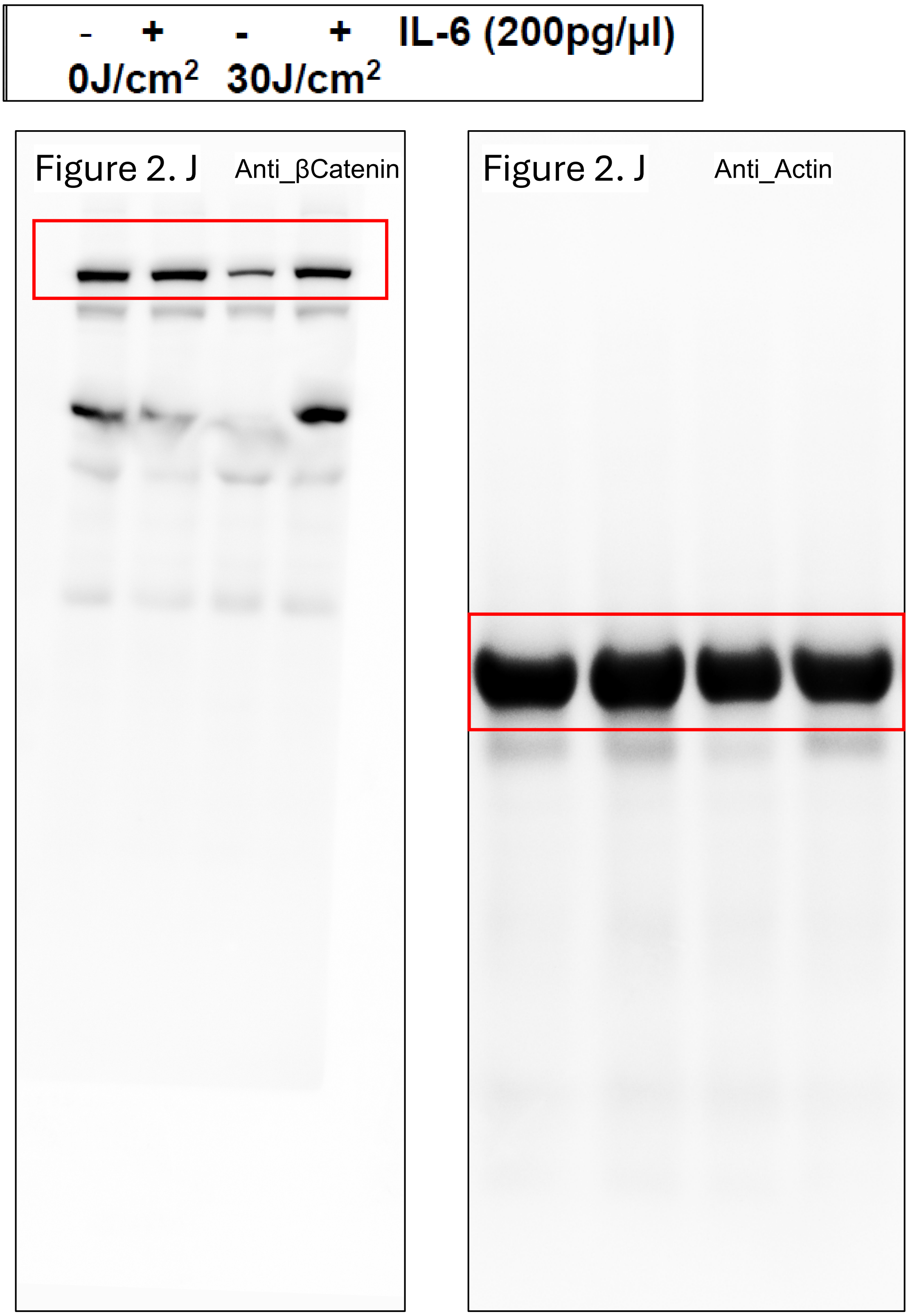

- + - + IL-6 (200pg/μl)
0J/cm2 30J/cm2
Figure 2. J
Anti_βCatenin
Figure 2. J
Anti_Actin

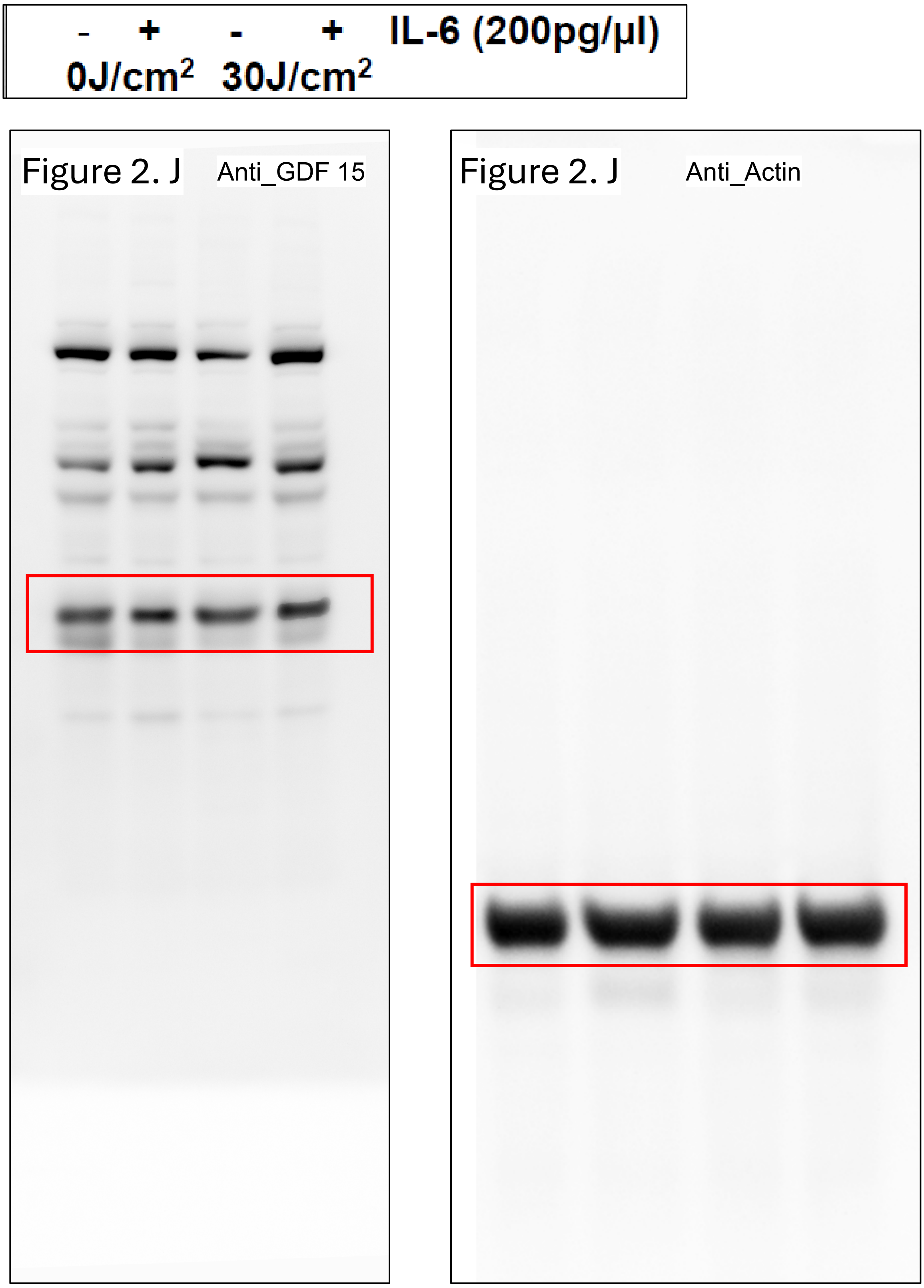
- + - + IL-6 (200pg/μl)
0J/cm2 30J/cm2
Figure 2. J
Anti_GDF 15
Figure 2. J
Anti_Actin

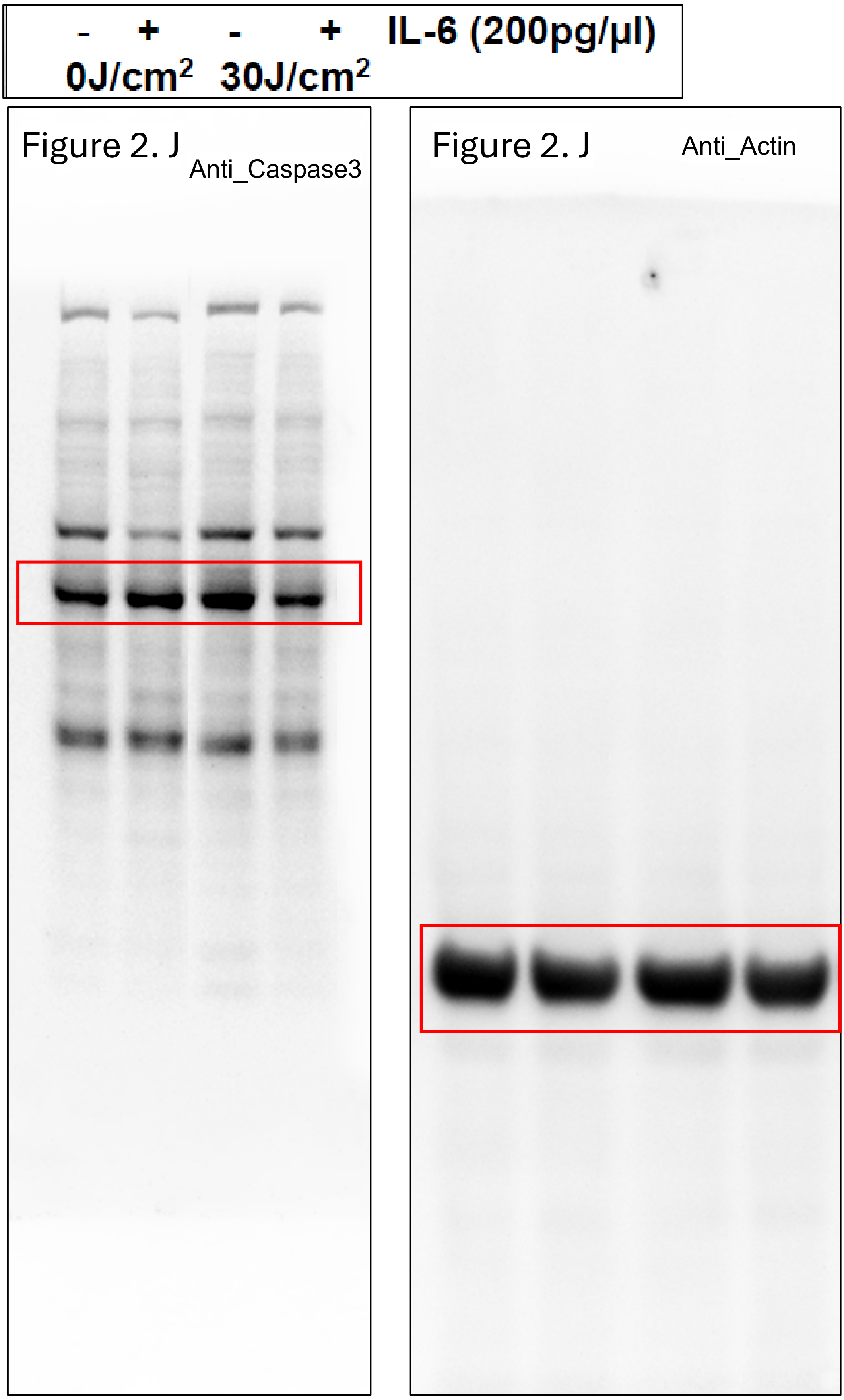
- + - + IL-6 (200pg/μl)
0J/cm2 30J/cm2
Figure 2. J
Anti_Caspase3
Figure 2. J
Anti_Actin

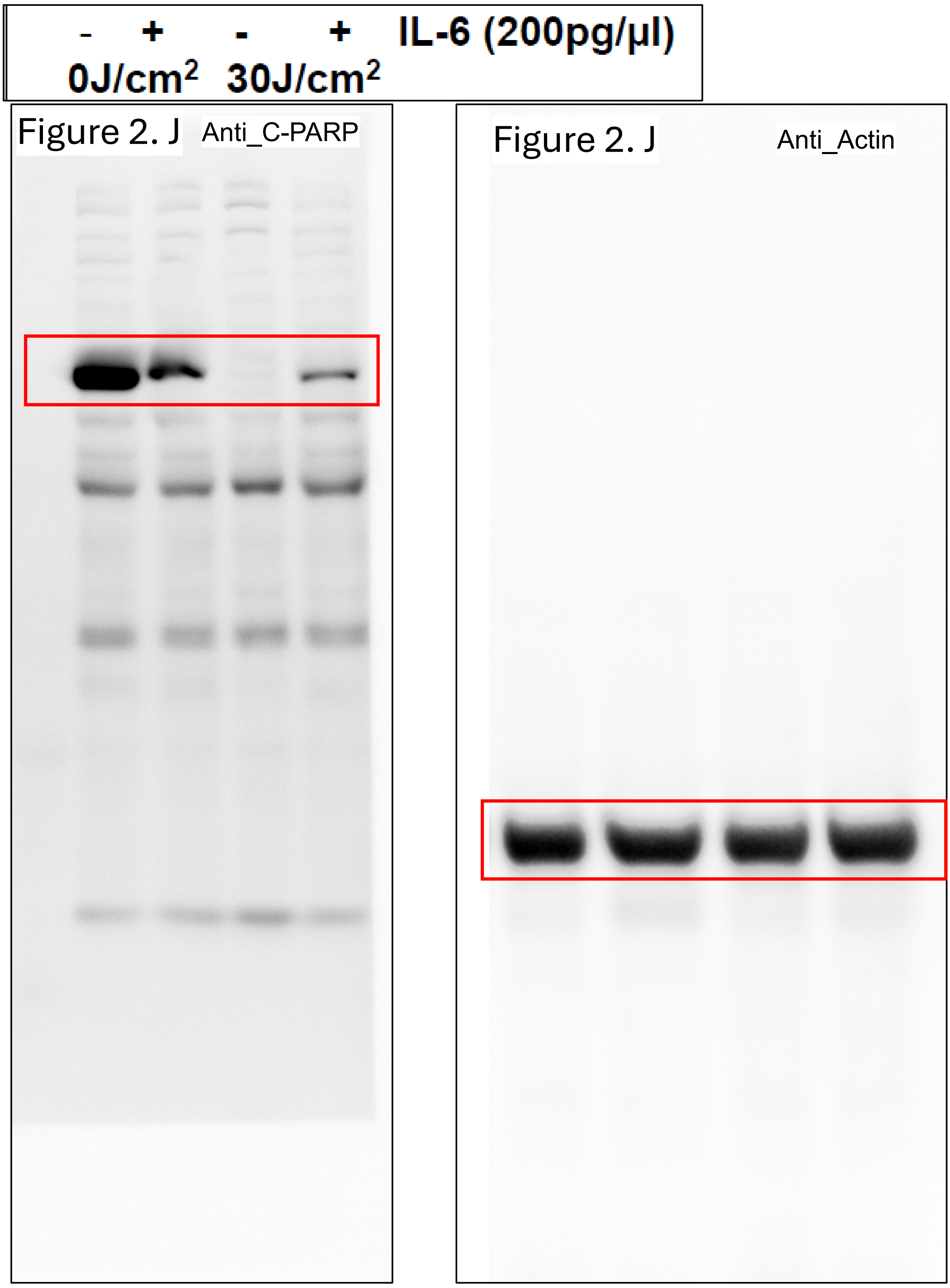
- + - + IL-6 (200pg/μl)
0J/cm2 30J/cm2
Figure 2. J
Anti_C-PARP
Figure 2. J
Anti_Actin

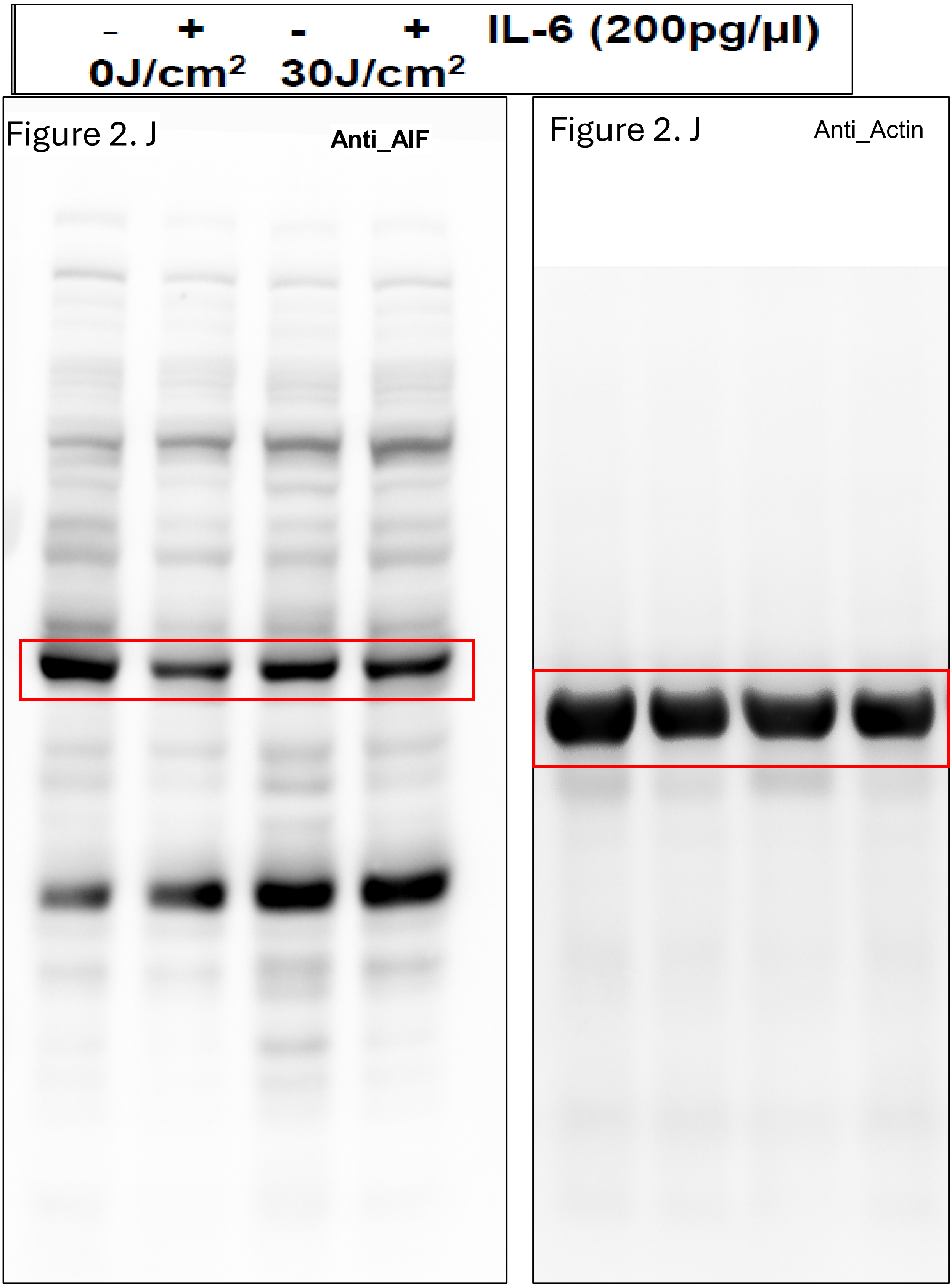
- + - + IL-6 (200pg/μl)
0J/cm2 30J/cm2
Figure 2. J
Anti_AIF
Figure 2. J
Anti_Actin

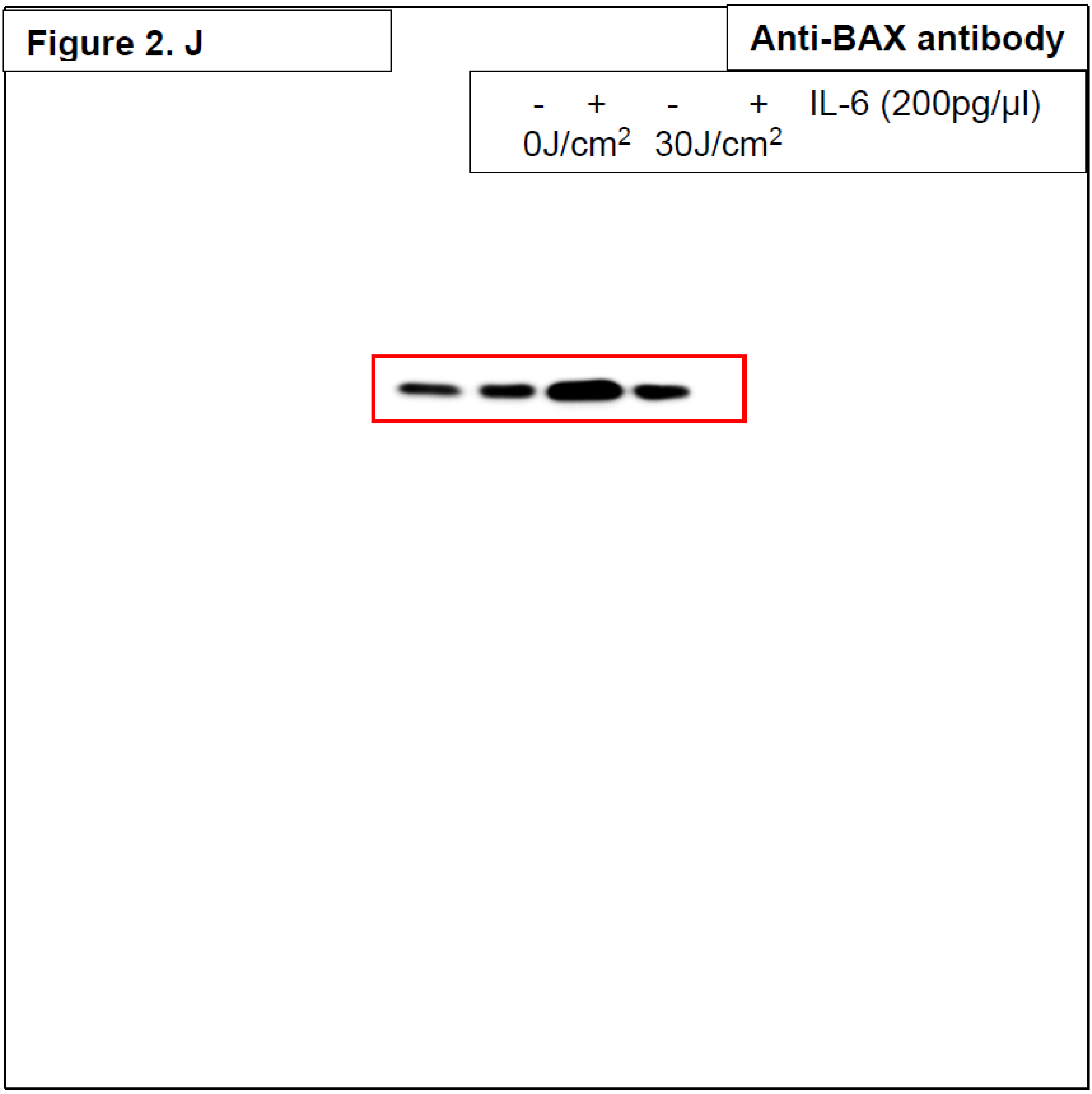
Figure 2. J
Anti-BAX antibody
- + - + IL-6 (200pg/μl)
0J/cm2 30J/cm2

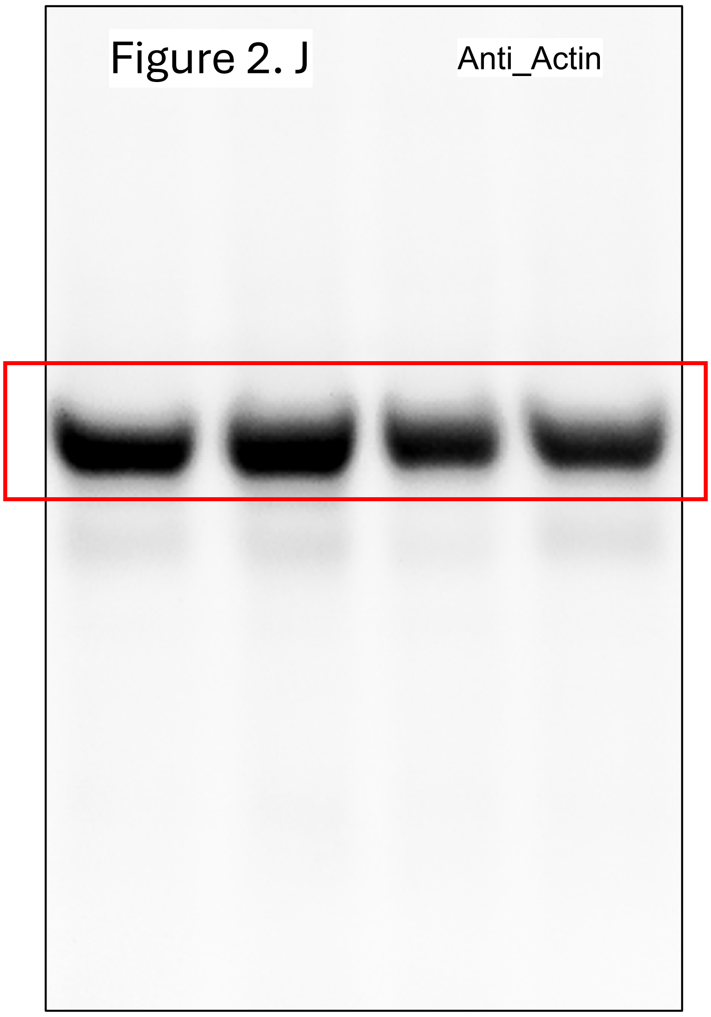
Figure 2. J
Anti_Actin

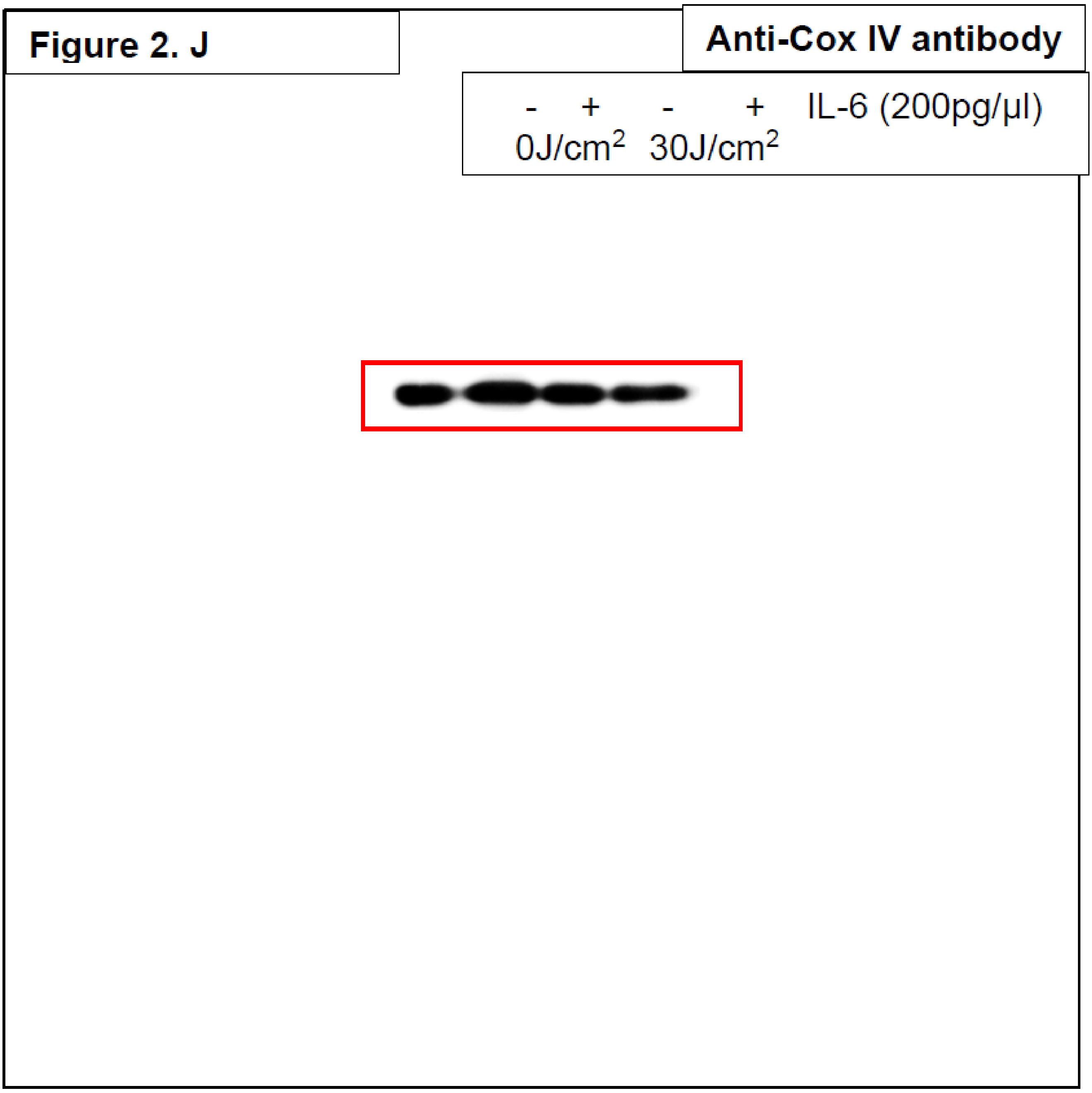
Figure 2. J
Anti-Cox IV antibody
- + - + IL-6 (200pg/μl)
0J/cm2 30J/cm2

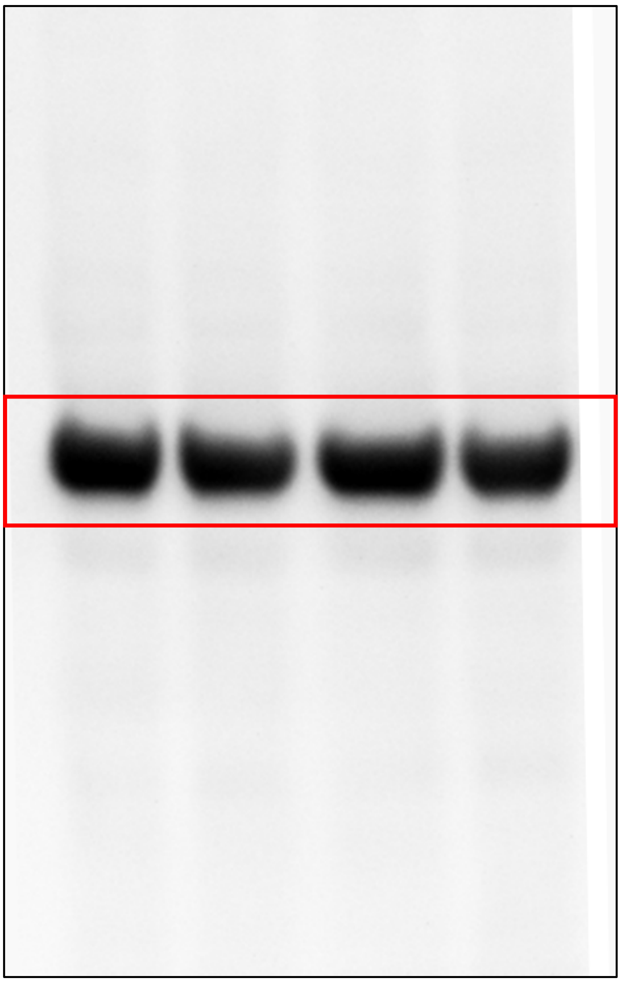

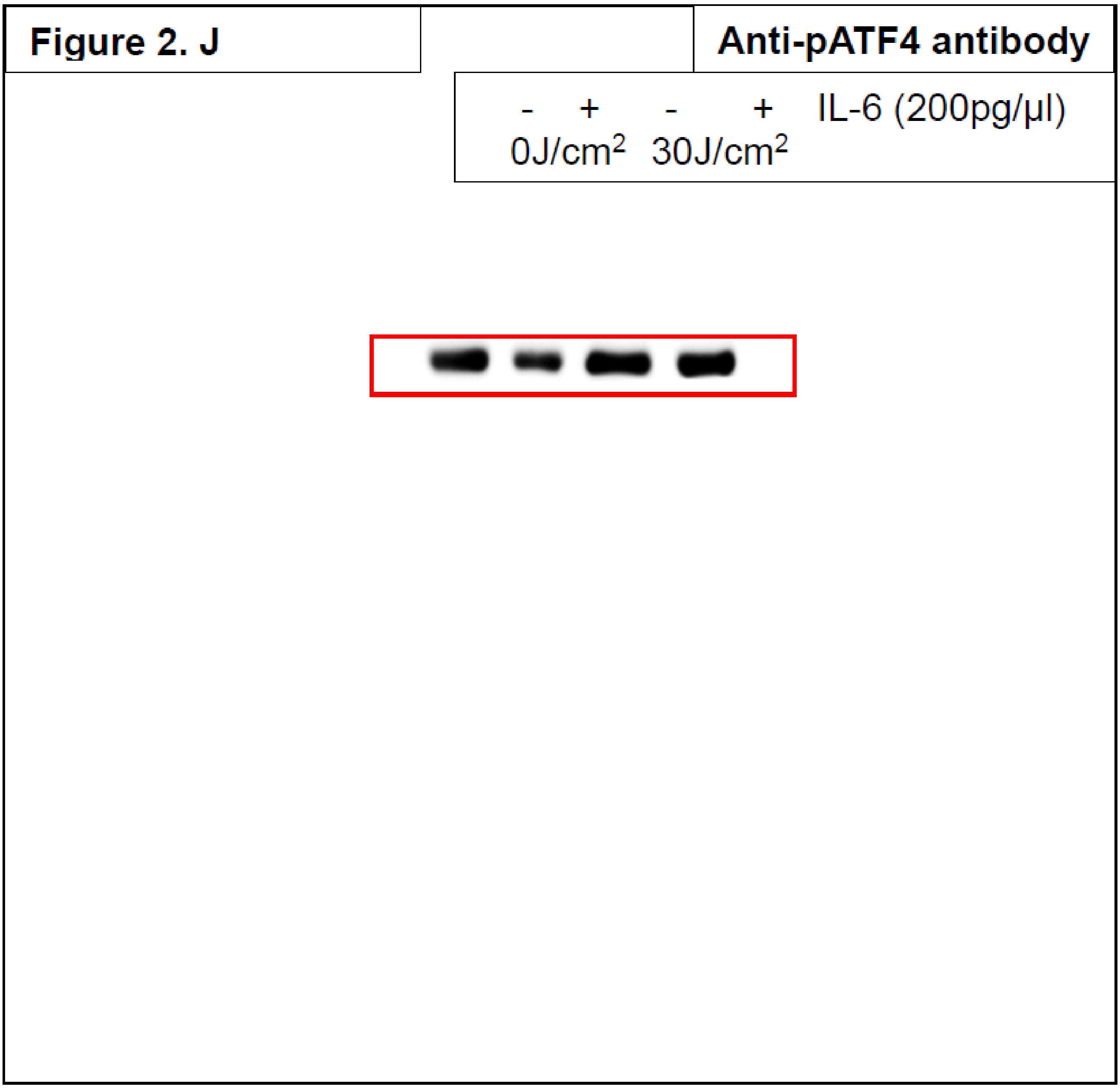
Figure 2. J
Anti-pATF4 antibody
- + - + IL-6 (200pg/μl)
0J/cm2 30J/cm2

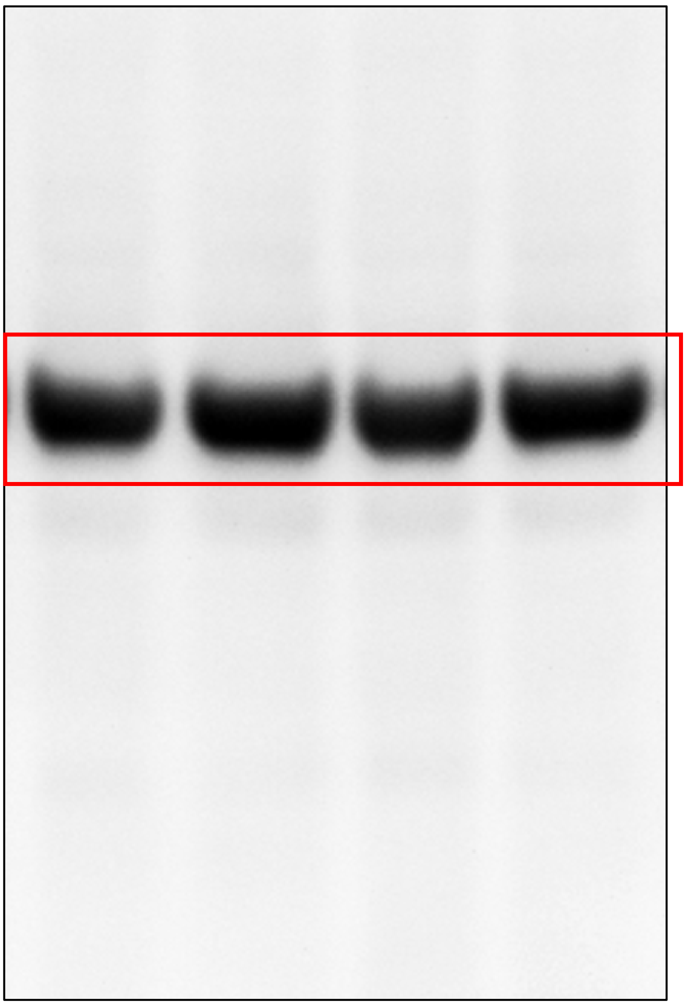

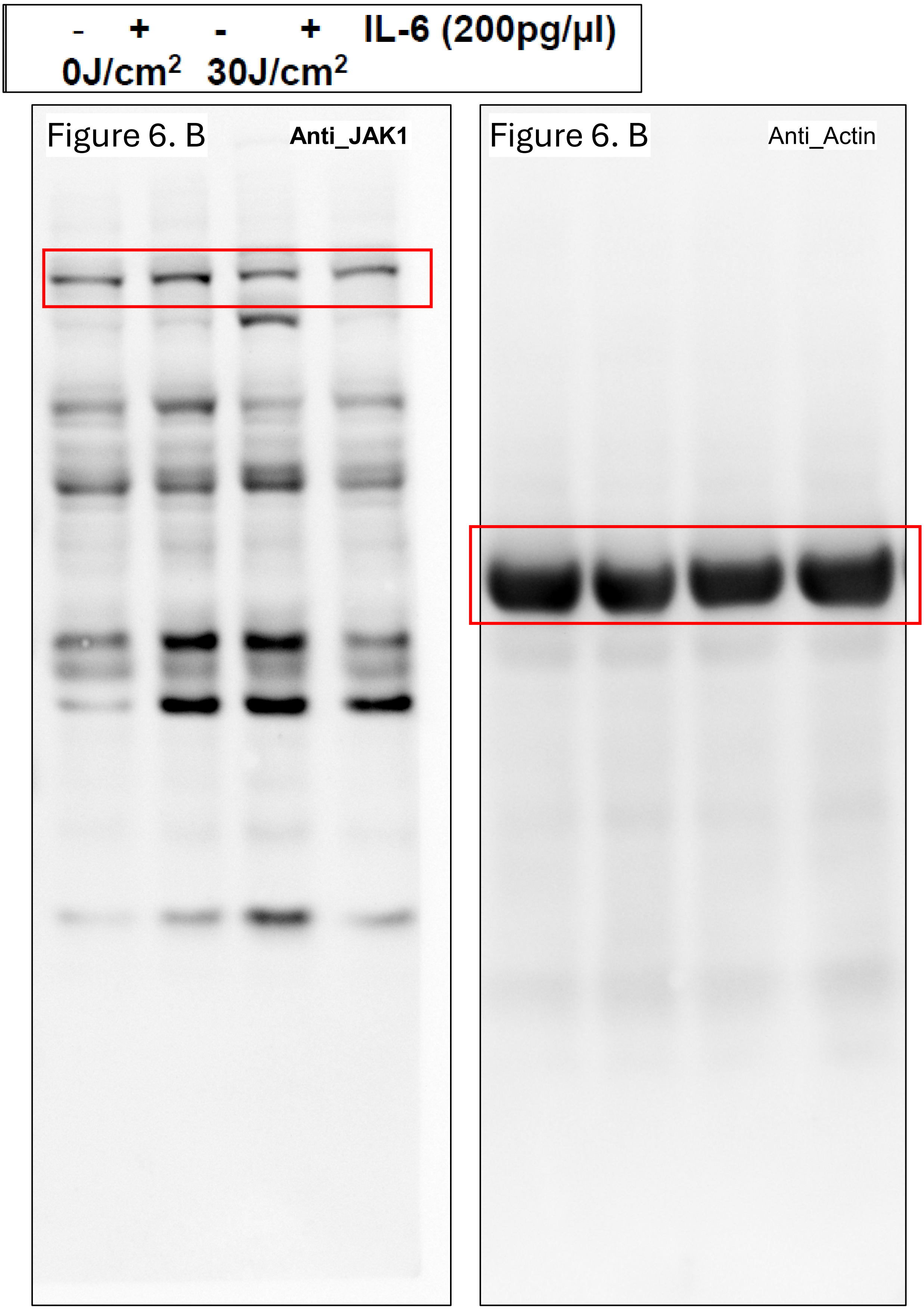
- + - + IL-6 (200pg/μl)
0J/cm2 30J/cm2
Figure 6. B
Anti_JAK1
Figure 6. B
Anti_Actin

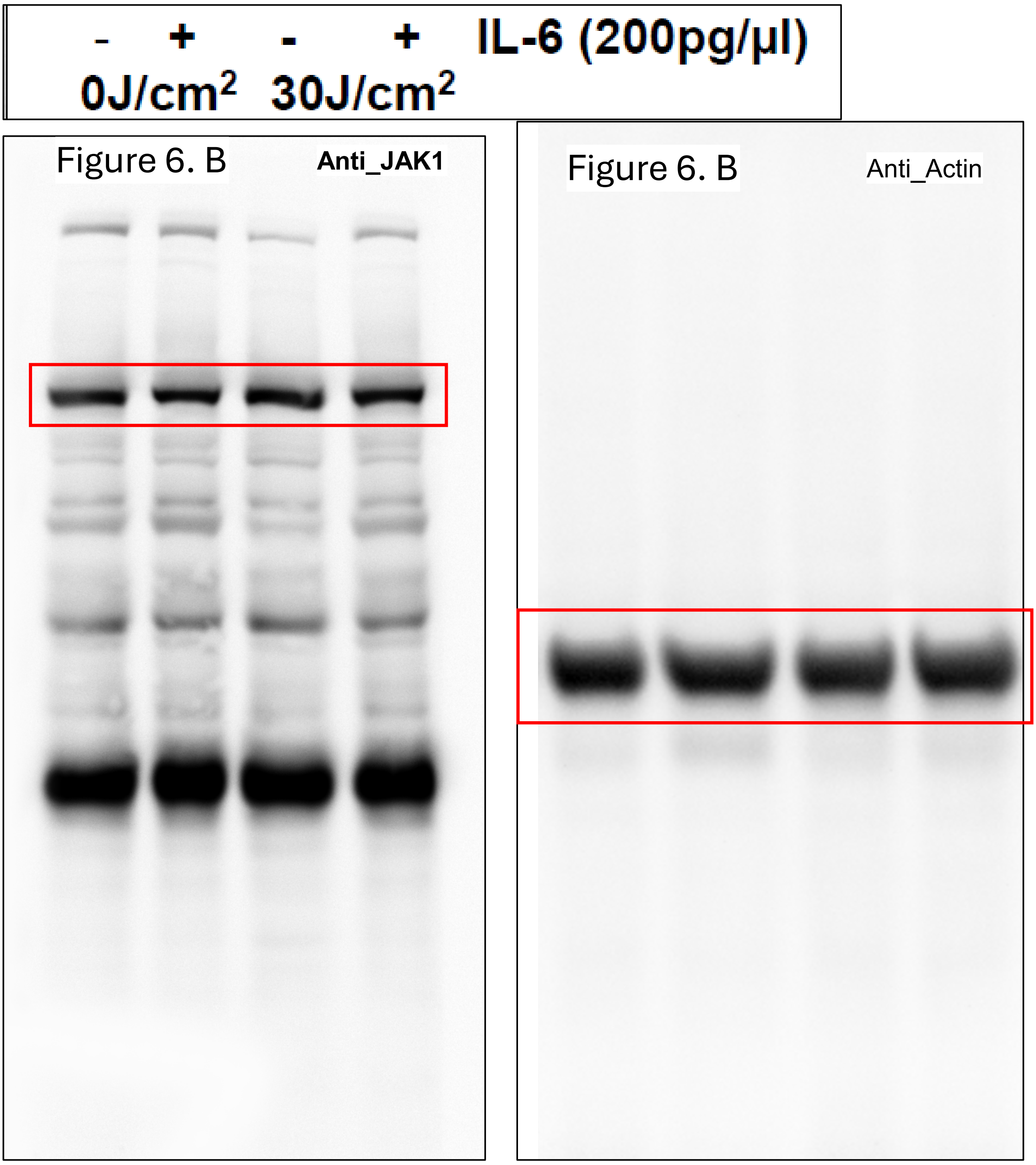
- + - + IL-6 (200pg/μl)
0J/cm2 30J/cm2
Figure 6. B
Anti_JAK1
Figure 6. B
Anti_Actin

Figure 6. B

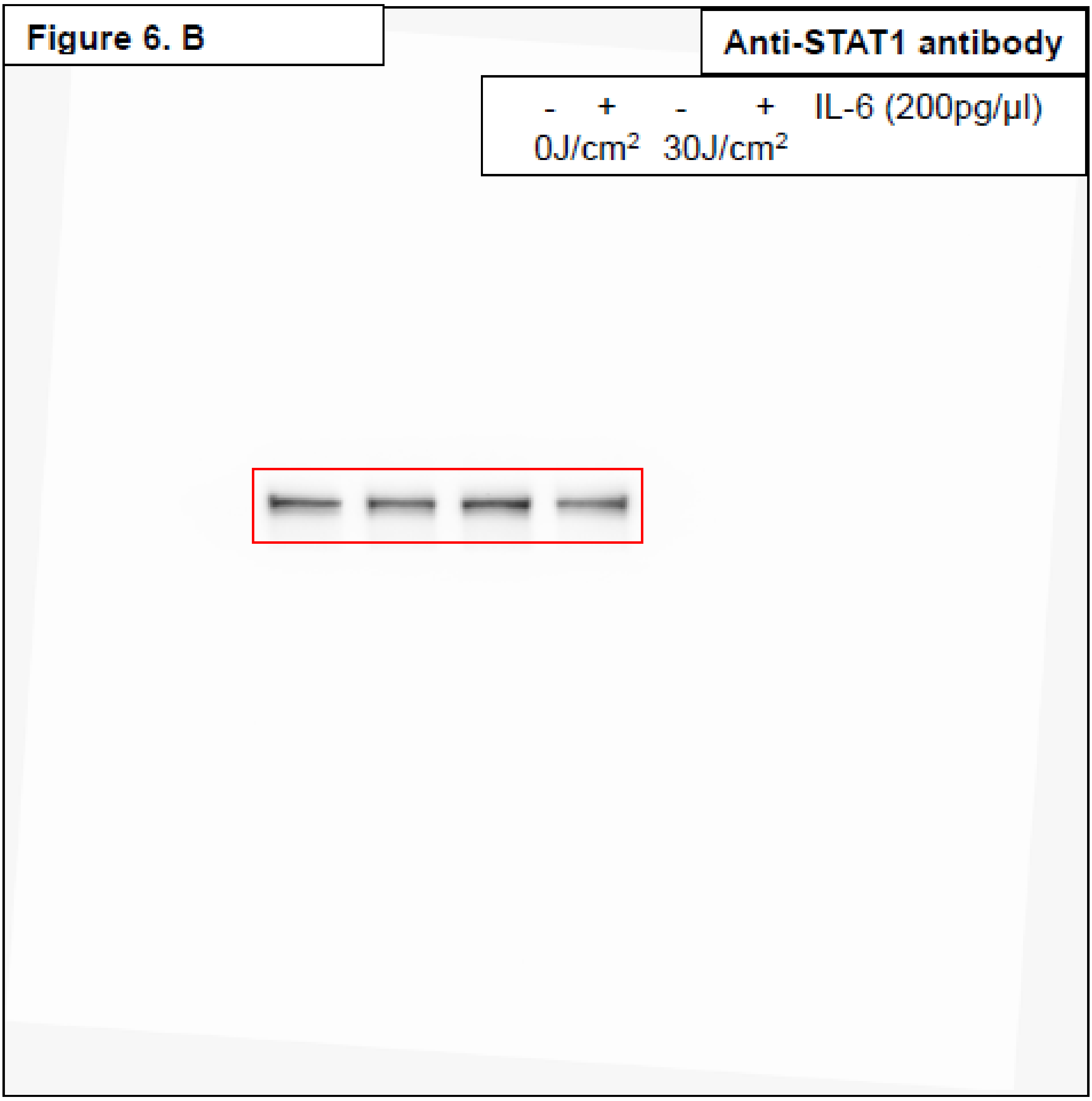

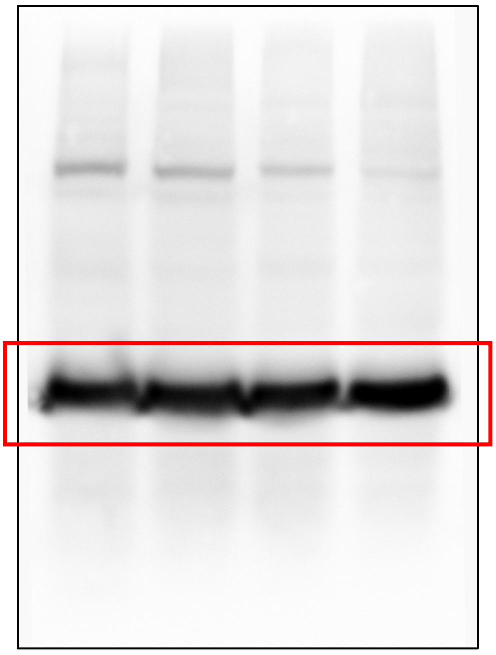

Figure 6. B

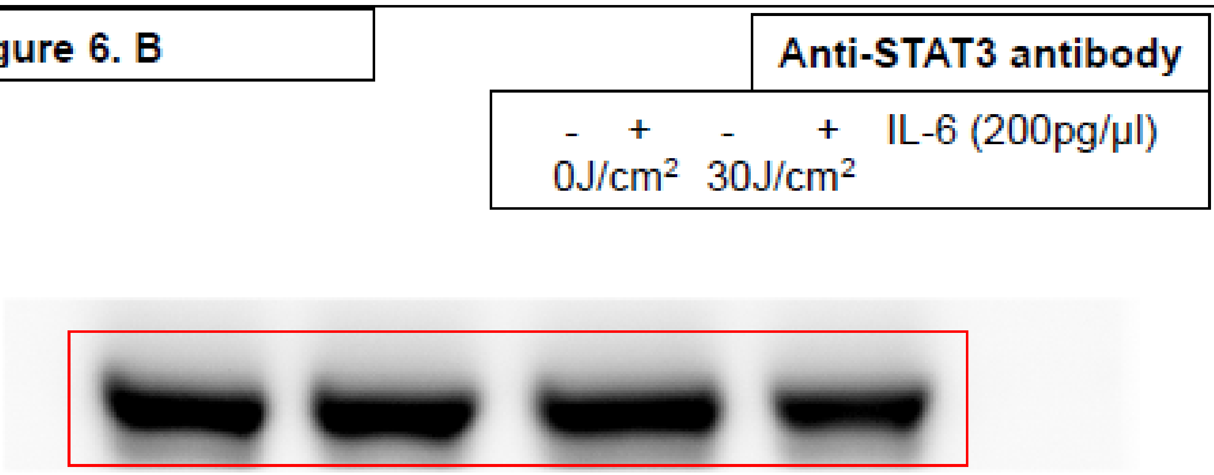

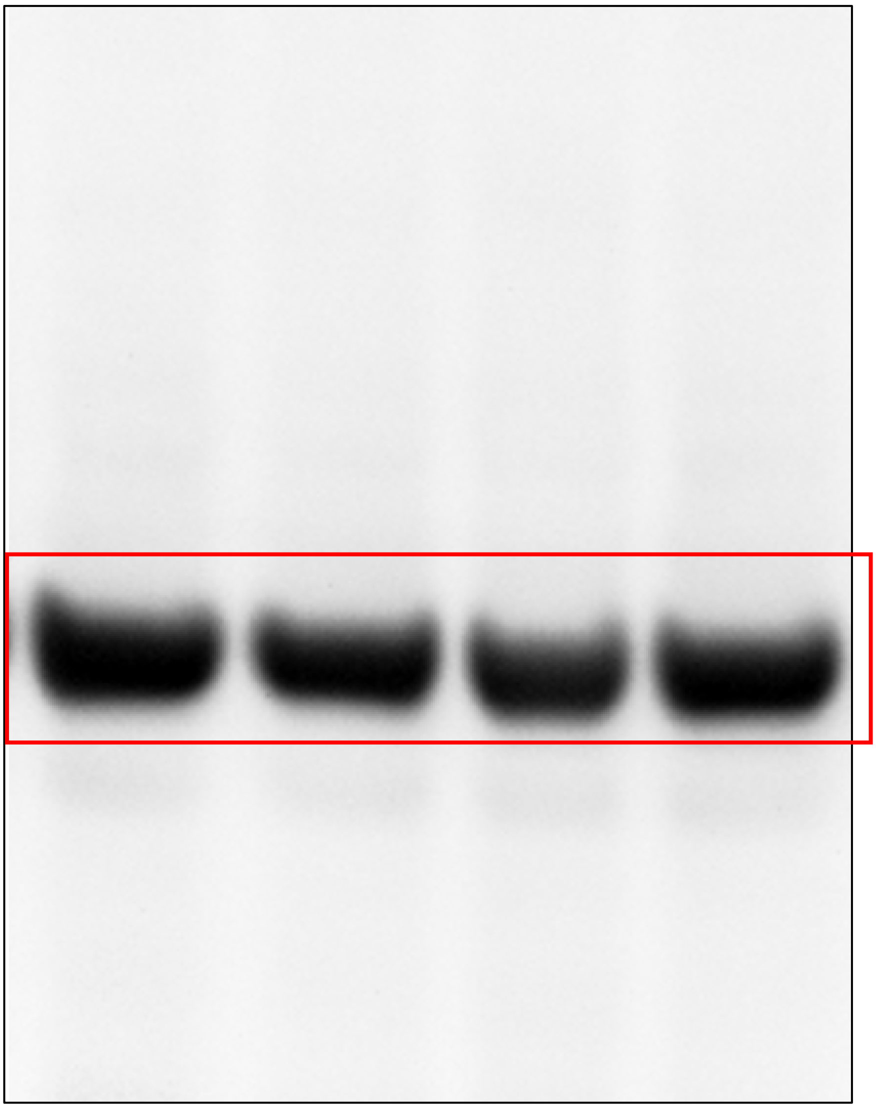

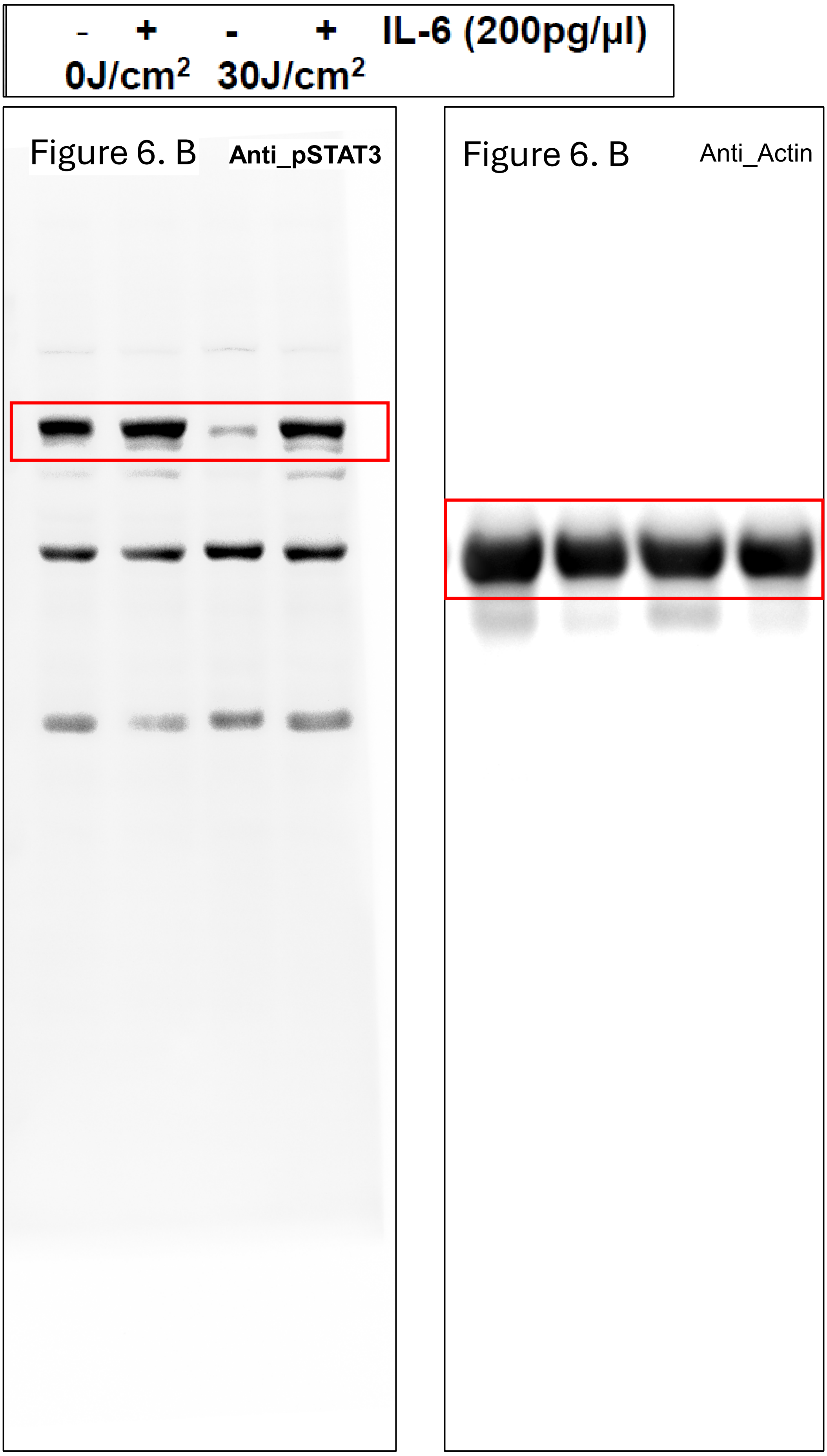

- + - + IL-6 (200pg/μl)
0J/cm2 30J/cm2
Figure 6. B
Anti_pSTAT3
Figure 6. B
Anti_Actin

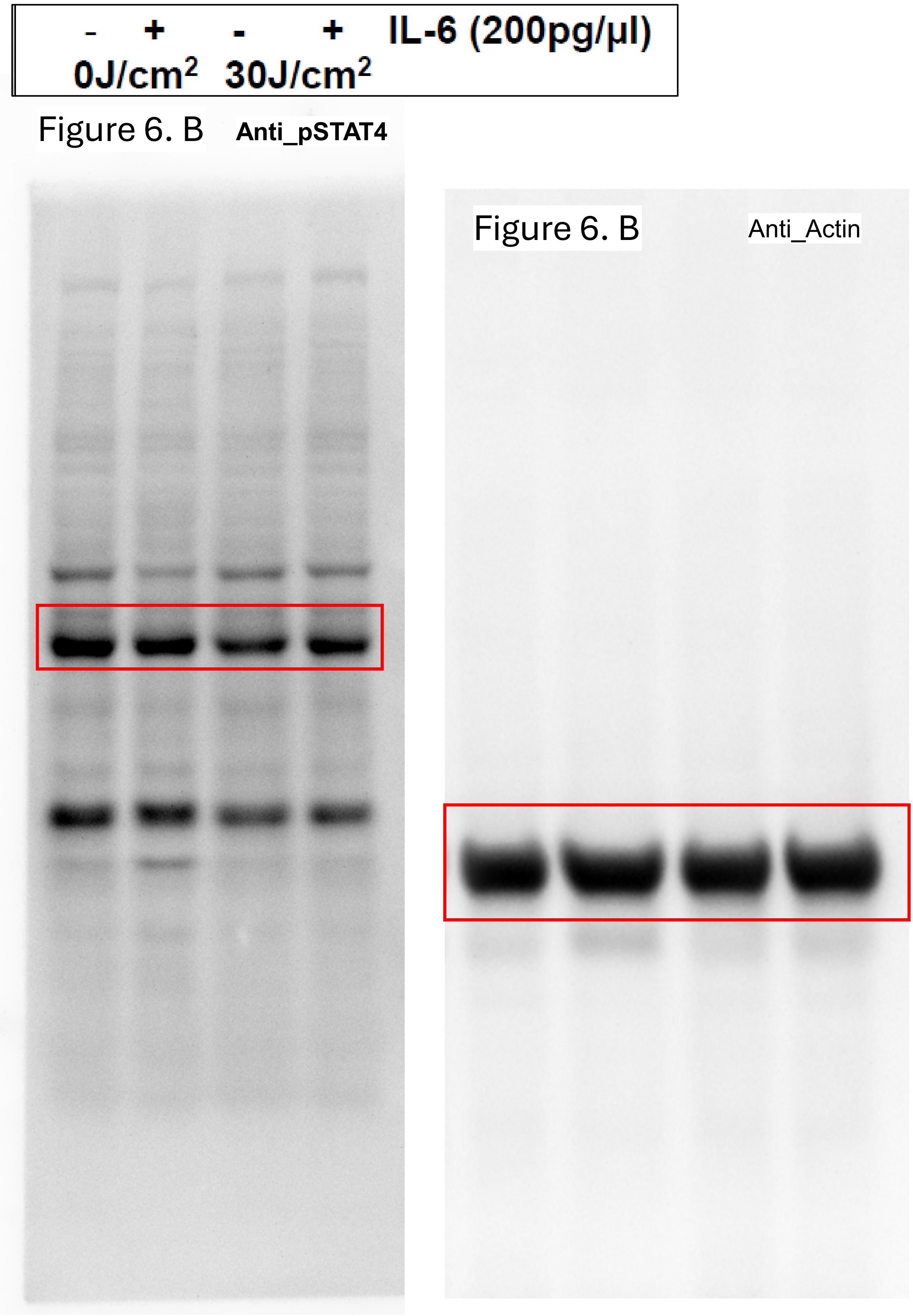
- + - + IL-6 (200pg/μl)
0J/cm2 30J/cm2
Figure 6. B
Anti_pSTAT4
Figure 6. B
Anti_Actin

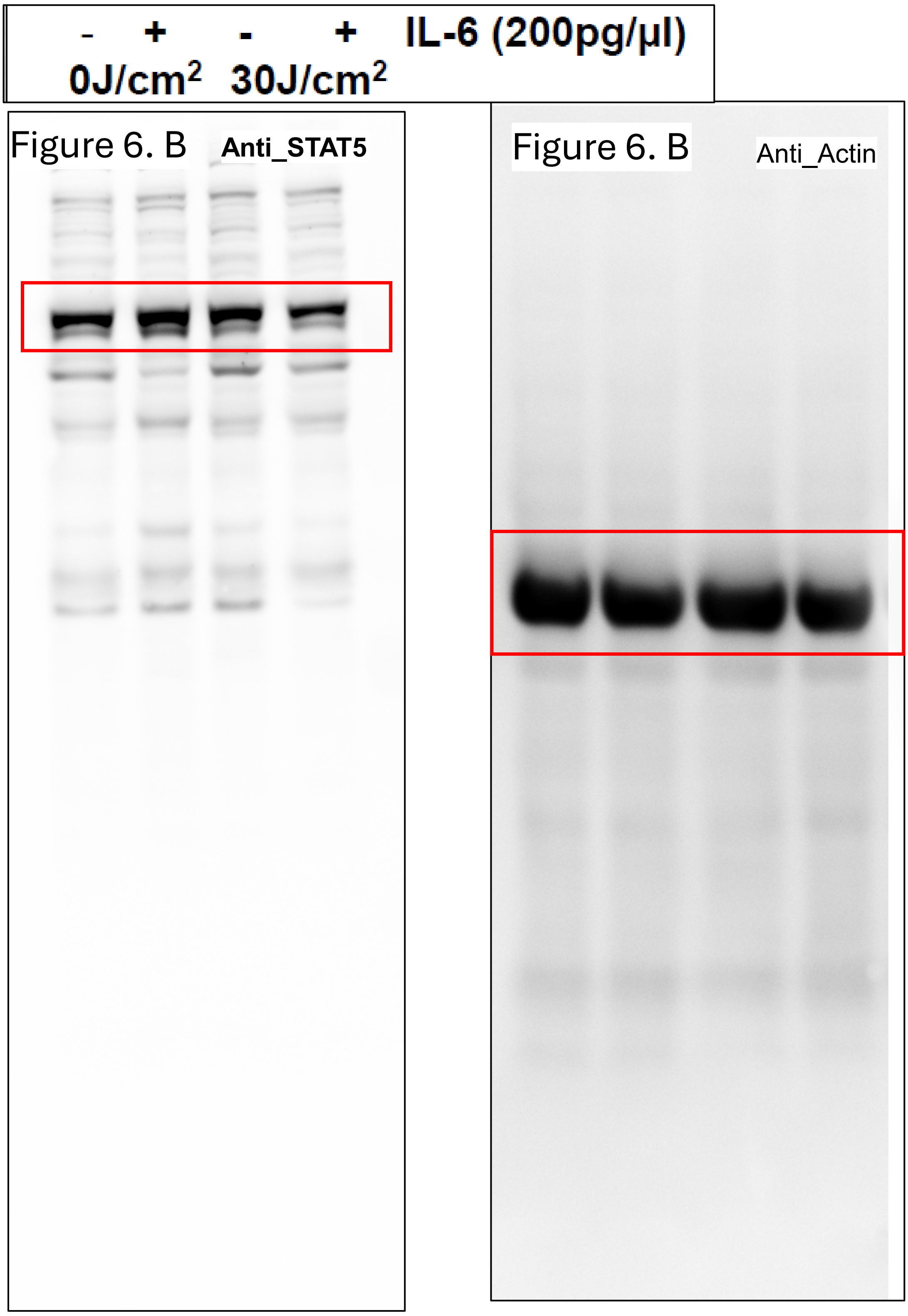
- + - + IL-6 (200pg/μl)
0J/cm2 30J/cm2
Figure 6. B
Anti_STAT5
Figure 6. B
Anti_Actin

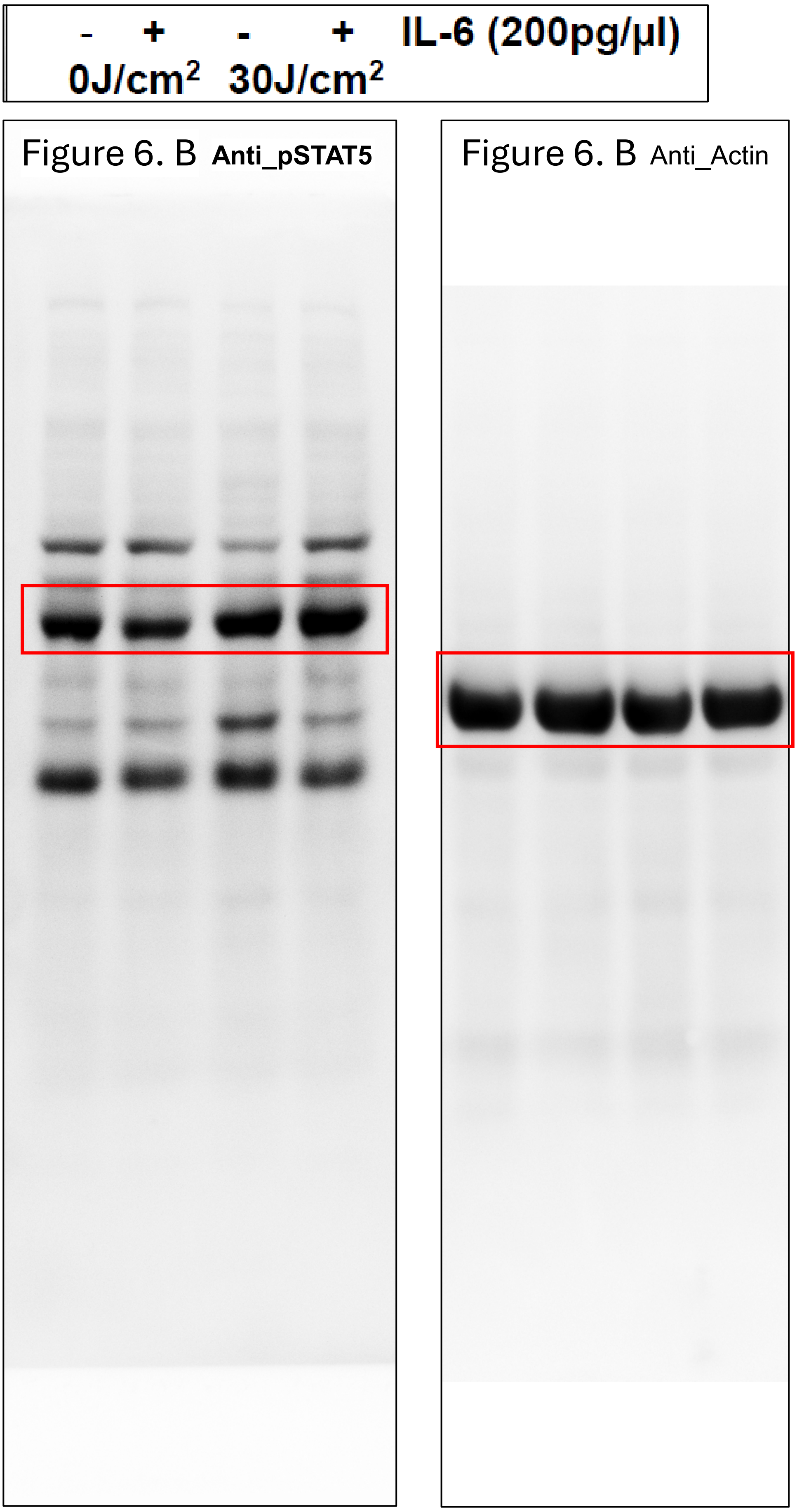
- + - + IL-6 (200pg/µl)
0J/cm2 30J/cm2
Figure 6. B Anti_pSTAT5
Figure 6. B Anti_Actin

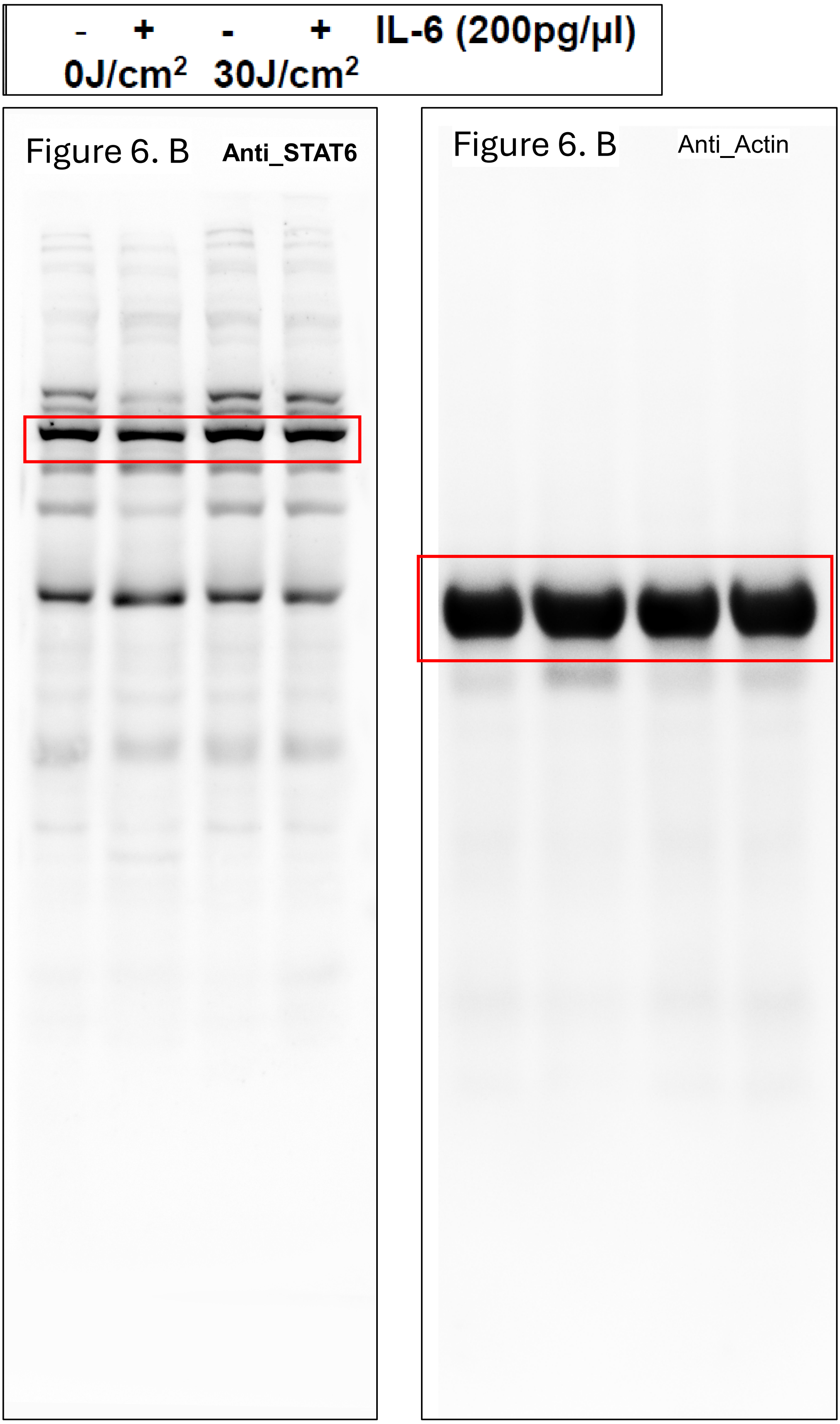

- + - + IL-6 (200pg/µl)
0J/cm2 30J/cm2
Figure 6. B
Anti_STAT6
Figure 6. B
Anti_Actin

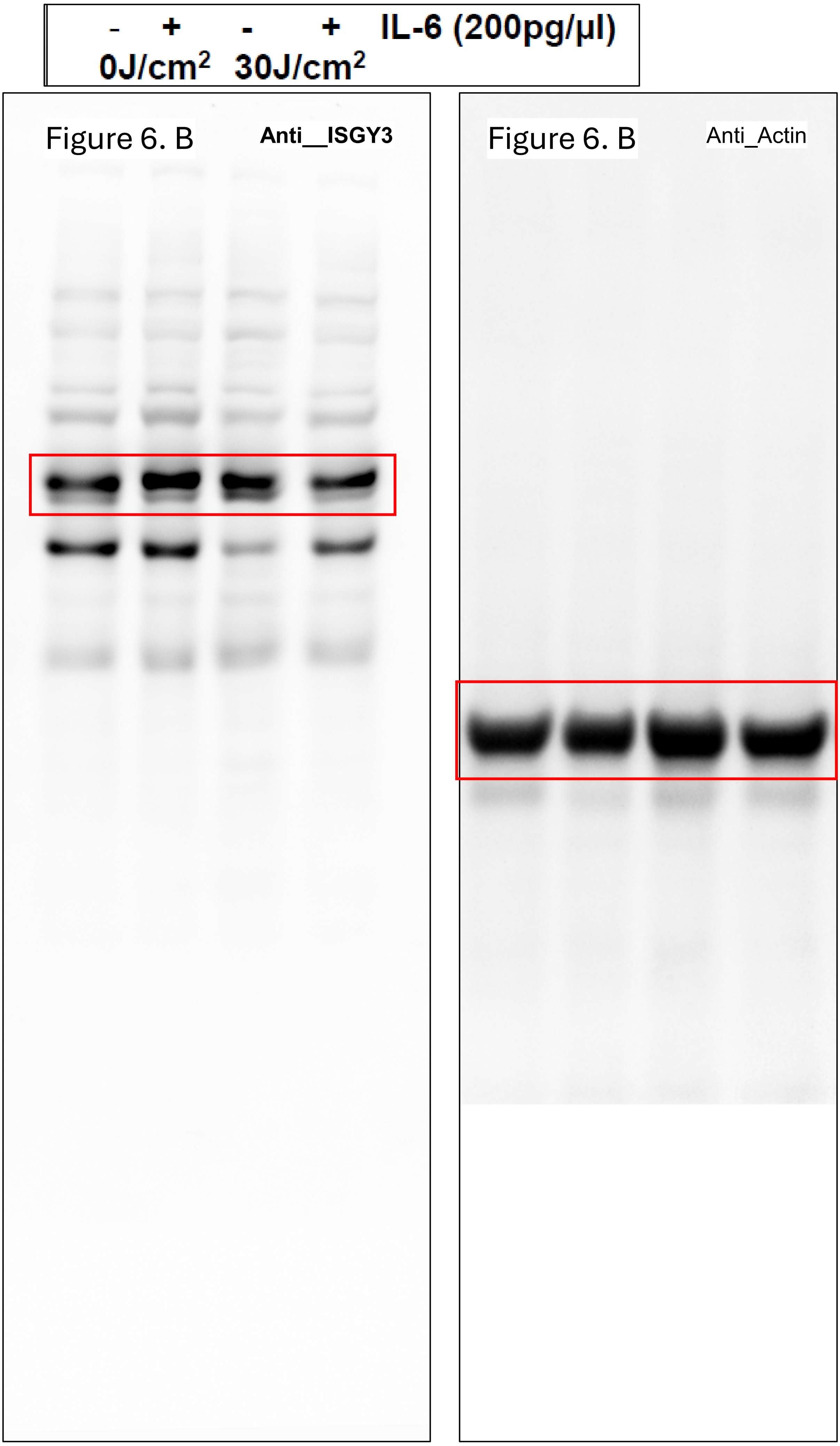
- + - + IL-6 (200pg/μl)
0J/cm2 30J/cm2
Figure 6. B
Anti__ISGY3
Figure 6. B
Anti_Actin

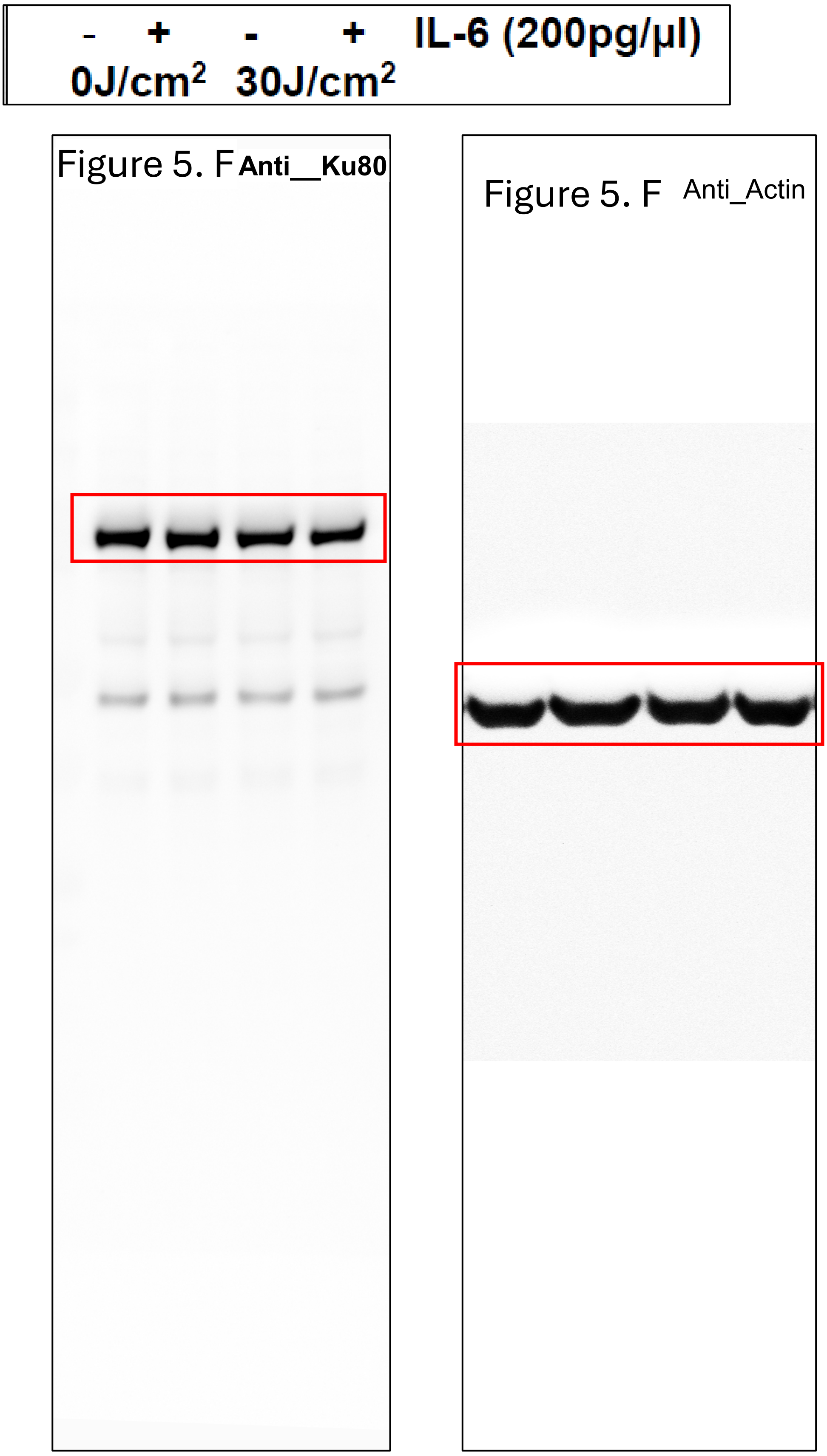
- + - + IL-6 (200pg/μl)
0J/cm2 30J/cm2
Figure 5. F Anti__Ku80
Figure 5. F Anti_Actin

Figure 5. F

Anti-MDM2 antibody

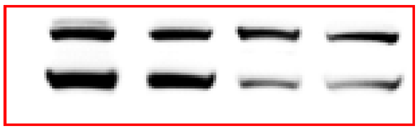

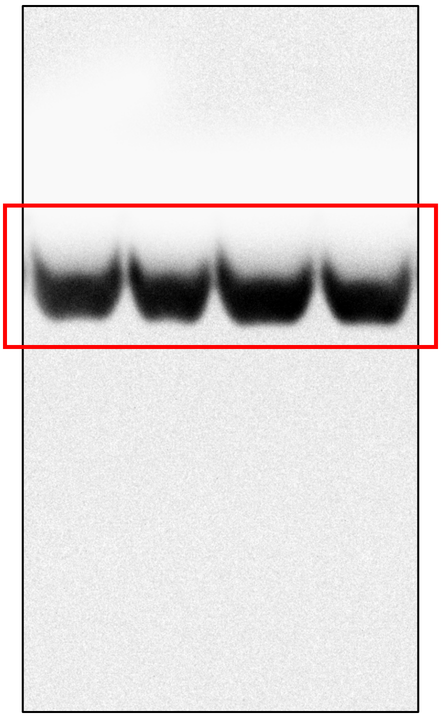

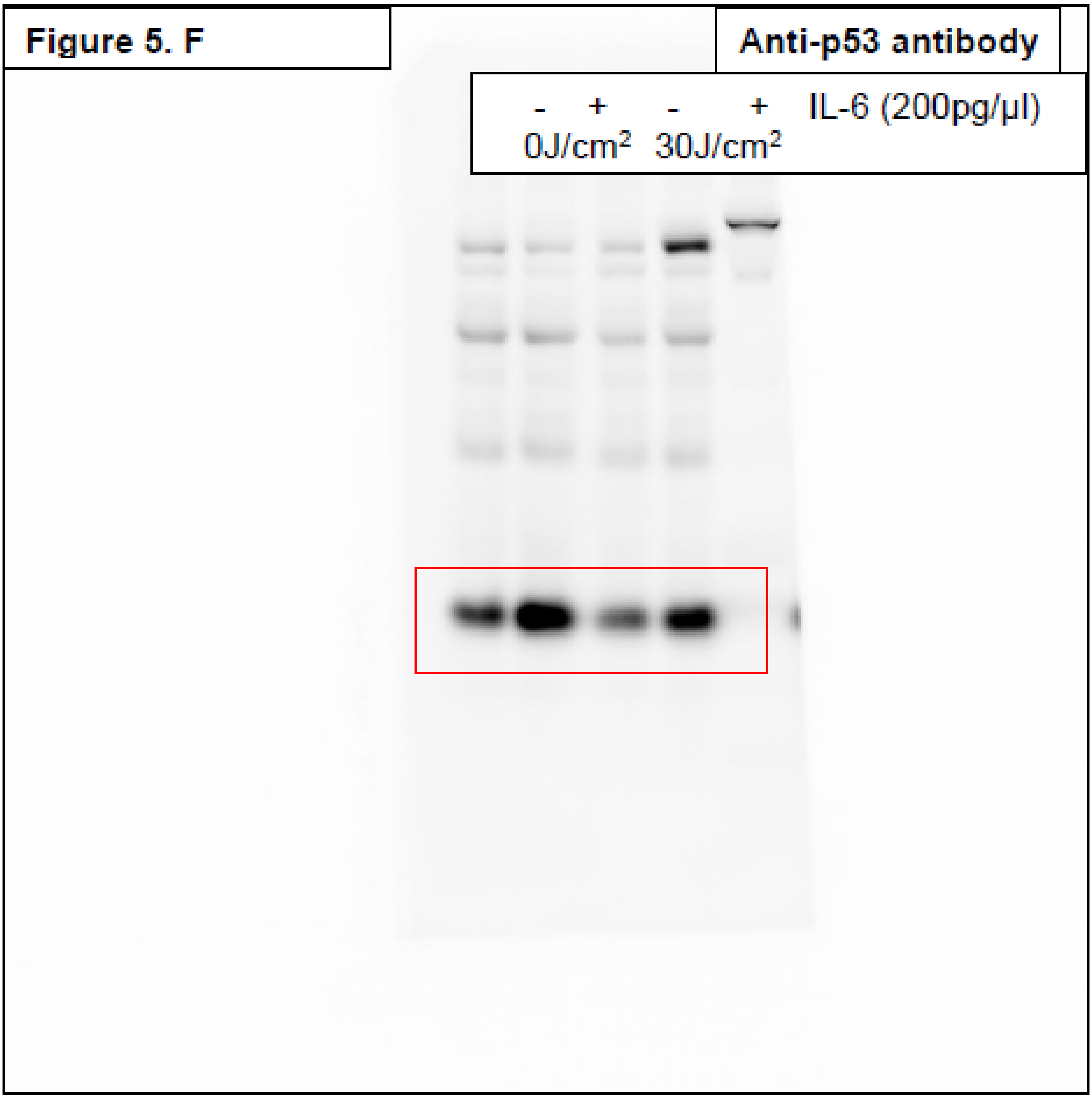
Figure 5. F
Anti-p53 antibody
- + - + IL-6 (200pg/μl)
0J/cm2 30J/cm2

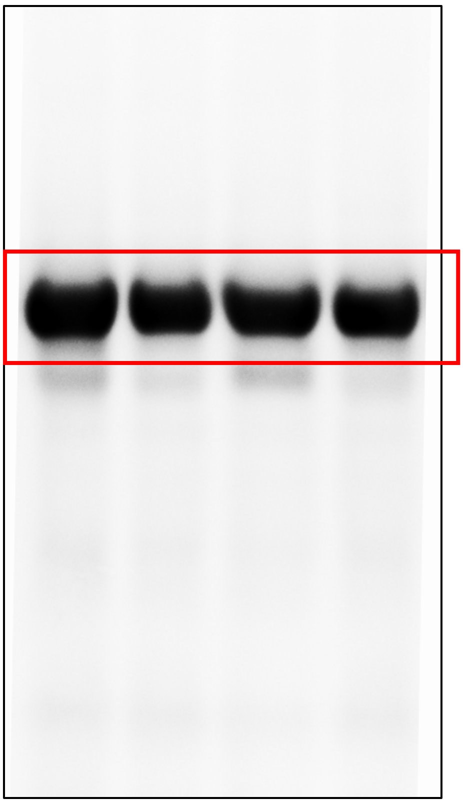

Figure 5. F

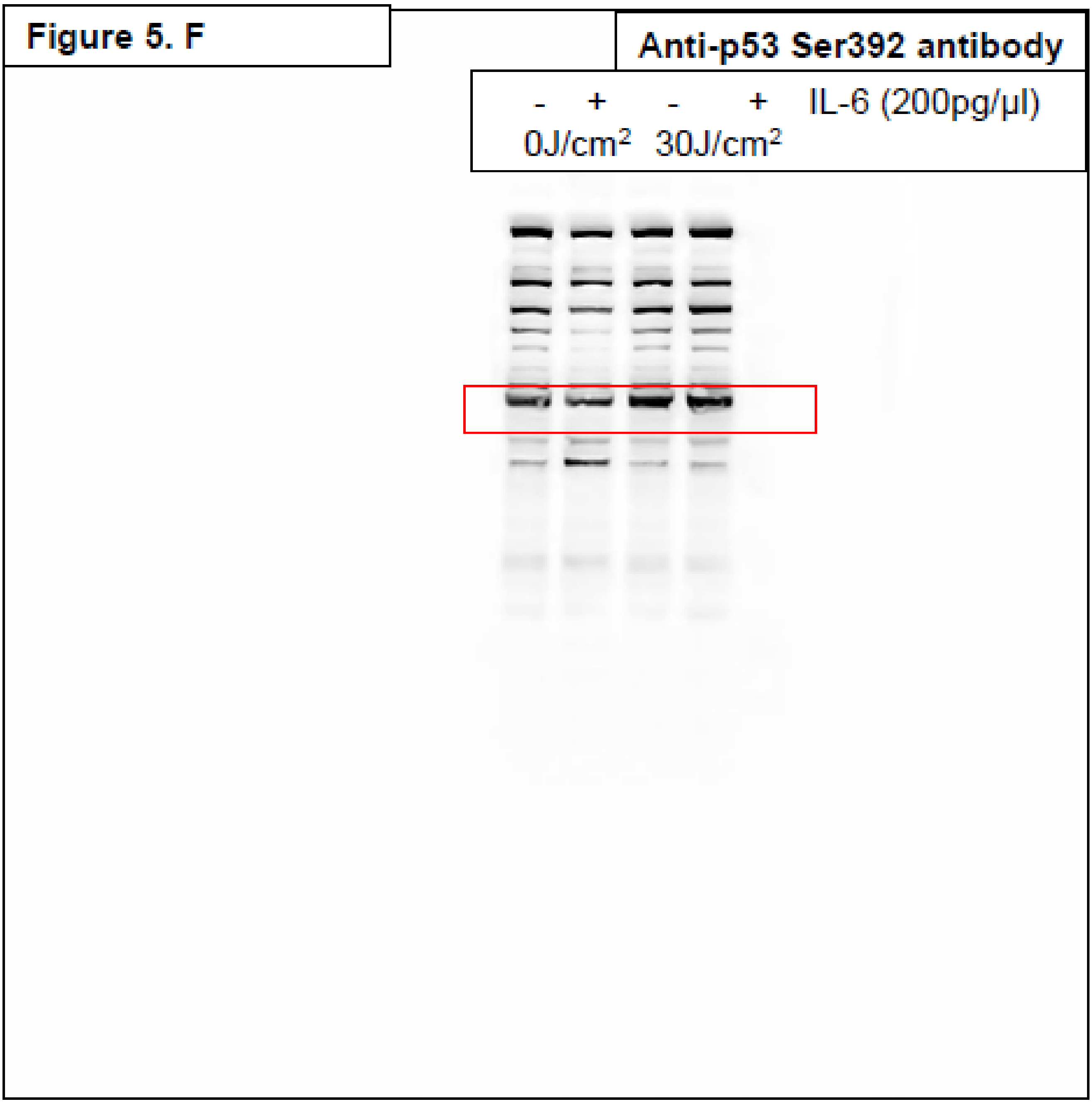

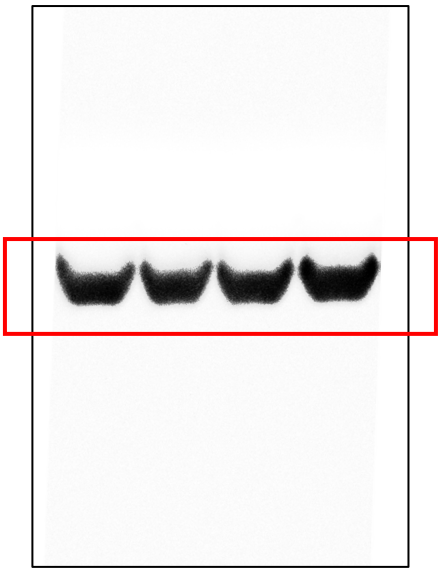

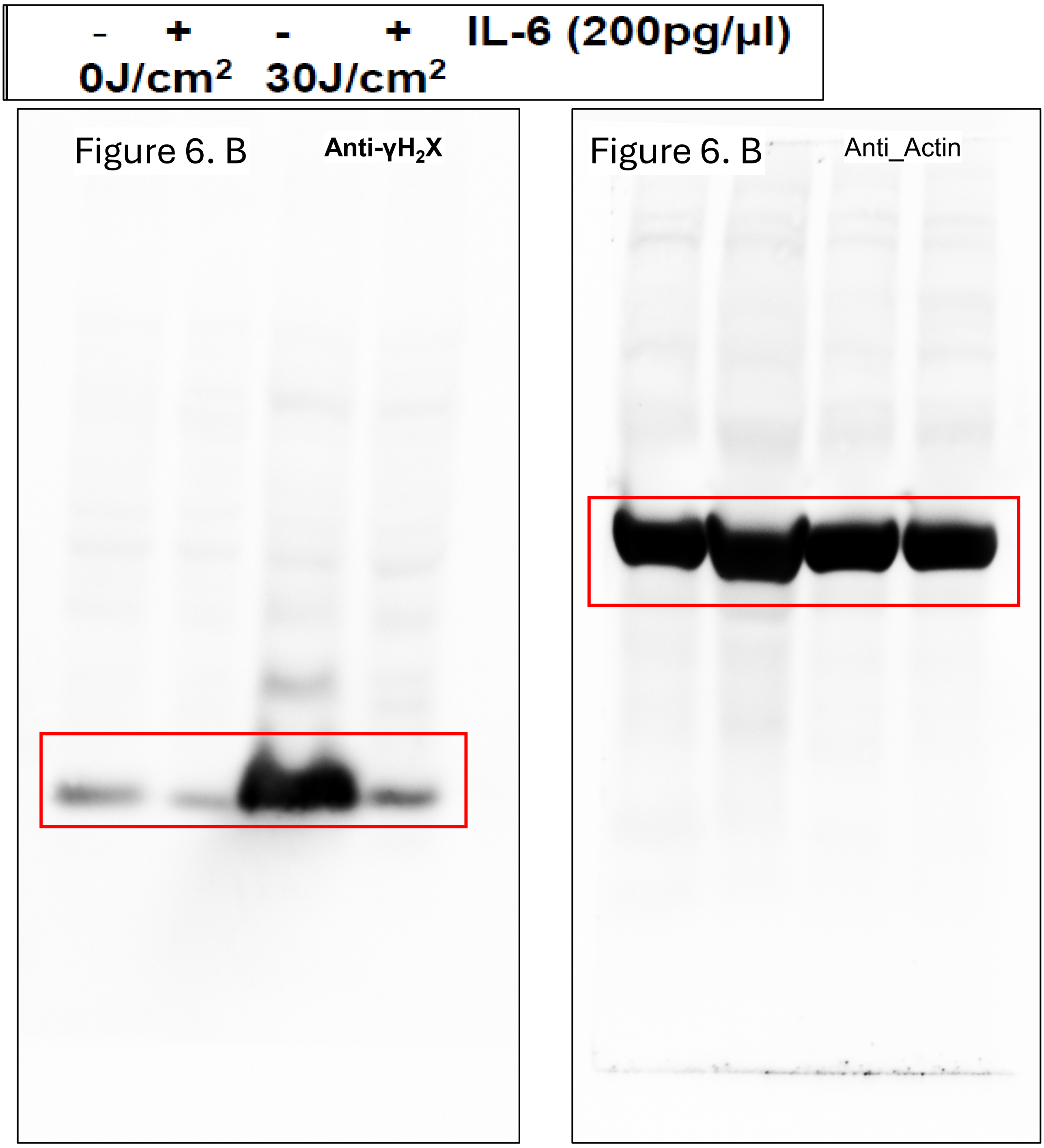

Figure 6. B

Figure 6. B

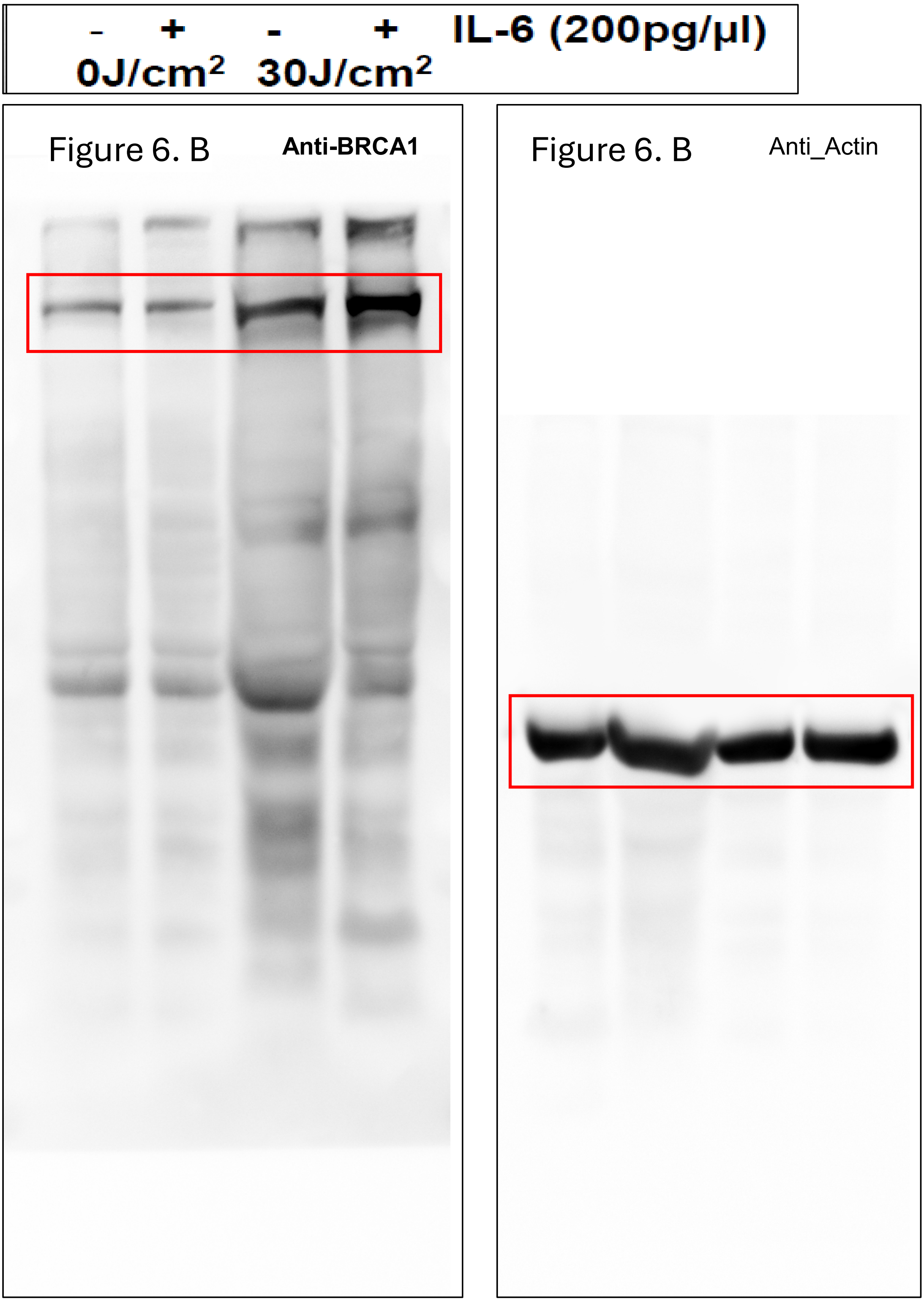
- + - + IL-6 (200pg/µl)
0J/cm2 30J/cm2
Figure 6. B
Anti-BRCA1
Figure 6. B
Anti_Actin

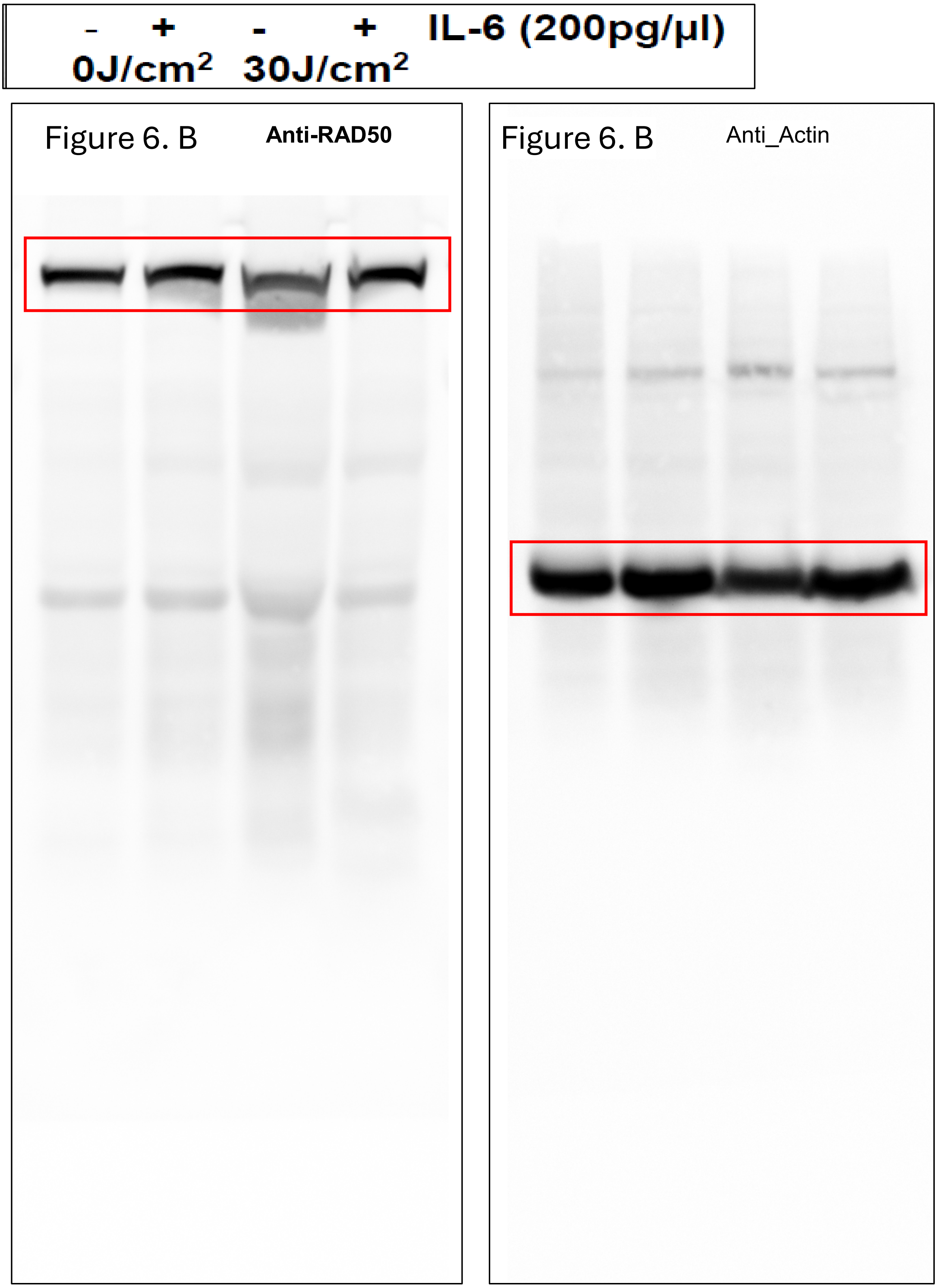
- + - + IL-6 (200pg/μl)
0J/cm2 30J/cm2
Figure 6. B
Anti-RAD50
Figure 6. B
Anti_Actin

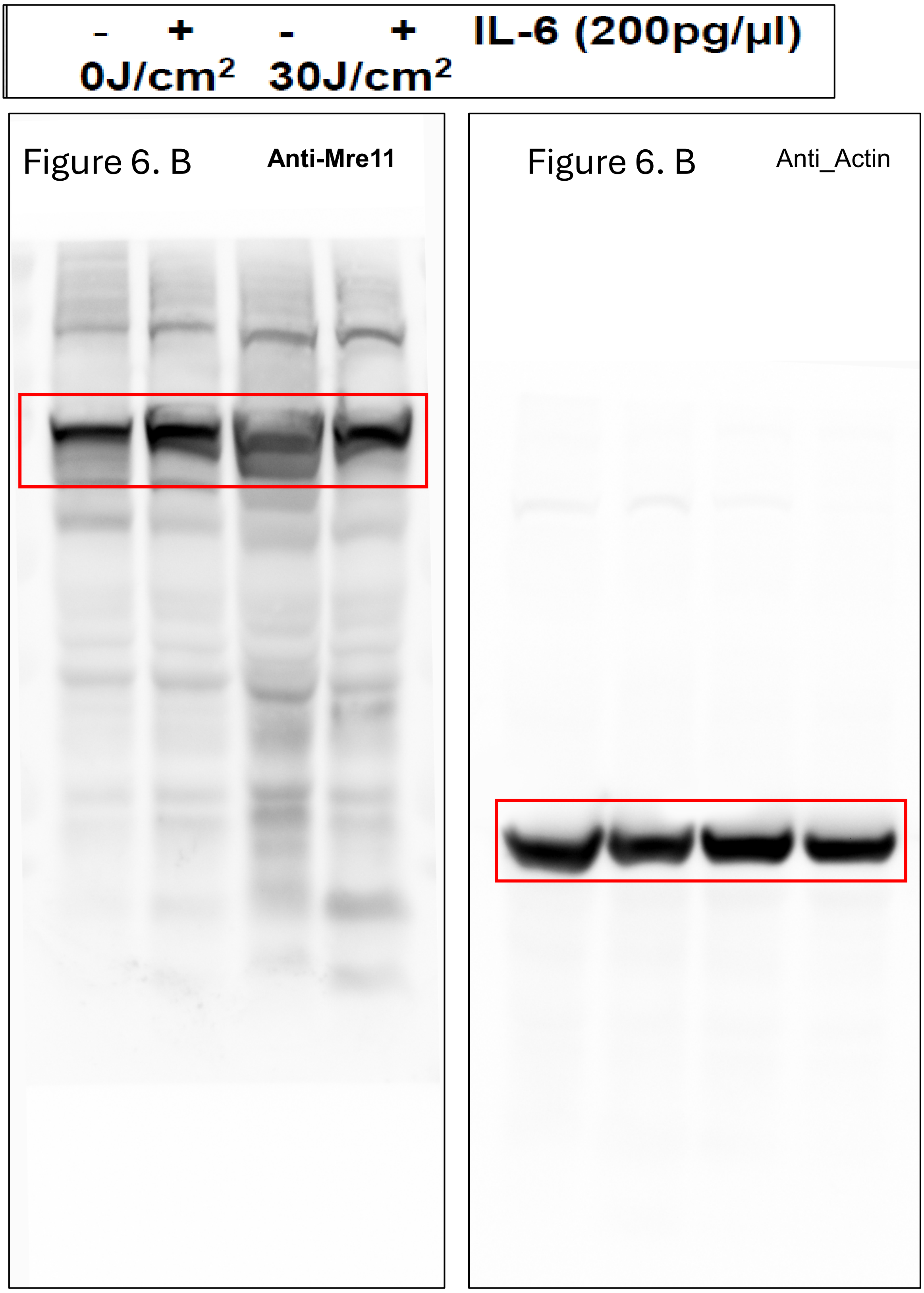
- + - + IL-6 (200pg/μl)
0J/cm2 30J/cm2
Figure 6. B
Anti-Mre11
Figure 6. B
Anti_Actin

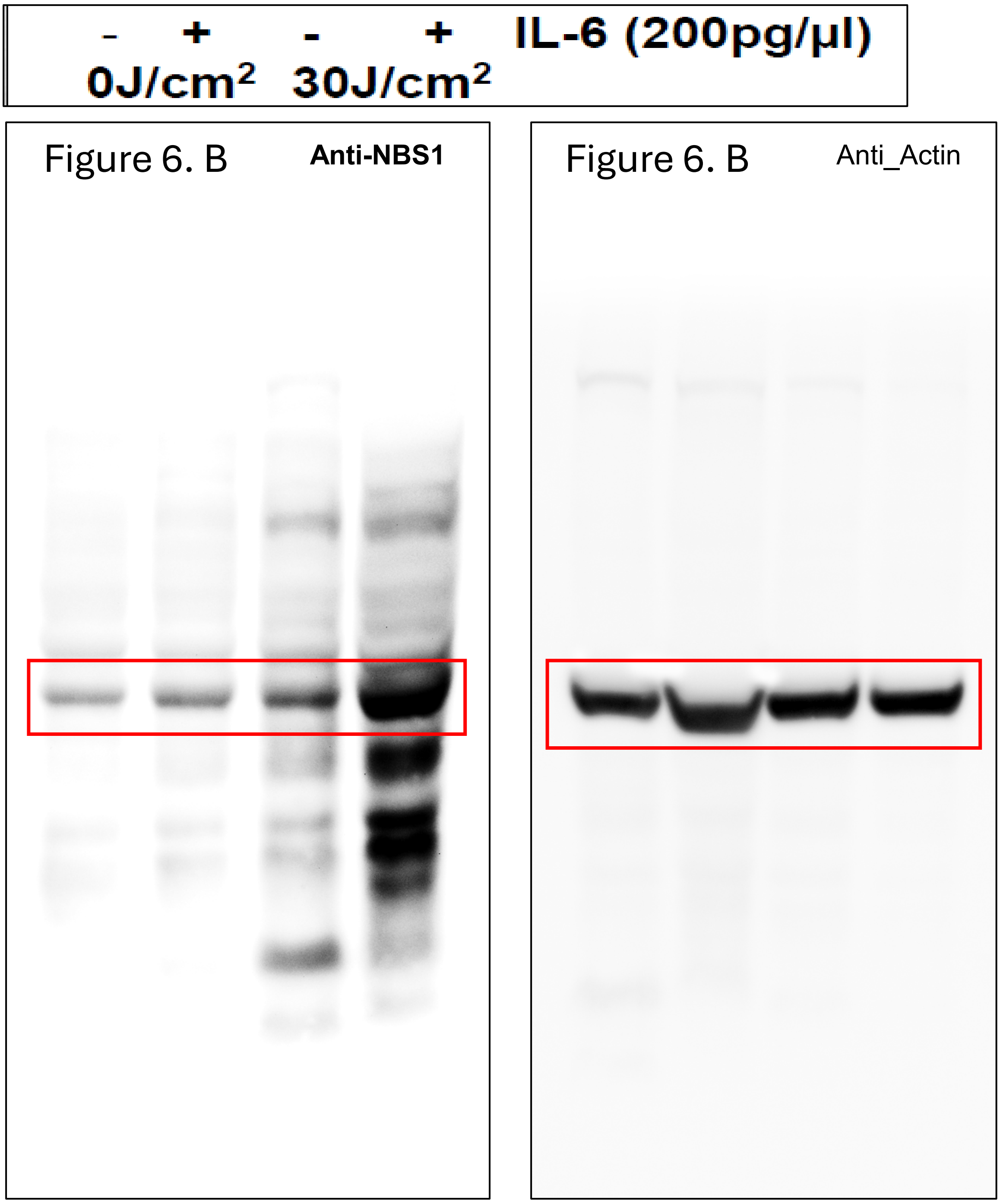
- + - + IL-6 (200pg/µl)
0J/cm2 30J/cm2
Figure 6. B
Anti-NBS1
Figure 6. B
Anti_Actin

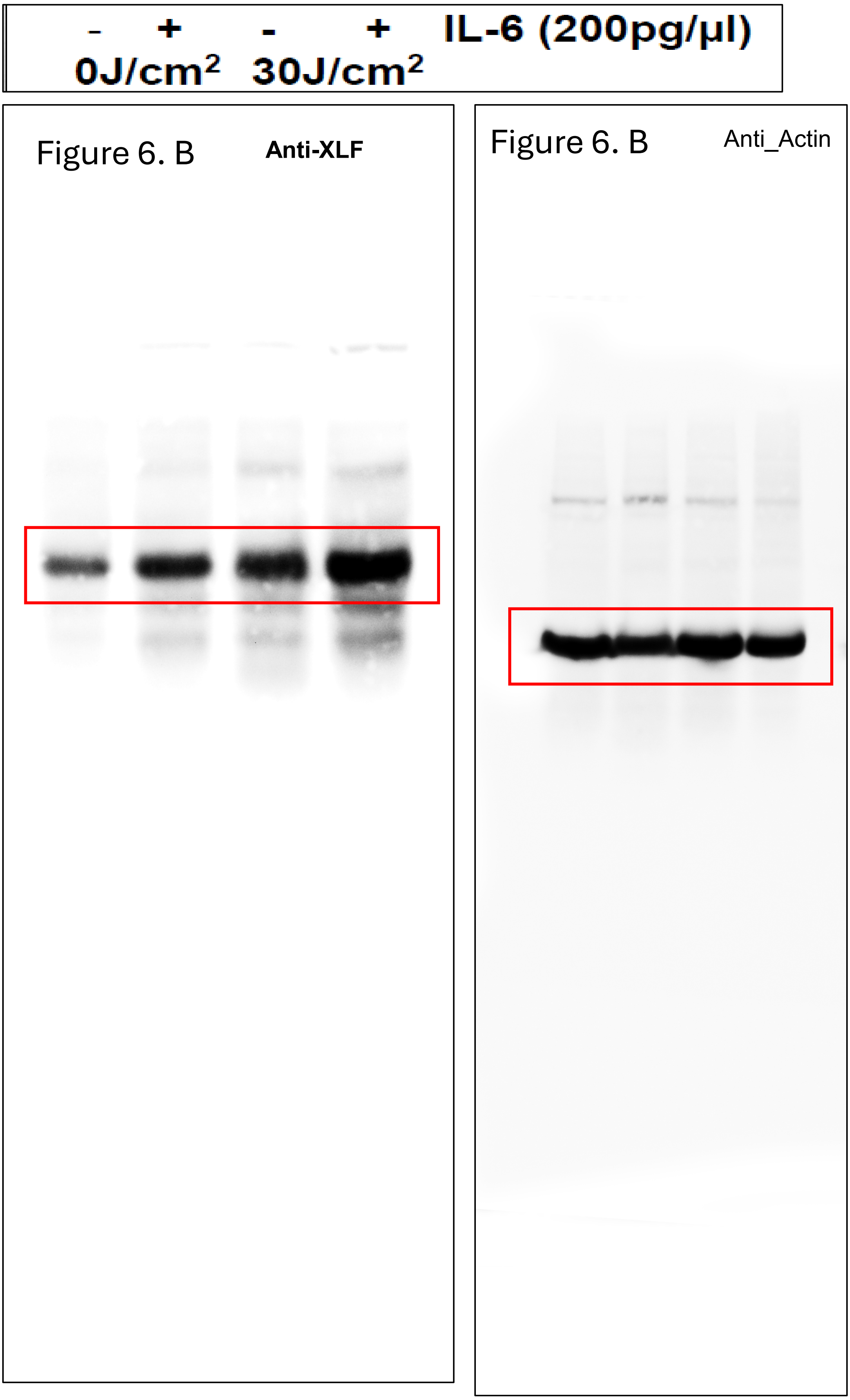

- + - + IL-6 (200pg/µl)
0J/cm2 30J/cm2
Figure 6. B
Anti-XLF
Figure 6. B
Anti_Actin
